# Testing for complete spatial randomness on three dimensional bounded convex shapes^☆^

**DOI:** 10.1016/j.spasta.2020.100489

**Published:** 2021-03

**Authors:** Scott Ward, Edward A.K. Cohen, Niall Adams

**Affiliations:** a Department of Mathematics, Imperial College London, South Kensington, London, SW7 2AZ, United Kingdom; b Data Science Institute, Imperial College London, South Kensington, London, SW7 2AZ, United Kingdom

**Keywords:** Complete spatial randomness, Convex shapes, Functional summary statistics, Poisson point processes

## Abstract

There is currently a gap in theory for point patterns that lie on the surface of objects, with researchers focusing on patterns that lie in a Euclidean space, typically planar and spatial data. Methodology for planar and spatial data thus relies on Euclidean geometry and is therefore inappropriate for analysis of point patterns observed in non-Euclidean spaces. Recently, there has been extensions to the analysis of point patterns on a sphere, however, many other shapes are left unexplored. This is in part due to the challenge of defining the notion of *stationarity* for a point process existing on such a space due to the lack of rotational and translational isometries. Here, we construct functional summary statistics for Poisson processes defined on convex shapes in three dimensions. Using the Mapping Theorem, a Poisson process can be transformed from any convex shape to a Poisson process on the unit sphere which has rotational symmetries that allow for functional summary statistics to be constructed. We present the first and second order properties of such summary statistics and demonstrate how they can be used to construct a test statistics to determine whether an observed pattern exhibits complete spatial randomness or spatial preference on the original convex space. We compare this test statistic with one constructed from an analogue L-function for inhomogeneous point processes on the sphere. A study of the Type I and II errors of our test statistics are explored through simulations on ellipsoids of varying dimensions.

## 1 Introduction

Research in spatial statistics has predominantly concentrated on the development of theory and methodology for point processes on $R^{d}$, with a significant focus on planar ($R^{2}$) and spatial ($R^{3}$) data. Point processes existing on non-Euclidean spaces, however, are still relatively under-explored. Recently, with the advent of spatial data on a global scale, and modelling Earth as a sphere, there have been important developments in the theory and analysis of point processes on the surface of d−1 dimensional unit spheres, $S^{d-1}\subset R^{d}$ (Lawrence et al., 2016, Møller and Rubak, 2016, Robeson et al., 2014). Yet patterns can still arise for which these methodologies are inappropriate as they lie on other bounded metric spaces that deviate significantly from $S^{d-1}$. For example, microbiologists are concerned with the spatial arrangement of lipids and proteins on the cellular membranes of microorganisms that are not adequately modelled by spheres. In the case of bacteria, ellipsoids or capsules (a cylinder with two hemispherical caps placed at each end) are far more appropriate candidate surfaces. Recent advances in 3D super-resolution imaging techniques (e.g. Cabriel et al., 2019, Gustavsson et al., 2018) output point patterns of this type, and there is a demand for the correct statistical procedures to analyse them.

Key to the statistical analysis of spatial data is the ability to form functional summary statistics from an observed pattern, primarily for performing exploratory data analysis and testing for complete spatial randomness (CSR). On $R^{d}$ and $S^{d-1}$, there exists an infinite number of isometries, allowing for the notions of stationarity and isotropy to be well defined, which in turn allows for well defined functional summary statistics. However, on the surface of an arbitrary convex shape $D\subset R^{3}$, the set of available isometries is finite, and thus defining stationarity, isotropy, and summary statistics directly on D is non-trivial. Building on the current literature for spherical point patterns, in particular the discussion of inhomogeneous point processes on a sphere by both Lawrence et al. (2016) and Møller and Rubak (2016), we show that it is possible to construct functional summary statistics for point processes on the surface of arbitrary convex shapes in $R^{3}$, with our primary interest being to test for CSR.

Our approach is to map the point pattern from an arbitrary convex shape D onto $S^{2}$. For any Poisson process on D, the Mapping Theorem (Kingman, 1993) determines that the mapped process on $S^{2}$ remains Poisson with the intensity function dependent on the mapping. By working on $S^{2}$, we operate on a space that is more amenable to constructing summary statistics. These then allow us to test for CSR on D. The functional summary statistics we develop are based on the inhomogeneous counterparts of typical functional summary statistics already established in the spatial statistics literature. In particular, we focus on the inhomogeneous K-function, first discussed by Baddeley et al. (2000) for $R^{d}$ and later extended to $S^{d-1}$ by Lawrence et al. (2016) and Møller and Rubak (2016). Furthermore, we also construct the empty-space function, F, spherical contact distribution, H, and J-function (the ratio of the H- and F-functions) for point processes on arbitrary convex shapes by extending the inhomogeneous definitions of van Lieshout (2011) from $R^{d}$ to $S^{2}$.

Section 2 introduces the notation used throughout this work and formally states the hypothesis for testing CSR on an arbitrary convex shape. Section 3 discusses functional summary statistics on $S^{2}$, key for the construction of summary statistics on more general bounded subsets of $R^{3}$, and the impracticalities of attempting to define functional summary statistics directly on D. Section 4 extends the inhomogeneous F-, H-, and J-functions from $R^{d}$ (van Lieshout, 2011) to $S^{2}$. Section 5 describes the construction of functional summary statistics on bounded subsets of $R^{3}$ for Poisson processes, discussing their first and second order properties in the event that the intensity function is known. Section 6 provides two worked examples constructing functional summary statistics for realisations of a Poisson process observed on a cube and an ellipsoid. Section 7 discusses how regular and cluster processes can be detected based on the deviations of the empirical functional summary statistics. Section 8 describes estimation procedures for the functional summary statistics when the intensity function is unknown and we propose two test statistics for CSR. Finally in Section 9 we conduct empirical power tests using Monte Carlo simulations to explore the properties of our proposed test statistics.

## 2 Preliminaries

In this section we outline the necessary spatial theory and notation used throughout this work. We start by introducing the notion of a bounded convex space in $R^{3}$ and then define what it means for a point process to lie on such a surface. We end with the statement of the problem that this work is primarily focused on.

### 2.1 Notation

Let $x\in R^{3}$ such that $x={\left(x_{1},x_{2},x_{3}\right)}^{T}$ and define $\|x\|={\left(x_{1}^{2}+x_{2}^{2}+x_{3}^{2}\right)}^{1∕2}$ to be the Euclidean norm with the origin of $R^{3}$ denoted as $0={\left(0,0,0\right)}^{T}$. Denote a subset of $R^{3}$ as $D=\left\{x\in R^{3}:g\left(x\right)=0\right\}$, where $g:R^{3}\mapsto R$. We also suppose that D is compact (i.e. closed and bounded) and call g the level-set function of D. Define the set $D_{int}=\left\{x\in R^{3}:g\left(x\right)<0\right\}$, i.e. the boundary of $D_{int}$ is D and we refer to $D_{int}$ as the interior of D. The set $D_{int}$ is said to be convex if and only if for all $x,y\in D_{int}$ such that x≠y then $\left\{z\in R^{3}:z=x+\gamma \left(y-x\right),\;\gamma \in \left(0,1\right)\right\}\in D_{int}$. We thus define D to be convex if its interior, $D_{int}$, is convex. Examples of bounded convex sets of $R^{3}$ are spheres, ellipsoids, and cubes. Further for any bounded convex set D with level-set function g, we will also define $\overset{\sim }{g}$ which rearranges g(x)=0, such that $x_{3}=\overset{\sim }{g}\left(x_{1},x_{2}\right)$, i.e. we write $x_{3}$ as a function of $x_{1}$ and $x_{2}$. It may not always be possible to find $\overset{\sim }{g}$ explicitly since, as defined previously, it may be the case that the resultant $\overset{\sim }{g}$ is not a proper function. This issue can be rectified by partitioning D appropriately. For example take the case of a sphere with radius 1, then $g\left(x\right)=x_{1}^{2}+x_{2}^{2}+x_{3}^{2}-1$, hence $\overset{\sim }{g}\left(x_{1},x_{2}\right)=\pm {\left(1-x_{1}^{2}-x_{2}^{2}\right)}^{1∕2}$, which is not a proper function. In this case we partition the region D into the regions $x_{3}\geq 0$ and $x_{3}<0$. Then for $x_{3}\geq 0$, we define $\overset{\sim }{g}\left(x_{1},x_{2}\right)=+{\left(1-x_{1}^{2}-x_{2}^{2}\right)}^{1∕2}$ and $x_{3}<0$, $\overset{\sim }{g}\left(x_{1},x_{2}\right)=-{\left(1-x_{1}^{2}-x_{2}^{2}\right)}^{1∕2}$. For any bounded convex sets, D, we also define its geodesic as the shortest path between two points x,y∈D such that every point in the path is also an element of D and denote the geodesic distance by $d:D\times D\mapsto R_{+}$, where $R_{+}$ is the positive real line including 0, thus (D,d(⋅,⋅)) is a metric space. Additionally, we will frequently need to evaluate integrals over D, which can be done using its infinitesimal area element defined as,

$$dD=\sqrt{1+{\left(\frac{\partial \overset{\sim }{g}}{\partial x_{1}}\right)}^{2}+{\left(\frac{\partial \overset{\sim }{g}}{\partial x_{2}}\right)}^{2}}dx_{1}dx_{2}.$$

We assume that these convex subsets of $R^{3}$ are defined such that the origin is inside D, that is $0\in D_{int}$, we then say the space D is centred. Our methodology can easily be adapted for non-centred spaces by making the appropriate translations to bring the origin inside D.

Following the notation of Møller and Waagepetersen (2004), we define $\lambda_{D}\left(x\right)$ as the Lebesgue measure restricted to the surface of the convex shape D. Consider point processes which lie on some bounded convex metric space (D,d(⋅,⋅)). We define the notation $A_{K}=A\cap K$, A,K⊆D. This nomenclature is often used when the set A has finite cardinality and K is any subset of D. The cardinality of a set is denoted by |⋅|. Further define $B_{D}\left(x,r\right)=\left\{y\in D,d\left(x,y\right)\leq r\right\}$ and the set $N_{lf}=\left\{A\subset D:\left|A_{K}\right|<\infty ,K\subseteq D\right\}$ where K is any subset of D. In other words, $N_{lf}$ is the set of subsets of D that have finite cardinality. To distinguish between points in a point process X and any point in the space D, we shall refer to elements of our point process x∈X as *events* whilst retaining the term *point* for any point in D. We consider point processes which are *locally finite* and *simple*. A point process, X, lying on D is said to be locally finite, if for any bounded set K⊆D, the number of events of X in K is finite almost surely, i.e. $X\in N_{lf}$ almost surely. A simple point process is one in which no coincident events exist almost surely, in other words if $x_{i},x_{j}\in X$ such that i≠j then $x_{i}\neq x_{j}$. We also define the counting measure of X as $N_{X}\left(K\right)=\left|X\cap K\right|=\left|X_{K}\right|$. We denote the reduced Palm distribution of the point process X by $P_{X_{x}^{!}}$ and define $X_{x}^{!}$ to be the point process following this density, referring to this as the reduced Palm process (Møller and Waagepetersen, 2004). For a point process X on D we define the *intensity measure* as the expected number of events of X for any K⊆D, i.e. $\mu \left(K\right)=E\left[N_{X}\left(K\right)\right]$, whilst the *intensity function* ρ(x) for all x∈D, if it exists, is given by,

$$\mu \left(K\right)=\int_{K}\rho \left(x\right)\lambda_{D}\left(dx\right),$$

where $\rho \left(x\right)\lambda_{D}\left(dx\right)$ can be interpreted heuristically as the probability of an event of X being in the infinitesimal area $\lambda_{D}\left(dx\right)$.

We also define, for n∈N, $\alpha^{\left(n\right)}\left(K_{1},\ldots ,K_{n}\right)=E\sum_{x_{1},\ldots ,x_{n}\in X}^{\neq }1\left[x_{1}\in K_{1},\ldots ,x_{n}\in K_{n}\right]$ as the n*th-order factorial moment measures* where the summation is taken over pairwise distinct sets of $\left\{x_{1},\ldots ,x_{n}\right\}$. Further we shall assume there exists $\rho^{\left(n\right)}\left(x_{1},\ldots ,x_{n}\right)$ such that,

(1) $$\alpha^{\left(n\right)}\left(K_{1},\ldots ,K_{n}\right)=\int_{K_{1}}\cdots \int_{K_{n}}\rho^{\left(n\right)}\left(x_{1},\ldots ,x_{n}\right)\lambda_{D}\left(dx_{1}\right)\cdots \lambda_{D}\left(dx_{n}\right),$$

where $K_{1},\ldots ,K_{n}\subseteq D$. We can interpret $\rho {\left(x_{1},\ldots ,x_{n}\right)}^{\left(n\right)}\lambda_{D}\left(dx_{1}\right)\cdots \lambda_{D}\left(dx_{n}\right)$ as the probability that events of X lie jointly in the infinitesimal areas $\lambda_{D}\left(dx_{i}\right),\;i=1,\ldots ,n$ and call $\rho^{\left(n\right)}$ the n*th-order factorial moment density*. Notice that $\alpha^{\left(1\right)}=\mu$, and $\rho^{\left(1\right)}=\rho$. The *pair correlation function* is defined as,

$$h\left(x,y\right)=\frac{\rho^{\left(2\right)}\left(x,y\right)}{\rho \left(x\right)\rho \left(y\right)},$$

where it is taken that division by 0 results in the pair correlation function equalling 0.

A useful alternative to the nth order product intensities are the n*th order correlation functions* (van Lieshout, 2011). They are recursively defined for n∈N, based on product densities, with $\xi_{1}=1$ and

$$\frac{\rho^{\left(n\right)}\left(x_{1},\ldots ,x_{n}\right)}{\rho \left(x_{1}\right)\cdots \rho \left(x_{n}\right)}=\overset{n}{\underset{k=1}{\sum }}\underset{D_{1},\ldots ,D_{k}}{\sum }\xi_{\left|D_{1}\right|}\left(x_{D_{1}}\right)\cdots \xi_{\left|D_{k}\right|}\left(x_{D_{k}}\right),$$

where the final sum ranges over all partitions $\left\{D_{1},\ldots ,D_{k}\right\}$ of {1,…,n} in k non-empty, disjoint sets, $x_{D_{j}}=\left\{x_{i}:i\in D_{j}\right\},\;j=1,\ldots ,k$, and $x_{i}\in S^{2}$ (van Lieshout, 2011). Further, we define the *generating functional* (Møller and Waagepetersen, 2004) of a point process X as

$$G_{X}\left(u\right)=E\underset{x\in X}{\prod }u\left(x\right),$$

for a function u:D↦[0,1], which are also useful when discussing the F-, H-, and J-functions in Section 4.

We define a Poisson process on D identically to one on $R^{2}$. Let X be a point process on D such that $N_{X}\left(D\right)∼\text{Poisson}\left(\mu \left(D\right)\right)$ where,

$$\mu \left(B\right)=\int_{B}\rho \left(x\right)\lambda_{D}\left(dx\right),\;\text{for }B\subseteq D,$$

where μ(D)<∞. Then given $N_{X}\left(D\right)=n$, $x_{i}\in X,\;i=1,\ldots ,n$ are independent and identically distributed across D with density proportional to ρ(x). We say that X is a Poisson process on D with intensity function $\rho :D\mapsto R_{+}$. When $\rho \in R_{+}$ is constant we say the process is homogeneous Poisson or CSR.

### 2.2 Statement of the problem

We are now in a position to formally state the hypothesis of CSR we are interested in and for which this work provides an approach to testing.

Let X be a spatial point process, such that g(X)=0 where g(X) is a notational convenience for g(x)=0, for all x∈X and g is the level set for the convex shape D. From a realisation of X we wish to conduct the following hypothesis test,

$$H_{0}:X\text{ is CSR on }D\;\;\text{vs.}\;\;H_{1}:X\text{ is not CSR on }D.$$

## 3 Summary statistics on $S^{2}$ and the impracticalities of defining functional summary statistics on general convex shapes

To analyse point patterns on a convex shape D, we will be required to map the point pattern onto the unit sphere $S^{2}$; we use the term spheroidal point process to denote a point process that resides on $S^{2}$. Hence it is necessary to discuss functional summary statistics in this space. We also explain why it is non trivial to construct analogous functional summary statistics directly on D, motivating a need for new methodology in order to test patterns which arise on such surfaces.

### 3.1 Summary statistics on $S^{2}$

Mentioned in passing by Ripley (1977), spherical point patterns only garnered interest over the past five years (Lawrence et al., 2016, Møller and Rubak, 2016, Robeson et al., 2014). Robeson et al. (2014) show that, on a sphere of radius R, the spherical K-function for a homogeneous Poisson process is $K\left(r\right)=2\pi R^{2}\left(1-\cos\left(r∕R\right)\right)$, where r is the geodesic distance from an arbitrary event of the process. Building on this, both Lawrence et al. (2016) and Møller and Rubak (2016) define a range of typical functional summary statistics for isotropic (rotationally invariant) point processes, including the empty-space and nearest-neighbour distributions. They also extend the K-function to the class of inhomogeneous point processes that have rotationally invariant pair correlation functions. This is analogous to the inhomogeneous extension given by Baddeley et al. (2000) for point processes on $R^{d}$.

The geodesic on $S^{2}$ is commonly referred to as the great circle distance and for two points $x,y\in S^{2}$ has analytic form $d\left(x,y\right)=\cos^{-1}\left(x\cdot y\right)$, where x⋅y is the dot product between vectors $x,y\in S^{2}$. We define a point process X on $S^{2}$ to be isotropic if its distribution is invariant under rotations, i.e. $X\overset{d}{=}OX$, for all O∈O(3), where O(3) is the set of orthogonal 3 × 3 matrices (Møller and Rubak, 2016). Here we use $\overset{d}{=}$ to denote *equal in distribution*. Such an isotropic process has constant intensity function $\rho \in R_{+}$.

Functional summary statistics are frequently employed for both exploratory data analysis and model fitting, playing a pivotal role in the early stages of any in depth investigation of an observed point pattern. In the homogeneous case, let X be an isotropic spheroidal point process with constant intensity function $\rho \in R_{+}$ then the F-, H-, J-, and K-functions are defined as,

(2) $$F\left(r\right)=P\left(X_{B\left(o,r\right)}\neq 0̸\right)$$

(3) $$H\left(r\right)=P\left(X_{o,B\left(o,r\right)}^{!}\neq 0̸\right)$$

(4) $$J\left(r\right)=\frac{1-H\left(r\right)}{1-F\left(r\right)}$$

(5) $$K\left(r\right)=\frac{1}{\rho }E\underset{x\in X_{o}^{!}}{\sum }1\left[d\left(x,o\right)\leq r\right],$$

where r∈[0,π] and $o={\left(0,0,1\right)}^{T}$ is defined as the origin of $S^{2}$. Estimators of these functional summary statistics can be used to determine whether the underlying process of an observed point pattern follows a specific distribution. In particular, they can be used to test whether a pattern arises from a CSR process or whether the underlying process exhibits regularity or clustering. A treatment of the standard isotropic functional summary statistics on $S^{2}$ is given in Lawrence et al. (2016) and Møller and Rubak (2016).

It will be necessary for us to consider the inhomogeneous extensions of these functional summary statistics as they will form the foundation for our functional summary statistics for point process on convex bounded shapes in $R^{3}$. We begin by reviewing the inhomogeneous K-function, originally attributed to Baddeley et al. (2000) for a class of inhomogeneous point processes in $R^{d}$, and then extended to non-isotropic point processes on $S^{2}$ by Lawrence et al. (2016) and Møller and Rubak (2016). In Section 4, we will construct the inhomogeneous F-, H-, and J-functions for non-isotropic point processes on $S^{2}$. This builds on the formulation of van Lieshout (2011) for non-stationary point processes in $R^{d}$.

### 3.2 Inhomogeneous K-function

For the extension of the K-function to inhomogeneous processes on $R^{2,3}$, Baddeley et al. (2000) introduce the notion of a point process being *second order intensity reweighted stationary* (SOIRWS). Here we focus on the extension for $S^{2}$ where Lawrence et al. (2016) and Møller and Rubak (2016) define the notion of *second order intensity reweighted isotropic* (SOIRWI). A point process X on $S^{2}$ is said to be SOIRWI if its pair correlation function, h, is rotationally invariant, that is h(x,y)=h(d(x,y)), where d is the great circle distance on $S^{2}$. Lawrence et al. (2016) and Møller and Rubak (2016) define the inhomogeneous K-function for a SOIRWI process as,

$$K_{\text{inhom}}\left(r\right)=\frac{1}{\lambda_{S^{2}}\left(A\right)}E\underset{x,y\in X}{\sum^{\neq }}\frac{1\left[x\in A,O_{x}\left(y\right)\in B_{S^{2}}\left(o,r\right)\right]}{\rho \left(x\right)\rho \left(y\right)},\;0\leq r\leq \pi ,$$

where $O_{x}:S^{2}\mapsto S^{2}$ is a rotation that takes x to o. Here, $K_{\text{inhom}}$ is independent of the choice of $A\subseteq S^{2}$ with $\lambda_{S^{2}}\left(A\right)>0$, and by convention a∕0=0, for a≥0. For a Poisson process it is easy to show that $K_{\text{inhom}}\left(r\right)=2\pi \left(1-\cos\left(r\right)\right)$. Therefore the K-function is the same for all Poisson processes regardless of whether the intensity function is constant or not (Møller and Rubak, 2016).

Both Lawrence et al. (2016) and Møller and Rubak (2016) propose the following estimator for $K_{\text{inhom}}$ for a fully observed point pattern on $S^{2}$,

(6) $${\overset{ˆ}{K}}_{\text{inhom}}\left(r\right)=\frac{1}{4\pi }\underset{x,y\in X}{\sum^{\neq }}\frac{1\left[d\left(x,y\right)\leq r\right]}{\rho \left(x\right)\rho \left(y\right)},$$

which is unbiased if ρ is known. In the more likely event that ρ is unknown, Lawrence et al. (2016) and Møller and Rubak (2016) suggest using a plugin estimator for ρ(x)ρ(y).

### 3.3 Impracticalities of defining functional summary statistics directly on D

We now explain the subtle reasoning as to why constructing functional summary statistics directly on D is not a trivial extension from $S^{2}$. The definitions given by Eqs. (2)–(5) are well defined when considering stationary or isotropic point processes on $R^{d}$ or $S^{2}$ respectively. This is because the symmetries of the space admit well defined notions of stationarity/isotropy based on translations and rotations. Since an arbitrary convex space D does not, in general, have isometries these notions of stationarity/isotropy cannot be well defined. Therefore, defining functional summary statistics analogous to (2)–(5) is not possible.

Further, we also argue that we cannot define a point process to be SOIRWI on D. On $S^{2}$ being SOIRWI is equivalent to having a rotationally invariant pair correlation function. We may be tempted to equivalently define a point process to be SOIRWI on D if it has an invariant form for its pair correlation function. In particular this would make sense for a Poisson process on D as it would have pair correlation function, h(x)=1 for all x∈D. Closer inspection though leads us to conclude that this is not an appropriate definition for SOIRWI on D. Based on Møller and Waagepetersen (2004, Definition 4.5, p. 32) we can take a point process X with intensity function $\rho :S^{2}\mapsto R_{+}$ as being SOIRWI on $S^{2}$ if the measure,

(7) $$K\left(B\right)=\frac{1}{\lambda_{S^{2}}\left(A\right)}E\underset{x,y\in X}{\sum^{\neq }}\frac{1\left[x\in A,O_{x}\left(y\right)\in B\right]}{\rho \left(x\right)\rho \left(y\right)},\;B\subseteq S^{2},$$

does not depend on the choice of $A\subseteq S^{2}$ for 0<|A|<∞, where we take a∕0=0. K is then called the *second order reduced moment measure*. If the pair correlation function exists and is invariant under rotations, then by the Campbell–Mecke Theorem (Møller and Waagepetersen, 2004) it follows that

$$K\left(B\right)=\int_{B}h\left(d\left(o,x\right)\right)\lambda_{S^{2}}\left(dx\right),\;B\subseteq S^{2}.$$

Thus on $S^{2}$ a point process is SOIRWI if h is invariant under rotations. Eq. (7) implicitly depends on rotations $O_{x}\left(y\right)$. If we now consider a point process on D, we cannot construct the second order reduced moment measure as, in general, we do not have an analogous isometry. This in turn means that we cannot define SOIRWI directly on D based on an invariance of the pair correlation function.

Moreover, for a point process on $S^{2}$, consider the more specific case when $B=B_{S^{2}}\left(o,r\right),r>0$ in (7). This is identically the inhomogeneous K-function. The indicator function of (7) is still well-defined in the case of $S^{2}$ such that we are counting the events of X∖{x} that are at most a distance r from x∈X. This same intuition could not equivalently be applied to point processes on a convex shape as the ball of radius r from a point x on D also depends on x, i.e. $B_{D}\left(x,r\right)\subset D$ is different for each x∈D. Thus it is not possible to directly define an inhomogeneous K-function on D.

## 4 Extending the inhomogeneous F-, H-, and J-functions to $S^{2}$

On $R^{d}$, in the stationary case, it can be shown that the F-, and H-functions have infinite series representations (White, 1979), and further work by van Lieshout (2006) also gives an infinite series representation for the J-function based on the nth-order correlation functions. Theorem 1 gives an infinite series representation when the nth-order reduced factorial moment measure of all n exists, similar to (White, 1979) but where the underlying space is $S^{2}$.

Theorem 1 *Let* X *be an isotropic spheroidal point process with constant intensity function* ρ*. Further we assume the existence of all* n*th-order factorial moment measures for both* X *and its reduced Palm process,* $X_{x}^{!}$*. Then the* F*- and* H*-functions have the following series representation,*

$$F\left(r\right)=-\overset{\infty }{\underset{n=1}{\sum }}\frac{{\left(-1\right)}^{n}}{n!}\alpha^{\left(n\right)}\left(B_{S^{2}}\left(o,r\right),\ldots ,B_{S^{2}}\left(o,r\right)\right)$$

$$H\left(r\right)=-\overset{\infty }{\underset{n=1}{\sum }}\frac{{\left(-1\right)}^{n}}{n!}\alpha_{o}^{!\left(n\right)}\left(B_{S^{2}}\left(o,r\right)\ldots ,B_{S^{2}}\left(o,r\right)\right)$$

*where* $\alpha^{\left(n\right)}$ *and* $\alpha_{x}^{!\left(n\right)}$ *are the factorial moment measure for* X *and* $X_{x}^{!}$ *and* $B_{S^{2}}\left(o,r\right)$ *is the spherical cap of radius* r *at the origin* $o\in S^{2}$*. These representations hold provided the series is absolutely convergent, that is if* $\lim_{n\to \infty }\left|a_{n+1}∕a_{n}\right|<1$ *or* ${lim sup}_{n\to \infty }{\left(\left|a_{n}\right|\right)}^{1∕n}<1$*, where* $a_{n}=\left({\left(-1\right)}^{n}∕n!\right)\alpha^{\left(n\right)}\left(B_{S^{2}}\left(o,r\right),\ldots ,B_{S^{2}}\left(o,r\right)\right)$ *for the* F*-function or* $a_{n}=\left({\left(-1\right)}^{n}∕n!\right)$ $\alpha_{o}^{!\left(n\right)}\left(B_{S^{2}}\left(o,r\right)\ldots ,B_{S^{2}}\left(o,r\right)\right)$ *for the* H*-function.*

Proof See Theorem 1, Section S1 of the Supplementary Material. □

The following corollary reduces the representations for the F-, and H-function for when the nth-order product density exist. These representations are those used by van Lieshout (2011).

Corollary 1 **White, 1979**

*Under the same assumptions as* Theorem 1*, let* X *be an isotropic spheroidal point process with constant intensity function* ρ*. Further we assume the existence of all* n*th-order product intensities for both* X *and its reduced Palm process,* $X_{x}^{!}$*. Then the* F*- and* H*-functions have the following series representation,*

$$F\left(r\right)=-\overset{\infty }{\underset{n=1}{\sum }}\frac{{\left(-1\right)}^{n}}{n!}\int_{B_{S^{2}}\left(o,r\right)}\cdots \int_{B_{S^{2}}\left(o,r\right)}\rho^{\left(n\right)}\left(x_{1},\ldots ,x_{n}\right)\lambda_{S^{2}}\left(dx_{1}\right)\cdots \lambda_{S^{2}}\left(dx_{n}\right)$$

$$H\left(r\right)=-\overset{\infty }{\underset{n=1}{\sum }}\frac{{\left(-1\right)}^{n}}{n!}\int_{B_{S^{2}}\left(o,r\right)}\cdots \int_{B_{S^{2}}\left(o,r\right)}\frac{\rho^{\left(n+1\right)}\left(o,x_{1},\ldots ,x_{n}\right)}{\rho }\lambda_{S^{2}}\left(dx_{1}\right)\cdots \lambda_{S^{2}}\left(dx_{n}\right)$$

*provided the series is absolutely convergent, where* $B_{S^{2}}\left(o,r\right)$ *is the spherical cap of radius* r *at the origin* $o\in S^{2}$*.*

Proof See Corollary 1, Section S1 of the Supplementary Material. □

Adapting the work of van Lieshout (2011), the J-function for an isotropic spheroidal point process, based on the series for the F-, and H-function given by Theorem 1, has the following infinite series representation

$$J\left(r\right)=1+\overset{\infty }{\underset{n=1}{\sum }}\frac{{\left(-\rho \right)}^{n}}{n!}J_{n}\left(r\right),\;0\leq r\leq \pi ,$$

where $J_{n}\left(r\right)=\int_{B_{S^{2}}\left(o,r\right)}\cdots \int_{B_{S^{2}}\left(o,r\right)}\xi^{\left(n+1\right)}\left(o,x_{1},\ldots ,x_{n}\right)\lambda_{S^{2}}\left(dx_{1}\right)\cdots \lambda_{S^{2}}\left(dx_{n}\right)$, and $B_{S^{2}}\left(o,r\right)$ is the spherical cap at the origin $o\in S^{2}$.

In order to define the inhomogeneous F-, H-, and J-function we first define the notion of *iterative reweighted moment isotropic* (IRWMI) for a class of non-isotropic spheroidal point processes, similar to the notion of *iterative reweighted moment stationary* in $R^{2,3}$ (van Lieshout, 2011).

Definition 1 A spheroidal point process X is said to be IRWMI if, for all n∈N, the nth-order correlation functions are rotationally invariant. That is $\xi_{n}\left(x_{1},\ldots ,x_{n}\right)=\xi_{n}\left(Ox_{1},\ldots ,Ox_{n}\right)$ for all n∈N and O∈O(3).

Identically to the inhomogeneous J-function in $R^{2,3}$ (van Lieshout, 2011), we define the inhomogeneous J-function on $S^{2}$.

Definition 2 For an IRWMI point process X with intensity function $\rho :S^{2}\mapsto R_{+}$ such that $\overset{¯}{\rho }\equiv \inf_{x\in S^{2}}\rho \left(x\right)>0$,

$$J_{\text{inhom}}\left(r\right)=1+\overset{\infty }{\underset{n=1}{\sum }}\frac{{\left(-\overset{¯}{\rho }\right)}^{n}}{n!}J_{n}\left(t\right),\;0\leq r\leq \pi$$

where $J_{n}\left(r\right)=\int_{B_{S^{2}}\left(o,r\right)}\cdots \int_{B_{S^{2}}\left(o,r\right)}\xi_{n+1}\left(o,x_{1},\ldots ,x_{n}\right)\lambda_{S^{2}}\left(dx_{1}\right)\cdots \lambda_{S^{2}}\left(dx_{n}\right)$ and the series is absolutely convergent.

Notice that since the point process is IRWMI then the J-function does not depend on the origin o, and furthermore when the point process is isotropic $J_{\text{inhom}}$ collapses down to J since the intensity function is constant. In the context of $R^{d}$, van Lieshout (2011) shows that the inhomogeneous J-function can be written as the ratio of generating functionals of the point process. Here we easily adapt the theorem for IRWMI point processes on $S^{2}$.

Theorem *For all* r∈[0,π] *and* $y\in S^{2}$*,*

$$u_{r}^{y}\left(x\right)=\frac{\overset{¯}{\rho }1\left[O_{y}\left(x\right)\in B_{S^{2}}\left(o,r\right)\right]}{\rho \left(x\right)},\;x\in S^{2},$$

*where* $O_{y}:S^{2}\mapsto S^{2}$ *is a rotation that maps* y *to* o*. Assuming that the series* $\sum_{n=1}^{\infty }\frac{{\overset{¯}{\rho }}^{n}}{n!}\int_{B_{S^{2}}\left(o,r\right)}\cdots \int_{B_{S^{2}}\left(o,r\right)}\frac{\rho^{\left(n\right)}\left(x_{1},\ldots ,x_{n}\right)}{\rho \left(x_{1}\right)\cdots \rho \left(x_{n}\right)}$ $\lambda_{S^{2}}\left(dx_{1}\right)\cdots \lambda_{S^{2}}\left(dx_{n}\right)$ *is absolutely convergent. Then under the further assumptions associated with the inhomogeneous* J*-function and the existence of all* n*th-order intensity function* $\rho_{y}^{!\left(n\right)}$ *for the reduced Palm distribution* $X_{y}^{!}$*,* $\forall y\in S^{2}$*,*

$$J_{\text{inhom}}\left(r\right)=\frac{G_{y}^{!}\left(1-u_{r}^{y}\right)}{G\left(1-u_{r}^{y}\right)},\;0\leq r\leq \pi ,$$

*for when* $G\left(1-u_{r}^{y}\right)>0$*, where* $G_{y}^{!}$ *and* G *are the generating functionals for* $X_{y}^{!}$ *and* X *respectively.*

Proof See Theorem 1 of van Lieshout (2011). □

From the proof given by van Lieshout (2011), it can be shown that the numerator and denominator do not depend on the arbitrary event or point y respectively. Further, in the case of an isotropic point process the numerator can be shown to be $G_{y}^{!}\left(1-u_{r}^{y}\right)=1-H\left(r\right)$, whilst the denominator is $G\left(1-u_{r}^{y}\right)=1-F\left(r\right)$, and so the F-, and H-functions can be extended to the inhomogeneous case,

$$F_{\text{inhom}}\left(r\right)=1-G\left(1-u_{r}^{y}\right)$$

$$H_{\text{inhom}}\left(r\right)=1-G_{y}^{!}\left(1-u_{r}^{y}\right),$$

where the functions do not depend on the arbitrary event y of the point process.

Similar to van Lieshout (2011) in $R^{2,3}$, we propose the following estimators for the inhomogeneous F-, and H-functions for spheroidal IRWMI point processes as,

(8) $${\overset{ˆ}{F}}_{\text{inhom}}\left(r\right)=1-\frac{\underset{p\in P}{\sum }\underset{x\in X\cap B_{S^{2}}\left(p,r\right)}{\prod }\left(1-\frac{\overset{¯}{\rho }}{\rho \left(x\right)}\right)}{\left|P\right|}$$

(9) $${\overset{ˆ}{H}}_{\text{inhom}}\left(r\right)=1-\frac{\underset{x\in X}{\sum }\underset{y\in \left(X\setminus \left\{x\right\}\right)\cap B_{S^{2}}\left(x,r\right)}{\prod }\left(1-\frac{\overset{¯}{\rho }}{\rho \left(y\right)}\right)}{N_{X}\left(S^{2}\right)},$$

where $P\subseteq S^{2}$ is a finite grid of points. The properties of the ${\overset{ˆ}{F}}_{\text{inhom}}$-function are independent of the choice of P (van Lieshout, 2011). In this work we choose P such that the points on $S^{2}$ are equidistant. van Lieshout (2011) shows that ${\overset{ˆ}{F}}_{\text{inhom}}\left(r\right)$ is unbiased whilst ${\overset{ˆ}{H}}_{\text{inhom}}\left(r\right)$ is ratio-unbiased. Then since ${\overset{ˆ}{F}}_{\text{inhom}}$ is unbiased and ${\overset{ˆ}{H}}_{\text{inhom}}$ is ratio-unbiased, constructing ${\overset{ˆ}{J}}_{\text{inhom}}$ as

(10) $${\overset{ˆ}{J}}_{\text{inhom}}\left(r\right)=\frac{1-{\overset{ˆ}{H}}_{\text{inhom}}\left(r\right)}{1-{\overset{ˆ}{F}}_{\text{inhom}}\left(r\right)},$$

gives a ratio-unbiased estimator for $J_{\text{inhom}}\left(r\right)$.

## 5 Summary statistics for Poisson processes on convex shapes

Here, we construct summary statistics for Poisson processes on general convex shapes. We show that a Poisson process on a general convex shape, D, can be mapped to a Poisson process on a sphere, and then define functional summary statistics for such processes. We discuss properties of these functional summary statistics in the more general setting of inhomogeneous Poisson processes on $S^{2}$.

### 5.1 Mapping from D to $S^{2}$

To circumvent the geometrical restrictions of D we show, in this section, that we can map Poisson processes from D to $S^{2}$ and construct functional summary statistics in this space. Theorem 2 shows that a Poisson process on D can be transformed to a Poisson process on a sphere where we can take advantage of the rotational symmetries. The invariance of Poisson processes between metric spaces is known as the Mapping Theorem (Kingman, 1993). We use the function f(x)=x∕‖x‖ to map point patterns from D to $S^{2}$. Lemma 1 shows that this function is bijective and hence measurable.

Lemma 1 *Let* D *be a convex subset of* $R^{3}$ *such that the origin in* $R^{3}$ *is in the interior of* D*, i.e.* $o\in D_{int}$*. Then the function* $f\left(x\right)=x∕\|x\|,f:D\mapsto S^{2}$ *is bijective.*

Proof See Lemma 1, Section S2 of the Supplementary Material. □

Rather than using the Mapping Theorem (Kingman, 1993), we utilise Proposition 3.1 of Møller and Waagepetersen (2004) to show that mapping a Poisson process from D to $S^{2}$ results in a new Poisson process on $S^{2}$ and also derive the intensity function of the mapped process on $S^{2}$.

Theorem 2 *Let* X *be a Poisson process on an arbitrary bounded convex shape* $D\subset R^{3}$ *with intensity function* $\rho :D\mapsto R_{+}$*. We assume that* $D=\left\{x\in R^{3}:g\left(x\right)=0\right\}$ *where* g(x)=0 *is the level-set function and is defined as,*

$$g\left(x\right)=\left\{\begin{array}{cc} g_{1}\left(x\right)=0, & \;x\in D_{1}\\ & \vdots \\ g_{n}\left(x\right)=0, & \;x\in D_{n} \end{array}\right)$$

*such that* $\cup_{i=1}^{n}D_{i}=D$ *and* $D_{i}\cap D_{j}=0̸,\;\forall i\neq j$*. Let* Y=f(X)*, where* f(x)=x∕‖x‖ *with* $f\left(X\right)=\left\{y\in S^{2}:y=x∕\|x\|,x\in X\right\}$*. Then* Y *is a Poisson process on* $S^{2}$*, with intensity function,*

(11) $$\rho^{*}\left(x\right)=\left\{\begin{array}{cc} \rho \left(f^{-1}\left(x\right)\right)l_{1}\left(f^{-1}\left(x\right)\right) & J_{\left(1,f^{*}\right)}\left(x\right)\sqrt{1-x_{1}^{2}-x_{2}^{2}},\;x\in f\left(D_{1}\right)\\ & \vdots \\ \rho \left(f^{-1}\left(x\right)\right)l_{n}\left(f^{-1}\left(x\right)\right) & J_{\left(n,f^{*}\right)}\left(x\right)\sqrt{1-x_{1}^{2}-x_{2}^{2}},\;x\in f\left(D_{n}\right) \end{array}\right)$$

*where,*

$$x_{3}={\overset{\sim }{g}}_{i}\left(x_{1},x_{2}\right)$$

$$l_{i}\left(x\right)={\left[1+{\left(\frac{\partial {\overset{\sim }{g}}_{i}}{\partial x_{1}}\right)}^{2}+{\left(\frac{\partial {\overset{\sim }{g}}_{i}}{\partial x_{2}}\right)}^{2}\right]}^{\frac{1}{2}}$$

$$J_{\left(i,f^{*-1}\right)}\left(x\right)=\frac{1}{{\left(x_{1}^{2}+x_{2}^{2}+{\overset{\sim }{g}}_{i}^{2}\left(x_{1},x_{2}\right)\right)}^{3}}\det\left[\left(\begin{array}{cc} \overset{2}{\underset{2}{x}}+{\overset{\sim }{g}}_{i}^{2}\left(x_{1},x_{2}\right)-x_{1}{\overset{\sim }{g}}_{i}\left(x_{1},x_{2}\right)\frac{\partial {\overset{\sim }{g}}_{i}}{\partial x_{1}} & -x_{1}\left(x_{2}+{\overset{\sim }{g}}_{i}\left(x_{1},x_{2}\right)\frac{\partial {\overset{\sim }{g}}_{i}}{\partial x_{2}}\right)\\ -x_{2}\left(x_{1}+{\overset{\sim }{g}}_{i}\left(x_{1},x_{2}\right)\frac{\partial {\overset{\sim }{g}}_{i}}{\partial x_{1}}\right) & \overset{2}{\underset{1}{x}}+{\overset{\sim }{g}}_{i}^{2}\left(x_{1},x_{2}\right)-x_{2}{\overset{\sim }{g}}_{i}\left(x_{1},x_{2}\right)\frac{\partial {\overset{\sim }{g}}_{i}}{\partial x_{2}} \end{array}\right)\right]$$

$$J_{\left(i,f^{*}\right)}\left(x\right)=\frac{1}{J_{\left(i,f^{*-1}\right)}\left(f^{-1}\left(x\right)\right)},$$

*where* $f^{-1}$ *is the inverse of* f*,* det(⋅) *is the determinant operator, and* $f^{*}:R^{2}\mapsto R^{2}$ *is the function which maps* $x_{1}\mapsto x_{1}∕\|x\|$ *and* $x_{2}\mapsto x_{2}∕\|x\|$*.*

Proof See Theorem 2, Section S2 of the Supplementary Material. □

To solidify the notation used to describe the space D in Theorem 1, we demonstrate it with a clear example. Let us suppose that D is an ellipsoid with semi-major axis lengths a,b, and c along the x, y and z - axes. Then we define $D_{1}$ to be the $D\cap \left\{x\in R^{3}:x={\left(x_{1},x_{2},x_{3}\right)}^{T}\text{ and }x_{3}\geq 0\right\}$ i.e. the elements of D with non-negative $x_{3}$ component. Similarly we define $D_{2}=D\cap \left\{x\in R^{3}:x={\left(x_{1},x_{2},x_{3}\right)}^{T}\text{ and }x_{3}<0\right\}$. Then using the notation outlined in Section 2.1 we take ${\overset{\sim }{g}}_{1}\left(x\right)=+c\sqrt{1-{\left(x_{1}∕a\right)}^{2}-{\left(x_{2}∕b\right)}^{2}}$ and ${\overset{\sim }{g}}_{2}\left(x\right)=-c\sqrt{1-{\left(x_{1}∕a\right)}^{2}-{\left(x_{2}∕b\right)}^{2}}$.

Remark 1 A notion of bijectivity arises from this theorem. Consider the set of all Poisson processes on D such that their intensity functions exist, label this set $T_{D}$. Also define $T_{S^{2}}$ as all the Poisson processes on $S^{2}$ such that their intensity functions exist. Then for any $X\in T_{D}$ implies that $f\left(X\right)\in T_{S^{2}}$. Similarly by considering the inverse operation $f^{-1}$, which exists by Lemma 1, for all $Y\in T_{S^{2}}$ implies that $f\left(Y\right)\in T_{D}$. Hence the mapping $f:T_{D}\mapsto T_{S^{2}}$ is surjective. By Theorem 2 if X,Y are Poisson processes on D with intensity function $\rho_{X}$ and $\rho_{Y}$ respectively then f(X) and f(Y) are the same Poisson process if and only if $\rho_{X}=\rho_{Y}$ and so the mapping is also injective, and hence bijective. This means that analysis of a Poisson process, X, on D is equivalent to the analysis of f(X) on $S^{2}$.

Remark 2 Further, another useful result which follows directly from Theorem 2 is the construction of approximate Poisson processes on $S^{2}$. More precisely consider a convex surface D for which instead of having a level-set function, g, we have an approximation to the space, for example consider we have a finite piecewise planar approximation to D. Then D can be approximated by $\cup_{i=1}^{n}D_{i}$, where each $D_{i}$ is a planar piece and there are n∈N pieces to the approximation. For each $D_{i}$ the level-set function is $g_{i}\left(x\right)=a_{i}x_{1}+b_{i}x_{2}+c_{i}x_{3}+d_{i}=0$, and we can then use this approximation of D to map a Poisson process on D to $S^{2}$.

### 5.2 Construction of functional summary statistics

We are now in a position to construct functional summary statistics for a Poisson process which lies on some bounded convex space D. Since all Poisson processes on $S^{2}$ are SOIRWI (Møller and Rubak, 2016) and IRWMI (van Lieshout, 2011), the estimators for $F_{\text{inhom}},H_{\text{inhom}},J_{\text{inhom}}$, and $K_{\text{inhom}}$ (see Eqs. (8)–(10), (6) respectively) (van Lieshout, 2011, Møller and Rubak, 2016) can be combined with the mapped intensity function from Theorem 2 to construct estimators as follows,

(12) $${\overset{ˆ}{F}}_{\text{inhom},D}\left(r\right)=1-\frac{\underset{p\in P}{\sum }\underset{x\in Y\cap B_{S^{2}}\left(p,r\right)}{\prod }\left(1-\frac{\overset{¯}{\rho^{*}}}{\rho^{*}\left(x\right)}\right)}{\left|P\right|}$$

(13) $${\overset{ˆ}{H}}_{\text{inhom},D}\left(r\right)=1-\frac{\underset{x\in Y}{\sum }\underset{y\in \left(Y\setminus \left\{x\right\}\right)\cap B_{S^{2}}\left(x,r\right)}{\prod }\left(1-\frac{\overset{¯}{\rho^{*}}}{\rho^{*}\left(y\right)}\right)}{N_{Y}\left(S^{2}\right)}$$

(14) $${\overset{ˆ}{J}}_{\text{inhom},D}\left(r\right)=\frac{1-{\overset{ˆ}{H}}_{\text{inhom},D}\left(r\right)}{1-{\overset{ˆ}{F}}_{\text{inhom},D}\left(r\right)}$$

(15) $${\overset{ˆ}{K}}_{\text{inhom},D}\left(r\right)=\frac{1}{4\pi }\underset{x,y\in Y}{\sum^{\neq }}\frac{1\left[d\left(x,y\right)\leq r\right]}{\rho^{*}\left(x\right)\rho^{*}\left(y\right)},$$

where X is a Poisson process on D with intensity function ρ, Y=f(X) is the mapped Poisson process onto $S^{2}$, $\rho^{*}$ is given by (11) and $\overset{¯}{\rho^{*}}=\inf_{x\in S^{2}}\rho^{*}\left(x\right)$. In the event that $\rho :D\mapsto R_{+}$ is unknown and therefore $\rho^{*}$ is unknown, nonparametric plug-in estimates of $\rho^{*}$ can be constructed on $S^{2}$ (Lawrence et al., 2016, Møller and Rubak, 2016).

### 5.3 Properties of functional summary statistics

Consider the general case of all Poisson processes on $S^{2}$. Theorem 3 gives the expectations of ${\overset{ˆ}{F}}_{\text{inhom}}\left(r\right)$, ${\overset{ˆ}{H}}_{\text{inhom}}\left(r\right)$, and ${\overset{ˆ}{K}}_{\text{inhom}}\left(r\right)$. We restate the mean of ${\overset{ˆ}{K}}_{\text{inhom}}$ (Lawrence et al., 2016, Møller and Rubak, 2016) and adapt the proof to Proposition 1 in van Lieshout (2011) for $R^{d}$, to show that ${\overset{ˆ}{F}}_{\text{inhom}}$ is unbiased and ${\overset{ˆ}{H}}_{\text{inhom}}$ is ratio unbiased for $S^{2}$. In addition we also provide the expectation of ${\overset{ˆ}{H}}_{\text{inhom}}\left(r\right)$.

Theorem 3 *Let* X *be a spherical Poisson process on* $S^{2}$ *with known intensity function* $\rho :S^{2}\mapsto R_{+}$*, such that* $\overset{¯}{\rho }=\inf_{x\in S^{2}}\rho \left(x\right)>0$*. Then the estimators for* ${\overset{ˆ}{F}}_{\text{inhom}}\left(r\right)$*, and* ${\overset{ˆ}{K}}_{\text{inhom}}\left(r\right)$ *are unbiased whilst* ${\overset{ˆ}{H}}_{\text{inhom}}\left(r\right)$ *is ratio-unbiased. More precisely,*

$$E\left[{\overset{ˆ}{F}}_{\text{inhom}}\left(r\right)\right]=1-\exp\left(-\overset{¯}{\rho }2\pi \left(1-\cos r\right)\right)$$

$$E\left[{\overset{ˆ}{H}}_{\text{inhom}}\left(r\right)\right]=1-\frac{\exp\left(-\overset{¯}{\rho }2\pi \left(1-\cos r\right)\right)-\exp\left(-\mu \left(S^{2}\right)\right)}{1-\frac{\overset{¯}{\rho }2\pi \left(1-\cos r\right)}{\mu \left(S^{2}\right)}}$$

$$E\left[{\overset{ˆ}{K}}_{\text{inhom}}\left(r\right)\right]=2\pi \left(1-\cos r\right),$$

*where* r∈[0,π]*, and* $\overset{¯}{\rho }=\inf_{x\in S^{2}}\rho \left(x\right)>0$*. Further by unbiasedness and ratio-unbiasedness of* ${\overset{ˆ}{F}}_{\text{inhom}}\left(r\right)$ *and* ${\overset{ˆ}{H}}_{\text{inhom}}\left(r\right)$*, respectively, we immediately have ratio-unbiasedness of* ${\overset{ˆ}{J}}_{\text{inhom}}\left(r\right)$*.*

Proof See Lawrence et al. (2016) for treatment of ${\overset{ˆ}{K}}_{\text{inhom}}\left(r\right)$. Results for ${\overset{ˆ}{F}}_{\text{inhom}}\left(r\right)$ and ${\overset{ˆ}{H}}_{\text{inhom}}\left(r\right)$ follow from a trivial adaptation of the proof for Proposition 1 in van Lieshout (2011). For the expectation of ${\overset{ˆ}{H}}_{\text{inhom}}\left(r\right)$ see Theorem 3, Section S3 of the Supplementary Material. □

Theorem 3 shows that ${\overset{ˆ}{H}}_{\text{inhom}}\left(r\right)$ is a biased estimator for $H_{\text{inhom}}\left(r\right)$. Although biased it can be bounded.

Corollary 2 *With the same assumptions as* Theorem 3*, let* X *be a spherical Poisson process on* $S^{2}$ *with intensity function* $\rho :S^{2}\mapsto R_{+}$*. Defining* $\overset{¯}{\rho }=\inf_{x\in S^{2}}\rho \left(x\right)$*, the bias of the estimator* ${\overset{ˆ}{H}}_{\text{inhom}}\left(r\right)$ *is bounded by*

$$\left|\text{Bias}\left({\overset{ˆ}{H}}_{\text{inhom}}\left(r\right)\right)\right|\leq \exp\left(-\mu \left(S^{2}\right)\right)\leq \exp\left(-4\pi \overset{¯}{\rho }\right),$$

*for all* r∈[0,π]*.*

Proof See Corollary 2, Section S3 of the Supplementary Material. □

Corollary 2 shows that, depending on the intensity function and hence $\overset{¯}{\rho }=\inf_{x\in S^{2}\rho \left(x\right)}$, the bias can be considered negligible. In the examples to come we set the expected number of events in the process to be large enough for the bias to be considered negligible. Next we provide the variance of the estimators of the functional summary statistics.

Theorem 4 *Let* X *be a spherical Poisson process on* $S^{2}$ *with known intensity function* $\rho :S^{2}\mapsto R_{+}$*, such that* $\overset{¯}{\rho }=\inf_{x\in S^{2}}\rho \left(x\right)>0$*. Then the estimators* ${\overset{ˆ}{K}}_{\mathrm{inhom}}\left(r\right)$*,* ${\overset{ˆ}{F}}_{\mathrm{inhom}}\left(r\right)$*, and* ${\overset{ˆ}{H}}_{\mathrm{inhom}}\left(r\right)$ *have variance,*

Var(Kˆinhom(r))=18π2∫S2∫S21[d(x,y)≤r]ρ(x)ρ(y)λS2(dx)λS2(dy)+(1−cosr)2∫S21ρ(x)λS2(dx),Var(Fˆinhom(r))=exp−2ρ¯λS2(BS2(o,r))|P|2AAAAAAAA∑p∈P∑p′∈Pexp∫BS2(p,r)∩BS2(p′,r)ρ¯2ρ(x)λS2(dx)−exp−2ρ¯λS2(BS2(o,r)),Var(Hˆinhom(r))=1μ2(S2)∫S2∫S2ρ(x)−ρ¯1[x∈BS2(y,r)]ρ(y)−ρ¯1[y∈BS2(x,r)]=+e−μ(S2)A12(x,y)eμ(S2)A1(x,y)−1−Ei(μ(S2)A1(x,y))+γ+log(μ(S2)A1(x,y))λS2(dx)λS2(dy)=+1μ(S2)∫S2e−μ(S2)A2(x)γ+log(μ(S2)A2(x))−Ei(μ(S2)A2(x))ρ(y)λS2(dy)=−e−2μ(S2)1−ρ¯μ(S2)2π(1−cosr)2eμ(S2)1−ρ¯μ(S2)2π(1−cosr)−12

*where,*

$$A_{1}\left(x,y\right)=1-\frac{2\overset{¯}{\rho }}{\mu \left(S^{2}\right)}2\pi \left(1-\cos r\right)+\frac{{\overset{¯}{\rho }}^{2}}{\mu \left(S^{2}\right)}\int_{B_{S^{2}}\left(x,r\right)\cap B_{S^{2}}\left(y,r\right)}\frac{1}{\rho \left(z\right)}\lambda_{S^{2}}\left(dz\right)$$

$$A_{2}\left(x\right)=1-\frac{2\overset{¯}{\rho }}{\mu \left(S^{2}\right)}2\pi \left(1-\cos r\right)+\frac{{\overset{¯}{\rho }}^{2}}{\mu \left(S^{2}\right)}\int_{B_{S^{2}}\left(x,r\right)}\frac{1}{\rho \left(y\right)}\lambda_{S^{2}}\left(dy\right)$$

$$\mathrm{Ei}\left(x\right)=-\int_{-x}^{\infty }\frac{e^{-t}}{t}dt$$

*and* Ei(x) *is the exponential integral and* r∈[0,π]*.*

Proof See Theorem 4, Section S4 of the Supplementary Material. □

Due to the complexity of the estimator for the ${\overset{ˆ}{J}}_{\text{inhom}}$-function, its mean and variance are extremely complex and although can be derived in terms of integrals over $S^{2}$, we instead give an approximation based on the Taylor series expansion of the function f(x,y)=x∕y around the means of the numerator and denominator. We first provide conditions for which the first two moments of ${\overset{ˆ}{J}}_{\text{inhom}}\left(r\right)$ exist and then proceed to show how it can be approximated.

Theorem 5 *Let* X *be a spheroidal Poisson process with intensity function* $\rho :S^{2}\mapsto R_{+}$ *such that* $\overset{¯}{\rho }\equiv \inf_{x\in S^{2}}\rho \left(x\right)>0$*. Let* P *be any finite grid on* $S^{2}$ *and define* $r_{\max}=\sup\left\{r\in \left[0,\pi \right]:\text{ there exists }p\in P\text{ such that }\rho \left(x\right)\neq \overset{¯}{\rho }\text{ for all }x\in B_{S^{2}}\left(p,r\right)\right\}$*. Then for any given* $r\in \left[0,r_{\max}\right]$ *both* $E\left[{\overset{ˆ}{J}}_{\mathrm{inhom}}\left(r\right)\right]$ *and* $\mathrm{Var}\left({\overset{ˆ}{J}}_{\mathrm{inhom}}\left(r\right)\right)$ *exist.*

Proof See Theorem 5, Section S5 of the Supplementary Material. □

Proposition 1 *Let* X *be a spheroidal Poisson process with known intensity function* $\rho :S^{2}\mapsto R_{+}$*. Then the covariance between* $1-{\overset{ˆ}{H}}_{\text{inhom}}\left(r\right)$ *and* $1-{\overset{ˆ}{F}}_{\text{inhom}}\left(r\right)$ *for* r∈[0,π] *is,*

$$\text{Cov}\left(1-{\overset{ˆ}{H}}_{\text{inhom}}\left(r\right),1-{\overset{ˆ}{F}}_{\text{inhom}}\left(r\right)\right)=\frac{1}{\left|P\right|}\underset{p\in P}{\sum }\int_{S^{2}}\left(1-\frac{\overset{¯}{\rho }1\left[x\in B_{S^{2}}\left(p,r\right)\right]}{\rho \left(x\right)}\right)$$

=−exp−2ρ¯2π(1−cosr)−∫BS2(x,r)∩BS2(p,r)ρ¯2ρ(y)λS2(dy)A(x,p)ρ(x)μ(S2)λS2(dx)

=−exp(−2π(1−cosr)ρ¯)(exp(−2π(1−cosr)ρ¯)−exp(−μ(S2))μ(S2)μ(S2)−2π(1−cosr)ρ¯,

*where* P *is a finite grid of points on* $S^{2}$ *and,*

$$A\left(x,p\right)=1-\frac{2\overset{¯}{\rho }}{\mu \left(S^{2}\right)}2\pi \left(1-\cos r\right)+\frac{1}{\mu \left(S^{2}\right)}\int_{B_{S^{2}}\left(x,r\right)\cap B_{S^{2}}\left(p,r\right)}\frac{{\overset{¯}{\rho }}^{2}}{\rho \left(y\right)}\lambda_{S^{2}}\left(dy\right).$$

Proof See Proposition 1, Section S5 of the Supplementary Material. □

Using a Taylor series expansion (see Section S5.2 of the Supplementary Material), we can approximate the expectation and variance of ${\overset{ˆ}{J}}_{\text{inhom}}\left(r\right)$ as

(16) $$E\left[\frac{X}{Y}\right]\approx \frac{\mu_{X}}{\mu_{Y}}-\frac{\text{Cov}\left(X,Y\right)}{\mu_{Y}^{2}}+\frac{\text{Var}\left(Y\right)\mu_{X}}{\mu_{Y}^{3}}$$

(17) $$\text{Var}\left(\frac{X}{Y}\right)\approx \frac{\mu_{X}}{\mu_{Y}}\left[\frac{\text{Var}\left(X\right)}{\mu_{X}^{2}}-2\frac{\text{Cov}\left(X,Y\right)}{\mu_{X}\mu_{Y}}+\frac{\text{Var}\left(Y\right)}{\mu_{Y}^{2}}\right],$$

where $X=1-{\overset{ˆ}{H}}_{\text{inhom}}\left(r\right)$ and $Y=1-{\overset{ˆ}{F}}_{\text{inhom}}\left(r\right)$. The terms in Eqs. (16), (17) are given in Theorem 3, Theorem 4, and Proposition 1.

## 6 Examples

We now look at two examples where we simulate homogeneous Poisson processes on their surfaces and construct the previously described functional summary statistics.

**Fig. 1:**
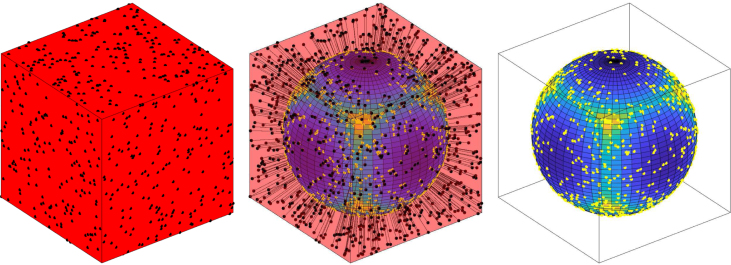
Example of simulating and mapping a CSR process on the cube to the sphere. *Left:* example of a CSR process on a cube with l=1 and constant intensity function 50. *Middle:* mapping of events from cube to the sphere by the function f(x)=x∕‖x‖. *Right:* mapped point pattern on the sphere with the new intensity function indicated by the colour on the sphere. High intensity is indicated by light areas whilst low intensity is indicated by dark areas.

### 6.1 Cube

We define a centred cube over each of the six faces with a side length 2l, where l=1. The level-set function for a cube is,

$$g\left(x\right)=\left\{\begin{array}{c} x_{3}-l,\;\text{for }-l\leq x_{1},x_{2}\leq l\;\\ x_{3}+l,\;\text{for }-l\leq x_{1},x_{2}\leq l\;\\ x_{2}-l,\;\text{for }-l\leq x_{1},x_{3}\leq l\;\\ x_{2}+l,\;\text{for }-l\leq x_{1},x_{3}\leq l\;\\ x_{1}-l,\;\text{for }-l\leq x_{2},x_{3}\leq l\;\\ x_{1}+l,\;\text{for }-l\leq x_{2},x_{3}\leq l.\; \end{array}\right)$$

Using Theorem 2 we can derive the intensity function for the point process that is mapped to the sphere. By symmetry we need only consider one of the faces of the cube and by rotation we will be able to derive the intensity function on the sphere. Consider the bottom face, i.e. $x_{3}=-l$, and in the notation of Theorem 2 label this $D_{1}$. Then,

$$l_{1}\left(x\right)=1$$

$$J_{\left(1,f^{*}\right)}\left(x\right)={\left(1+x_{1}+x_{2}\right)}^{2},$$

and so the intensity function over $f\left(D_{1}\right)$ is,

$$\rho_{1}^{*}\left(x\right)=\rho {\left(1+{\left(f_{1}^{*-1}\left(x_{1}\right)\right)}^{2}+{\left(f_{2}^{*-1}\left(x_{2}\right)\right)}^{2}\right)}^{2}{\left(1-x_{1}^{2}-x_{2}^{2}\right)}^{\frac{1}{2}},$$

where $f_{i}\left(x_{i}\right)=x_{i}∕||x||$. Thus by the appropriate rotations the intensity function over the entire sphere is,

$$\rho^{*}\left(x\right)=\left\{\begin{array}{c} \rho {\left(1+{\left(f_{1}^{*-1}\left(x_{1}\right)\right)}^{2}+{\left(f_{2}^{*-1}\left(x_{2}\right)\right)}^{2}\right)}^{2}{\left(1-x_{1}^{2}-x_{2}^{2}\right)}^{\frac{1}{2}},\;x\in f\left(D_{1}\right)\cup f\left(D_{2}\right)\;\\ \rho {\left(1+{\left(f_{1}^{*-1}\left(x_{1}\right)\right)}^{2}+{\left(f_{3}^{*-1}\left(x_{3}\right)\right)}^{2}\right)}^{2}{\left(1-x_{1}^{2}-x_{3}^{2}\right)}^{\frac{1}{2}},\;x\in f\left(D_{3}\right)\cup f\left(D_{4}\right)\;\\ \rho {\left(1+{\left(f_{2}^{*-1}\left(x_{2}\right)\right)}^{2}+{\left(f_{3}^{*-1}\left(x_{3}\right)\right)}^{2}\right)}^{2}{\left(1-x_{2}^{2}-x_{3}^{2}\right)}^{\frac{1}{2}},\;x\in f\left(D_{5}\right)\cup f\left(D_{6}\right),\; \end{array}\right)$$

where $D_{1},D_{2},D_{3},D_{4},D_{5}$, and $D_{6}$ are the faces such that z=−1,z=1,y=−1,y=1,x=−1, and x=1 respectively. Fig. 1 demonstrates mapping from a cube with l=1 and ρ=50 to the unit sphere where the shading over the sphere indicates areas of low (dark) and high (light) intensity. The figure also shows an example of a CSR pattern over the cube and how this pattern changes under the mapping.

**Fig. 2:**
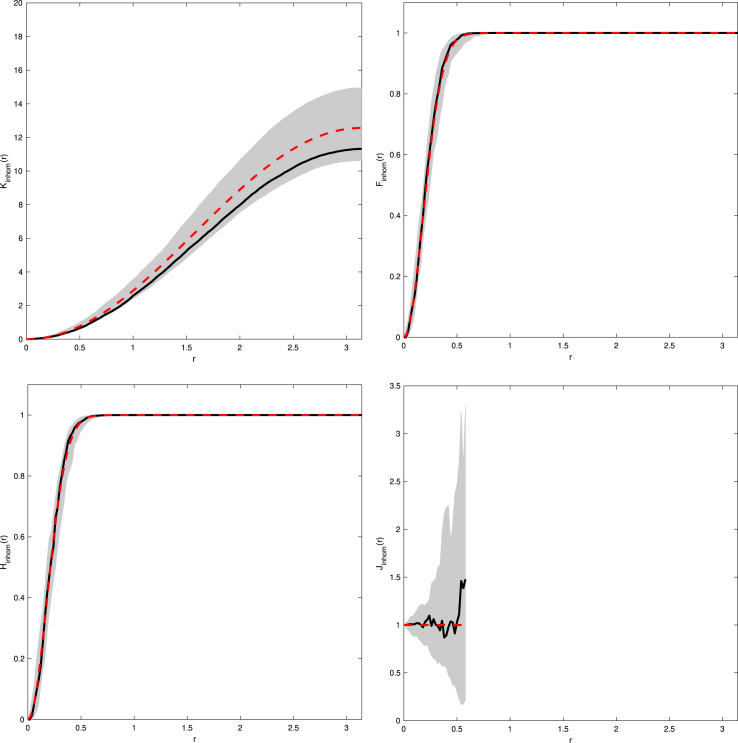
Examples of $K_{\text{inhom}}$- (*top left*), $F_{\text{inhom}}$- (*top right*), $H_{\text{inhom}}$- (*bottom left*), and $J_{\text{inhom}}$- (*bottom right*) functions for CSR patterns on a cube with l=1 and ρ=5. Solid line is the estimated functional summary statistic for our observed data, dashed line is the theoretical functional summary statistic for a Poisson process, and the grey shaded area represents the simulation envelope from 99 Monte Carlo simulations of Poisson processes fitted to the observed data.

In order to be able to construct the inhomogeneous F-, and H-function we need to determine $\inf_{x\in S^{2}}\rho^{*}\left(x\right)$. By the nature of the function f(x)=x∕‖x‖ and assuming that l≥1, then mapping events from the cube to the sphere causes events to be more concentrated on the sphere compared to the cube, thus increasing the corresponding intensity on the sphere. Therefore, the lowest achievable intensity occurs at the centre of each face of the cube, i.e. for the bottom face it occurs when $x_{1}=x_{2}=0$, giving $\inf_{x\in S^{2}}\rho^{*}\left(x\right)=\rho$. Fig. 2 gives examples of the inhomogeneous K-, F-, H-, and J-functions where l=1 and ρ=5 and are typical when the observed process is CSR.

### 6.2 Ellipsoid

An ellipsoid is defined by its semi-major axis lengths a,b,c∈R along x-, y-, and z-axis respectively. Again we also assume that the ellipsoid is centred at the origin. The level-set function for ellipsoids is given by $g\left(x\right)=x_{1}^{2}∕a^{2}+x_{2}^{2}∕b^{2}+x_{3}^{2}∕c^{2}-1$, in this current form $\overset{\sim }{g}$ is not well defined in which case we shall use the following equivalent representation

$$g\left(x\right)=\left\{\begin{array}{c} x_{1}^{2}∕a^{2}+x_{2}^{2}∕b^{2}+x_{3}^{2}∕c^{2}-1,\;\text{for }x_{3}\geq 0\;\\ x_{1}^{2}∕a^{2}+x_{2}^{2}∕b^{2}+x_{3}^{2}∕c^{2}-1,\;\text{for }x_{3}<0.\; \end{array}\right)$$

This representation then allows for $\overset{\sim }{g}$ to be well defined for each partition of the ellipsoid.

**Fig. 3:**
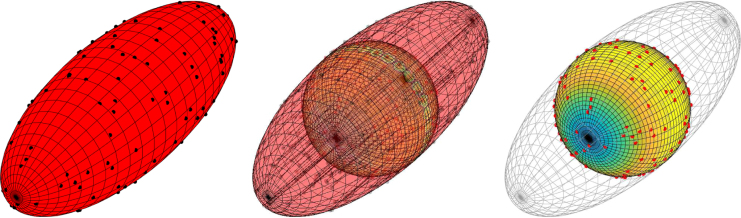
Example of simulating and mapping a CSR process on a prolate ellipsoid to the sphere. *Left:* example of a CSR process on a prolate ellipsoid with a=b=1,c=3 and ρ=5. *Middle:* mapping of events from prolate ellipsoid to the sphere by the function $f\left(x\right)={\left(x_{1}∕a,x_{2}∕b,x_{3}∕c\right)}^{T}$. *Right:* mapped point pattern on the sphere with the new intensity function indicated by the colour on the sphere. High intensity is indicated by light areas whilst low intensity is indicated by dark areas.

We now demonstrate our methodology on an ellipsoid with semi-major axis lengths a=1,b=1, and c=3 along the x-, y-, and z-axis respectively. Instead of using the function f(x)=x∕‖x‖ to map from the ellipsoid to the sphere, we can use a simpler mapping function which makes calculation of the determinant $J_{\left(i,f^{*}\right)}\left(x\right)$ in Theorem 2 significantly easier. We can simply scale along the axis directions, i.e. use the mapping $f\left(x\right)={\left(x_{1}∕a,x_{2}∕b,x_{3}∕c\right)}^{T}$. Using this mapping function, as opposed to dividing each vector by the norm of itself, and focusing on the bottom hemiellipsoid (indicated by the minus superscript), then

$$l_{-}\left(x\right)=\sqrt{\frac{1-\left(1-\frac{c^{2}}{a^{2}}\right)x_{1}^{2}-\left(1-\frac{c^{2}}{b^{2}}\right)x_{2}^{2}}{1-x_{1}^{2}-x_{2}^{2}}},\;J_{\left(-,f^{*}\right)}\left(x\right)=ab,$$

and so on the lower hemisphere the intensity function takes the form

$$\rho_{-}^{*}\left(x\right)=\rho ab\sqrt{1-\left(1-\frac{c^{2}}{a^{2}}\right)x_{1}^{2}-\left(1-\frac{c^{2}}{b^{2}}\right)x_{2}^{2}}.$$

By symmetry the mapped intensity function over the whole sphere is then

$$\rho^{*}\left(x\right)=\rho ab\sqrt{1-\left(1-\frac{c^{2}}{a^{2}}\right)x_{1}^{2}-\left(1-\frac{c^{2}}{b^{2}}\right)x_{2}^{2}}.$$

**Fig. 4:**
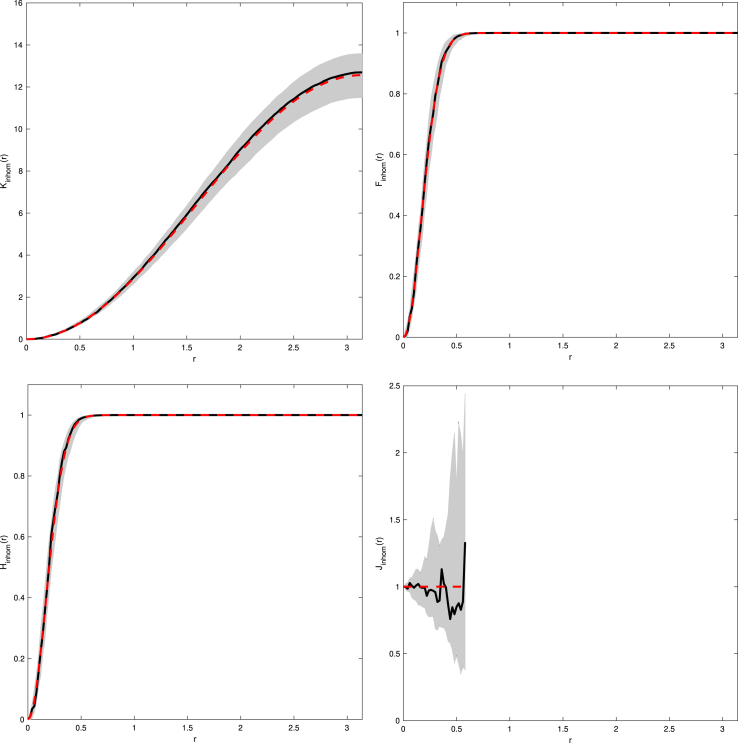
Examples of $K_{\text{inhom}}$- (*top left*), $F_{\text{inhom}}$- (*top right*), $H_{\text{inhom}}$- (*bottom left*), and $J_{\text{inhom}}$- (*bottom right*) functions for CSR patterns on a prolate ellipsoid with a=b=1,c=3, and ρ=5. Solid line is the estimated functional summary statistic for our observed data, dashed line is the theoretical functional summary statistic for a Poisson process, and the grey shaded area represents the simulation envelope from 99 Monte Carlo simulations of Poisson processes fitted to the observed data.

Again we need to calculate $\inf_{x\in S^{2}}\rho^{*}\left(x\right)$. Noting that c≥a=b, thus $-\left(1-c^{2}∕a^{2}\right)\geq 0$ and $-\left(1-c^{2}∕b^{2}\right)\geq 0$, then the square root term is minimised when $x_{1}$ and $x_{2}$ are 0, hence $\inf_{x\in S^{2}}\rho^{*}\left(x\right)=\rho ab$. An example of this mapping is given in Fig. 3. Using this we can construct the estimators of the inhomogeneous functional summary statistics given by Eqs. (12)–(15). Examples are given in Fig. 4. These figures are typical for CSR with the estimated functional summary statistics lying well within the simulation envelopes.

## 7 Regular & cluster processes on D

We examine some regular and cluster processes on D. In particular, we examine how functional summary statistics constructed under the Poisson hypothesis deviate when the underlying process is in fact not Poisson. We shall be using the Matérn I and II inhibition processes (Chiu et al., 2013) as examples of regular processes, and Thomas processes as a cluster example. Definitions for the Matérn I, II and Thomas processes on convex shapes will also be presented, whilst properties of such processes are given in Section S6 of the Supplementary Material.

### 7.1 Examples of regular and cluster processes on convex shapes

A common way of defining a regular process is using a minimum distance R, known as the hardcore distance, for which no events in the process has a nearest neighbour closer than R. In typical applications R is usually the Euclidean distance (in $R^{d}$) or the great circle distance (in $S^{2}$), but on an arbitrary three dimensional convex shape, D, this distance is taken as the geodesic distance defined by the surface. The following definitions extend the Matérn I and II processes to a convex shape with geodesic distance d(x,y), x,y∈D.

Definition 3 Let X be a homogeneous Poisson process on D with intensity $\rho \in R_{+}$. Fix R>0, and thin X according to the following rule: delete events x∈X if there exists y∈X∖{x} such that d(x,y)<R, otherwise retain x. The resulting thinned process is then defined as a Matérn I inhibition process on D.

Definition 4 Let X be a homogeneous Poisson process on D with intensity $\rho \in R_{+}$. Fix R>0, and let each x∈X have an associated mark, $M_{x}$ drawn from some mark density $P_{M}$ independently of all other marks and events in X. Thin X according to the following rule: delete the event x∈X if there exists y∈X∖{x} such that d(x,y)<R and $M_{y}<M_{x}$, otherwise retain x. The resulting thinned process is then defined as a Matérn II inhibition process on D.

We also extend the Neyman–Scott process, a class of cluster processes, to arbitrary convex shapes.

Definition 5 Let $X_{P}$ be a homogeneous Poisson process on D with intensity $\rho \in R_{+}$. Then for each $c\in X_{P}$ define $X_{c}$ to the point process with intensity function $\rho_{c}\left(x\right)=\alpha k\left(x,c\right)$, where α>0 and k:D×D↦R is a density function and $N_{X_{c}}\left(D\right)$ can be any random counting measure associated to $X_{c}$. The point process $X=\cup_{c\in X_{p}}X_{c}$ is a Neyman–Scott process.

A Thomas process is a specific Neyman–Scott process where the density function k(⋅,⋅) has a specific form. In $R^{2}$, k is taken to be an isotropic bivariate Gaussian distribution (Møller and Waagepetersen, 2004), whilst on $S^{2}$ it is taken as the Von-Mises Fisher distribution (Lawrence et al., 2016). We define a Thomas process on D to be a Neyman–Scott process with density function k of the form,

$$k\left(x,y\right)=\frac{1}{\chi \left(\kappa^{2}\right)}\exp\left(-\frac{d^{2}\left(x,y\right)}{2\kappa^{2}}\right),$$

where κ is a bandwidth parameter and $\chi \left(\kappa \right)=\int_{D}\exp\left(-d\left(x,y\right)∕2\kappa^{2}\right)\lambda_{D}\left(y\right)$. This is known as the Riemannian Gaussian distribution (Said et al., 2017), where on the plane this would reduce to an isotropic bivariate Gaussian.

### 7.2 Functional summary statistics assuming a homogeneous Poisson process

**Fig. 5:**
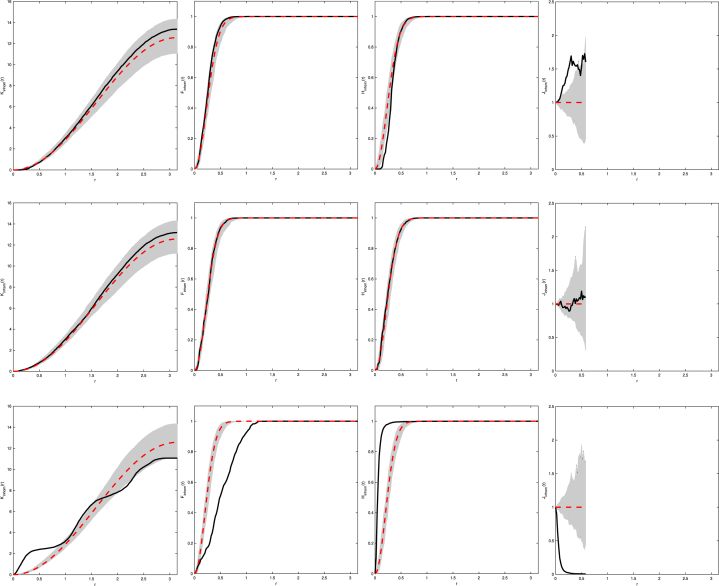
Example of (from left to right) $K_{\text{inhom}}$-, $J_{\text{inhom}}$-, $F_{\text{inhom}}$-, and $H_{\text{inhom}}$-functions for a Matérn II with parameters R=0.3 and exponential mark distribution with rate λ=1 (*top row*), Poisson process (*middle row*), and Thomas process with parameters κ=0.1 and offspring expectation 15 (*bottom row*) on a prolate spheroid with dimensions (a,b,c)=(1,1,3) all with expectation 100. Solid line is the estimated functional summary statistics for our observed data, dashed line is the theoretical functional summary statistic for a Poisson process, and the grey shaded area is the simulation envelopes from 99 Monte Carlo simulations of Poisson processes fitted to the observed data.

We simulate Matérn II, and Thomas processes and construct estimates of their functional summary statistics under the assumption that they are CSR. The inhomogeneous functional summary statistics are displayed in Fig. 5. Comparing Fig. 5 to typical functional summary statistics for regular and cluster processes in $R^{2}$, we see the same types of deviations away from CSR. In particular, we see for regular processes with small r that there are negative deviations, whilst the cluster process has large positive deviations for the ${\overset{\sim }{K}}_{\text{inhom}}$-function. Furthermore, the ${\overset{ˆ}{J}}_{\text{inhom}}$-function shows significant positive deviations for regular processes whilst negative ones are observed for cluster processes.

Fig. 5 highlights the importance of considering many different functional summary statistics when attempting to draw conclusions from the data (Diggle, 2003). More precisely consider the top row of Fig. 5 which refers to a Matérn II process with hardcore distance R=0.3 and $E\left[N_{X}\left(D\right)\right]=100$. If we were to only consult the $K_{\text{inhom}}$ simulation envelope (top left figure) then we may be hesitant to reject CSR, but when we examine the $H_{\text{inhom}}$ and $J_{\text{inhom}}$ simulation envelope plots there is strong evidence to suggest that this process is not CSR and is in fact regular. Thus, for this specific setting, the simulation envelope plots of the $J_{\text{inhom}}$ function provide greater power compared to those produced with the $K_{\text{inhom}}$ function, especially for smaller r (Baddeley et al., 2015 p. 235). This effect is also seen in Figure 1 of van Lieshout (2011) for Log Gaussian Cox processes on $R^{2}$, where for small values of r the $K_{\text{inhom}}$ is unable to provide evidence against the process being inhomogeneous Poisson but examining both the $F_{\text{inhom}}$ and $H_{\text{inhom}}$ provides greater evidence against this hypothesis.

This phenomena was first discussed by Baddeley and Silverman (1984), where they show that it is possible to construct planar processes which are not homogeneous Poisson but have the same K-function. This is also discussed by Baddeley et al. (2000) in Section 2.4 in the inhomogeneous setting. Similar arguments to those in Baddeley et al. (2000) and Baddeley and Silverman (1984) can be used to construct spheroidal processes that are not Poisson but exhibit the same K-function as any given Poisson process with the same intensity. This discussion serves as a precautionary warning to consulting only one individual functional summary statistic and it is therefore important to consider many to avoid drawing improper conclusions from data.

## 8 Testing for CSR on convex shapes in $R^{3}$

Exploratory data analysis for spatial point patterns in $R^{2}$ typically begins with testing whether the observed point pattern exhibits CSR where test statistics are frequently based on the L-function, $L\left(r\right)=\sqrt{K\left(r\right)∕\pi }$. On $R^{2}$ and under CSR the L-function is linear in r and variance stabilised (Besag, 1977) whilst Lawrence (2018) discusses the analogue L-function in $S^{2}$ where again it is variance stabilised when the underlying process is CSR. As we are working with inhomogeneous Poisson processes on $S^{2}$ an equivalent transformation for the L function has not been discussed previously. We propose two test statistics:

1. an extension of the analogue L - function proposed by Lawrence (2018) to the inhomogeneous setting, and
2. a standardisation of the inhomogeneous K - function inspired by Lagache et al. (2013).

In order to construct the latter we must derive first and second order properties of the estimated functional summary statistics. Section 7 discusses derivations for any spherical Poisson process when ρ is known. In this section we consider the scenario when we have a homogeneous Poisson process on D with *unknown* intensity $\rho \in R_{+}$. Furthermore we shall only focus on the inhomogeneous K-function as standardisation of the remaining functional summary statistics follow identically.

### 8.1 Test statistic for CSR

Given a homogeneous Poisson process on D with intensity $\rho \in R_{+}$, we map this to $S^{2}$ giving a new Poisson process on the sphere with inhomogeneous intensity function given by Theorem 2 as

(18) $$\rho^{*}\left(x\right)=\left\{\begin{array}{cc} \rho l_{1}\left(f^{-1}\left(x\right)\right) & J_{\left(1,f^{*}\right)}\left(x\right)\sqrt{1-x_{1}^{2}-x_{2}^{2}},\;x\in f\left(D_{1}\right)\\ & \vdots \\ \rho l_{n}\left(f^{-1}\left(x\right)\right) & J_{\left(n,f^{*}\right)}\left(x\right)\sqrt{1-x_{1}^{2}-x_{2}^{2}},\;x\in f\left(D_{n}\right). \end{array}\right)$$

Using Theorem 3, Theorem 4 we can calculate the mean and variances of the inhomogeneous K-function when ρ is known. When ρ is unknown we use estimators of ρ when constructing functional summary statistics. In particular we use

$$\overset{ˆ}{\rho }=\frac{N_{X}\left(D\right)}{\lambda_{D}\left(D\right)},\;\overset{ˆ}{\rho^{2}}=\frac{N_{X}\left(D\right)\left(N_{X}\left(D\right)-1\right)}{\lambda_{D}^{2}\left(D\right)},$$

which are both unbiased for ρ and $\rho^{2}$ respectively by application of the Campbell–Mecke Theorem (Møller and Waagepetersen, 2004). Thus our estimator for $K_{\text{inhom}}\left(r\right)$ when ρ is unknown takes the following form,

(19) $${\overset{\sim }{K}}_{\text{inhom}}\left(r\right)=\left\{\begin{array}{cc} \frac{\lambda_{D}^{2}\left(D\right)}{4\pi N_{Y}\left(S^{2}\right)\left(N_{Y}\left(S^{2}\right)-1\right)}\underset{x\in Y}{\sum }\underset{y\in Y\setminus \left\{x\right\}}{\sum }\frac{1\left[d\left(x,y\right)\leq r\right]}{\overset{\sim }{\rho }\left(x\right)\overset{\sim }{\rho }\left(y\right)},\; & \text{if }N_{Y}\left(S^{2}\right)>1\\ 0,\; & \text{otherwise}, \end{array}\right)$$

where Y=f(X), f is our mapping from the ellipsoid to the sphere, and $\overset{\sim }{\rho }\left(x\right)$ is given by,

(20) $$\overset{\sim }{\rho }\left(x\right)=\left\{\begin{array}{cc} l_{1}\left(f^{-1}\left(x\right)\right) & J_{\left(1,f^{*}\right)}\left(x\right)\sqrt{1-x_{1}^{2}-x_{2}^{2}},\;x\in f\left(D_{1}\right)\\ & \vdots \\ l_{n}\left(f^{-1}\left(x\right)\right) & J_{\left(n,f^{*}\right)}\left(x\right)\sqrt{1-x_{1}^{2}-x_{2}^{2}},\;x\in f\left(D_{n}\right). \end{array}\right)$$

Note that $N_{Y}\left(S^{2}\right)=N_{X}\left(D^{2}\right)$.

An analogue L - function for isotropic spherical point processes was introduced by Lawrence (2018) in which the square root of Ripley’s spherical K - function is taken. This transformation benefits from approximate variance stabilisation in the same sense as the L - function does in $R^{d}$ (Besag, 1977) but is not linearised. In the planar setting a multiplicative factor of $1∕\sqrt{\pi }$ can be used such that L(r)=r but due to the more complex form of K on $S^{2}$ a simple linearising transformation is not intuitive. Therefore Lawrence (2018) suggests subtracting the theoretical value in order for the summary statistic to be zero in the event a process is Poisson. Following this line of thought we then propose, in the inhomogeneous setting, the following functional summary statistic $P_{\text{inhom}}\left(r\right)=\sqrt{K_{\text{inhom}}\left(r\right)}-\sqrt{2\pi \left(1-\cos\left(r\right)\right)}$, where we use $P_{\text{inhom}}$ rather than $L_{\text{inhom}}$ to avoid confusion with the Euclidean L - function as $P_{\text{inhom}}$ takes a very different form to its Euclidean counterpart. In the event of a homogeneous Poisson process over D (and hence a inhomogeneous Poisson process over $S^{2}$) we can estimate $P_{\text{inhom}}$ as,

$${\overset{\sim }{P}}_{\text{inhom}}\left(r\right)=\sqrt{{\overset{\sim }{K}}_{\text{inhom}}\left(r\right)}-\sqrt{2\pi \left(1-\cos\left(r\right)\right)}.$$

Diggle (2003) proposes using the maximum absolute value between the theoretical and the estimated functional summary statistics to test for CSR. Based on this we propose the following two test statistics,

(21) T1=supr∈[0,π]|P~inhom(r)|,T2=supr∈[0,π]|K~inhom(r)−2π(1−cos(r))Var^(K~inhom(r))|,

the first based on the work of Lawrence (2018) and the second on the work of Lagache et al. (2013). In order to be able to construct the test statistic $T_{2}$, an estimate of the variance of the empirical functional summary statistics are required. Further, we need show that the bias of ${\overset{\sim }{K}}_{\text{inhom}}\left(r\right)$ is negligible and hence $E\left[{\overset{\sim }{K}}_{\text{inhom}}\left(r\right)\right]\approx 2\pi \left(1-\cos\left(r\right)\right)$ for Poisson processes, validating its use in (21). By using estimators for ρ and $\rho^{2}$ we alter the first and second order properties given by Theorem 3, Theorem 4. In the following we consider the first and second order moments of ${\overset{\sim }{K}}_{\text{inhom}}$.

### 8.2 Estimating moments of ${\overset{\sim }{K}}_{\text{inhom}}\left(r\right)$ on $S^{2}$ for CSR process on D

Theorem 6 *The bias and variance of* ${\overset{\sim }{K}}_{\text{inhom}}\left(r\right)$ *are,*

$$\text{Bias}\left({\overset{\sim }{K}}_{\text{inhom}}\left(r\right)\right)=-P\left(N_{Y}\left(S^{2}\right)\leq 1\right)2\pi \left(1-\cos r\right),$$

*and,*

(22) $$\text{Var}\left({\overset{\sim }{K}}_{\text{inhom}}\left(r\right)\right)=4\pi^{2}{\left(1-\cos r\right)}^{2}\left(1-P\left(N_{Y}\left(S^{2}\right)\leq 1\right)\right)P\left(N_{Y}\left(S^{2}\right)\leq 1\right)$$

AAAA+ρ3λD4(D)(1−cosr)2∫S21ρ~(x)λS2(dx)−16π2λD(D)E1(NY(S2)+3)2(NY(S2)+2)2

AAAA+ρ2λD4(D)8π2∫S2∫S21[d(x1,x2)≤r]ρ~(x1)ρ~(x2)λS2(dx1)λS2(dx2)−64π4(1−cosr)2λD2(D)

AAAA×E1(NY(S2)+2)2(NY(S2)+1)2,

*where* $\overset{\sim }{\rho }\left(x\right)$ *is given by* Eq. (20)*.*

Proof See Theorem 6, Section S7 of the Supplementary Material. □

The form of the variance derived in Theorem 6 is near identical to that derived by Lang and Marcon (2013) except that our derivations consider inhomogeneous Poisson processes, does not require corrections for edge effects, and the space is $S^{2}$ instead of $R^{2}$. Further we can bound the absolute value of the bias as follows

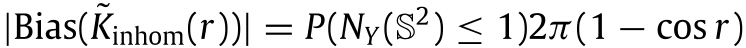

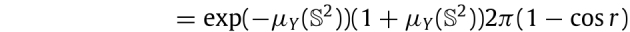

(23)
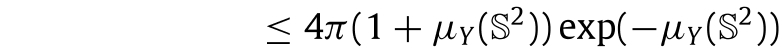

(24)
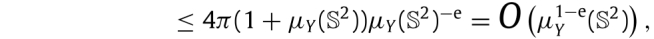

where $\mu_{Y}$ is the intensity measure of Y=f(X), the inequality in (23) is attained by setting r=π, and (24) follows from $e^{x}\geq x^{e}$. Thus, for shapes considered in this work, the bias will be negligible.

From Theorem 6 it is possible to construct a ratio-unbiased estimator for the variance. In particular by the Campbell–Mecke Theorem, and defining the estimator ${\overset{ˆ}{\rho }}_{k}=N_{Y}\left(S^{2}\right)\left(N_{Y}\left(S^{2}\right)-1\right)\cdots \left(N_{Y}\left(S^{2}\right)-k-1\right)∕\lambda_{D}^{k}\left(D\right)$, then $E\left[{\overset{ˆ}{\rho }}_{k}\right]=\rho^{k}$ and so ${\overset{ˆ}{\rho }}_{k}$ is unbiased for $\rho^{k}$. We can substitute the expectations in (22) with their corresponding observed values, for example we substitute ${\left(N_{Y}\left(S^{2}\right)+3\right)}^{-2}{\left(N_{Y}\left(S^{2}\right)+2\right)}^{-2}$ for $E\left[{\left(N_{Y}\left(S^{2}\right)+3\right)}^{-2}{\left(N_{Y}\left(S^{2}\right)+2\right)}^{-2}\right]$. Additionally, the following lemma helps derive a ratio unbiased estimator for $P\left(N_{Y}\left(S^{2}\right)<1\right)$.

Lemma 2 *Let* N∼Poisson(λ)*,* k∈N *and* $p\in R_{+}$*. Define the following random variable,*

(25) $$R=\frac{N!e^{N-k}}{\left(N-k\right)!{\left(e+p\right)}^{N}}.$$

*Then* R *is ratio-unbiased for* $\lambda^{k}e^{-p\lambda }$*.*

Proof See Lemma 2, Section S7 of the Supplementary Material. □

Using Lemma 2 we can construct a ratio-unbiased estimator for $\left(1-P\left(N_{Y}\left(S^{2}\right)<1\right)\right)P\left(N_{Y}\left(S^{2}\right)<1\right)$. Defining $\lambda =\rho \lambda_{D}\left(L\right)$,

$$\left(1-P\left(N_{Y}\left(S^{2}\right)<1\right)\right)P\left(N_{Y}\left(S^{2}\right)<1\right)=\left(1-e^{-\lambda }-\lambda e^{-\lambda }\right)\left(e^{-\lambda }+\lambda e^{-\lambda }\right)=e^{-\lambda }+\lambda e^{-\lambda }-e^{-2\lambda }-2\lambda e^{-2\lambda }-\lambda^{2}e^{-2\lambda },$$

and so a ratio-unbiased estimator for $\left(1-P\left(N_{Y}\left(S^{2}\right)<1\right)\right)P\left(N_{Y}\left(S^{2}\right)<1\right)$ is

$$\frac{e^{N_{Y}\left(S^{2}\right)}}{{\left(e+1\right)}^{N_{Y}\left(S^{2}\right)}}+\frac{N_{Y}\left(S^{2}\right)e^{N_{Y}\left(S^{2}\right)-1}}{{\left(e+1\right)}^{N_{Y}\left(S^{2}\right)}}+\frac{e^{N_{Y}\left(S^{2}\right)}}{{\left(e+2\right)}^{N_{Y}\left(S^{2}\right)}}-\frac{2N_{Y}\left(S^{2}\right)e^{N_{Y}\left(S^{2}\right)-1}}{{\left(e+2\right)}^{N_{Y}\left(S^{2}\right)}}$$

$$\;-\frac{N_{Y}\left(S^{2}\right)\left(N_{Y}\left(S^{2}\right)-1\right)e^{N_{Y}\left(S^{2}\right)-2}}{{\left(e+2\right)}^{N_{Y}\left(S^{2}\right)}}.$$

Plugging the given estimators for $\left(1-P\left(N_{Y}\left(S^{2}\right)<1\right)\right)P\left(N_{Y}\left(S^{2}\right)<1\right)$, $\rho^{2}$, $\rho^{3}$, $E\left[{\left(N_{Y}\left(S^{2}\right)+3\right)}^{-2}\right)\left({\left(N_{Y}\left(S^{2}\right)+2\right)}^{-2}\right]$ and $E\left[{\left(N_{Y}\left(S^{2}\right)+2\right)}^{-2}{\left(N_{Y}\left(S^{2}\right)+1\right)}^{-2}\right]$ into (22) gives a ratio unbiased estimator for $\text{Var}\left({\overset{\sim }{K}}_{\text{inhom}}\left(r\right)\right)$, which in turn allows for the construction of the test statistic $T_{2}$ in (21).

### 8.3 Standardised inhomogeneous K-function plots

**Fig. 6:**
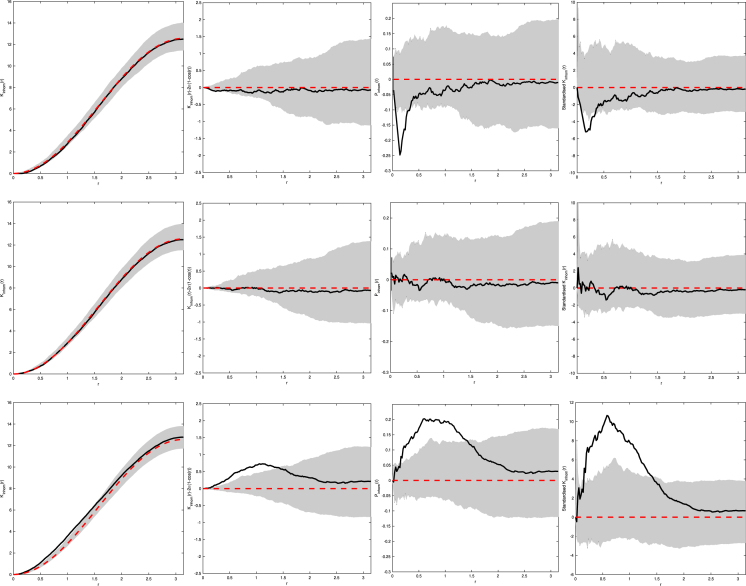
Example of (from left to right) $K_{\text{inhom}}$-, $K_{\text{inhom}}-2\pi \left(1-\cos\left(r\right)\right)$-, $P_{\text{inhom}}$-, and the standardised $K_{\text{inhom}}$-functions for a Matérn II with R=0.2, an exponential mark distribution with rate λ=1 and expectation 100 (*top row*), Poisson process with expectation 40π (*middle row*), and Thomas process with parameters κ=0.5, expectation of 150 and offspring mean of 20 (*bottom row*) on a prolate spheroid with a=b=0.8000,c=1.43983 (dimensions chosen so that the area of the ellipsoid is 4π). Solid line is the estimated functional summary statistics for our observed data, dashed line is the theoretical functional summary statistic for a Poisson process, and the grey shaded area is the simulation envelopes from 999 Monte Carlo simulations of Poisson processes fitted to the observed data.

Fig. 6 highlights how the empirical K-function estimates deviate when the underlying process is not CSR. For the regular processes we notice considerable negative deviations for small r whilst for cluster processes positive deviations are observed, highlighted in the right column of Fig. 6.

Intuitively, this is to be expected, with a near identical reasoning to what is observed for the K-function in $R^{2,3}$. Since the regular process has a hard-core distance between events, we observe estimates for $K_{\text{inhom}}\left(r\right)$ that are close to zero for small r, thus resulting in the large negative deviation observed in Fig. 6. On the other hand, for the Thomas cluster process, we observe events in closer proximity than would be expected for a CSR process, thus the estimated $K_{\text{inhom}}\left(r\right)$ function has large positive deviations away from CSR.

Further to this, the second row of Fig. 6 corresponding to a Poisson process highlights the importance of applying a variance stabilising transform. If we based a test statistic on $K_{\text{inhom}}\left(r\right)-2\pi \left(1-\cos\left(r\right)\right)$ (or even $K_{\text{inhom}}\left(r\right)$) then these plots suggest worst power compared to the $P_{\text{inhom}}$- and the standardised $K_{\text{inhom}}$-functions.

## 9 Simulation study

We conduct empirical Type I and II error studies to evaluate the effectiveness of the proposed test statistic in determining whether or not a point process on a convex shape exhibits CSR. We consider different prolate ellipsoids such that the area of the ellipsoid is the same across differing semi-major axis lengths. This will allow us to determine how the power of our test changes as the space under consideration deforms further away from the unit sphere.

### 9.1 Design of simulations

In order to best understand the properties of our testing procedure we will consider CSR, Matérn II and Thomas processes on different prolate spheroids. We design the experiments such that the expected number of events is similar across all experiments. For both the CSR and Thomas process simulations this is easily controlled. For a Poisson process, the expected number on D is $\rho \lambda_{D}\left(D\right)$ whilst for a Thomas process it is given by Proposition S3, Section S6 in the Supplementary Materials.

On the other hand, the Matérn II process requires a little more attention since Corollary S4 (Section S6 of the Supplementary Materials) limits the maximum expected number of possible events for a given space D. Thus, for a given expected number μ that is less than or equal to the one prescribed by Corollary S4 (Section 6 of the Supplementary Materials) we fix the hard-core distance R and solve the following equation for ρ

(26) $$\int_{B}\frac{1-e^{-\rho \lambda_{D}\left(B_{D}\left(x,R\right)\right)}}{\lambda_{D}\left(B_{D}\left(x,R\right)\right)}\lambda_{D}\left(dx\right)=\mu .$$

A full outline of all the experiments and the parameters chosen are given in Tables 1, 2, and 3 for CSR, regular, and cluster process simulations respectively. Note that when R=0 for the Matérn II process and when κ=∞ for the Thomas process both processes are CSR.

### 9.2 Test statistics

Calculating $\left|{\overset{\sim }{P}}_{\text{inhom}}\left(r\right)\right|$ and |(K~inhom(r)−2π(1−cosr))∕(Var^(K~inhom(r)))1∕2| over all r∈[0,π] is computationally infeasible and so we instead calculate it for $r\in R=\left\{r_{1},\ldots ,r_{m}\right\},\;m\in N$, where R is a finite set of distinct, evenly spaced points such that $r_{i}\in \left[0,\pi \right],i=1,\ldots ,m$, for the purposes of our simulation studies. We then take our test statistic as

T1=maxr∈R|P~inhom(r)|,T2=maxr∈R|K~inhom(r)−2π(1−cos(r))Var^(K~inhom(r))|.

where for these simulation studies we set R={0,0.02,0.04,…,π}. These simulations are tested at a 5% significance level. Each experiment is repeated 1000 times, and for each experiment we simulate 999 Poisson processes to approximate the critical values of the hypothesis test.

### 9.3 Results

Table 1, Table 2, Table 3 outline the parameter selection and results of our simulations. By the nature of Monte Carlo simulations the CSR results given in Table 1 are as to be expected with an empirical rejection rate close to 0.05. Expectedly, we see that for the same ellipsoid i.e. a kept constant, that when the Matérn II parameter R increases and the Thomas process parameter κ decreases (each representing an increased departure from CSR), the power of our test improves. In the Section S6.3 of the Supplementary Material we discuss a potential reason for the power of our test decreasing as a decreases (hence c increases), for both regular and cluster processes, for the same R and κ respectively. Additionally, Fig. 5 suggests we may gain power by considering a two sided test.

**Table 1:** Results when the *observed* data is CSR. The semi-major axis length along the x-axis, a, and y-axis, b, are equivalent and the semi-major axis length along the z-axis is determined such that the area of the ellipsoid is 4π.

Experiment No.	Expectation	a	ρ	Reject $H_{0}:T_{1}$	Reject $H_{0}:T_{2}$
1a	40π	1	10	0.0250	0.0480
1b	40π	0.8	10	0.0390	0.0390
1c	40π	0.6	10	0.0440	0.0430
1d	40π	0.4	10	0.0560	0.0560

**Table 2:** Results when the *observed* data is a Matérn II process, with independent mark being exponential with rate 1. The semi-major axis length along the x-axis, a, and y-axis, b, are equivalent and the semi-major axis length along the z-axis is determined such that the area of the ellipsoid is 4π. Fixing the expectation, μ, and hard-core distance, R, we use Eq. (26) to calculate ρ for the underlying constant Poisson process intensity function. When R=0 a Matérn II process collapses to a CSR process.

Experiment No.	Expectation	a	R	Reject $H_{0}:T_{1}$	Reject $H_{0}:T_{2}$
2ai	100	1	0	0.0450	0.0750
2aii	100	1	0.05	0.2520	0.0270
2aiii	100	1	0.1	1.0000	0.4550
2aiv	100	1	0.2	1.0000	1.0000

2bi	100	0.8	0	0.0440	0.0550
2bii	100	0.8	0.05	0.0460	0.0030
2biii	100	0.8	0.1	0.8460	0.0370
2biv	100	0.8	0.2	1.0000	1.0000

2ci	100	0.6	0	0.0520	0.0510
2cii	100	0.6	0.05	0.0540	0.0060
2ciii	100	0.6	0.1	0.0260	0.0010
2civ	100	0.6	0.2	0.2990	0.7790

2di	100	0.4	0	0.0440	0.0410
2dii	100	0.4	0.05	0.0400	0.0100
2diii	100	0.4	0.1	0.0270	0.0000
2div	100	0.4	0.2	0.0020	0.0020

**Table 3:** Results when the *observed* data is an ellipsoidal Thomas process. The expected number of offspring per parent is λ=20 and the underlying Poisson parent process has constant intensity function ρ=μ∕(4πλ), where μ is the expectation. The semi-major axis length along the x-axis, a, and y-axis, b, are equivalent and the semi-major axis length along the z-axis is determined such that the area of the ellipsoid is 4π. When κ=∞ an ellipsoidal Thomas process collapses to a CSR process.

Experiment No.	Expectation	a	κ	Reject $H_{0}:T_{1}$	Reject $H_{0}:T_{2}$
3ai	150	1	∞	0.0290	0.0440
3aii	150	1	5	0.0330	0.0470
3aiii	150	1	1	0.4620	0.5630
3aiv	150	1	0.5	0.9840	0.9830

3bi	150	0.8	∞	0.0460	0.0540
3bii	150	0.8	5	0.0530	0.0570
3biii	150	0.8	1	0.2950	0.2120
3biv	150	0.8	0.5	0.9260	0.9340

3ci	150	0.6	∞	0.0490	0.0460
3cii	150	0.6	5	0.0570	0.0610
3ciii	150	0.6	1	0.3730	0.1400
3civ	150	0.6	0.5	0.7480	0.7800

3di	150	0.4	∞	0.0670	0.0600
3dii	150	0.4	5	0.0530	0.0360
3diii	150	0.4	1	0.4460	0.2020
3div	150	0.4	0.5	0.6460	0.6350

We see that $T_{1}$ achieves greater empirical power compared to $T_{2}$ over the majority of experiments considered in our simulation study. This is clearly the case when a=1 or 0.8 for the Matérn II process whilst when a=0.6 or 0.4 the distinction is not as clear. In particular, $T_{2}$ achieves considerably greater empirical power for Experiment (2civ). For the Thomas process we see that $T_{1}$ out performs $T_{2}$ in nearly all experiments for all values of a. These result suggest the following considerations in practice: (1) consider $T_{1}$ before $T_{2}$ since computing $T_{1}$ is simpler, (2) if $T_{1}$ does not provide sufficient evidence against the null then $T_{2}$ may provide additional information or even considering another functional summary statistic that has been developed in this work. Although a formal hypothesis test may be sought, these results emphasise that this should not be without a detailed examination of simulation envelope plots which can potentially provide further information. In particular, simulation envelope plots can give indications as to whether the point pattern exhibits more regular or clustered behaviour (Diggle, 2003).

## 10 Discussion & conclusion

In this work we have discussed point patterns observed on arbitrary, bounded convex shapes in $R^{3}$, motivated by the need for such exploratory analyses in the area of microbiology. We have highlighted the challenge of handling such spaces due to the lack of isometries for such objects. Using the invariance of Poisson processes (Kingman, 1993), we can circumvent this lack of isometries in the original space by mapping to the sphere which has rotational symmetries. By doing so we propose a set of functional summary statistics for the class of Poisson processes. Further to this we have also proposed functional summary statistics for CSR processes on the convex space and explored their properties. Using this we have, in turn, been able to construct test statistics which can be used to reject the hypothesis of CSR for observed point patterns. We have also conducted simulation studies to investigate the effectiveness off the proposed test statistics in rejecting the null hypothesis when the observed data is either regular or clustered.

Interesting extensions to this work would include relaxing the need for convexity of the shape of interest. This presents a significant challenge as how one constructs the required mapping is not obvious. Another consideration is how to construct an estimator of the intensity function on D. One approach might be to construct it on $S^{2}$ and inverse map to D. There is, of course, the open question of how one forms summary statistics for multivariate point processes on convex shapes. Answering this would have immediate impact in bioimaging applications where experimentalists are regularly interested in spatial dependencies that exist between two or more different types of molecules.

## Appendix A Supplementary data

MMC S1
