# Supplementary material for: Testing for complete spatial randomness on three dimensional bounded convex shapes
Source: Spat Stat. 2021 Mar;41:100489. doi: 10.1016/j.spasta.2020.100489 (PMC7903323; doi:10.1016/j.spasta.2020.100489)
Supplement: MMC S1 [file mmc1.pdf]

Supplementary Material for Testing for complete spatial randomness on three dimensional bounded convex shapes

Scott Ward[a], Edward A.K. Cohen[a,*], Niall Adams[a,b]

[a] *Deparment of Mathematics, Imperial College London, South Kensington, London, SW7 2AZ, United Kingdom*
[b] *Data Science Institute, Imperial College London, South Kensington, London, SW7 2AZ, United Kingdom*

Outline

This supplementary material is in support of this paper. A brief outline of what is to follow is given below:

S1 A more general proof of the infinite series representation for the F-, and H-functions given by White [1].

S2 Poissonial invariance for Poisson processes on a convex shape in $\mathbb{R}^3$ mapped to $\mathbb{S}^2$.

S3 Derivation of the expectations of the functional summary statistics: F_{inhom}-, H_{inhom}-, and K_{inhom}-functions.

S4 Derivation of the variances of the functional summary statistics: F_{inhom}-, H_{inhom}-, and K_{inhom}-functions.

S5 Existence and approximations to the first and second order moments of the $\hat{J}_{inhom}$-function are given.

S6 Discussion of regular and cluster processes on convex three dimensional shapes.

S7 Derivation of the moments for the estimator of the K_{inhom}-function when ρ is unknown.

⋆This document is the results of the research project funded by the Wellcome Trust.

*Corresponding author

Email addresses: scott.ward12@imperial.ac.uk (Scott Ward), e.cohen@imperial.ac.uk (Edward A.K. Cohen), n.adams@imperial.ac.uk (Niall Adams)

S1. Infinite series representation of the F-, and H-functions

In this section we derive an infinite series representation for the F-, and H-function. White [1] was the first to derive these in $\mathbb{R}^d$. We provide a derivation on $\mathbb{S}^2$ assuming the existence of the nth-order factorial moment measures for all $n \in \mathbb{N}$. We also provide an identity when the nth-order product densities do exists for all $n \in \mathbb{N}$. Before giving the results we first introduce the nth-order factorial moment measures, $\alpha^{(n)} : \mathbb{S}^2 \times \cdots \times \mathbb{S}^2 \mapsto \mathbb{R}$, as,

$$\alpha^{(n)}(B_1 \times \cdots \times B_n) = \mathbb{E} \sum_{\mathbf{x}_1,\dots,\mathbf{x}_n \in X}^{\neq} \mathbb{1}[\mathbf{x}_1 \in B_1, \dots, \mathbf{x}_n \in B_n],$$

for $B_i \subseteq \mathbb{S}^2,\ i = 1, \dots, n$. Then if $\alpha^{(n)}$ is absolutely continuous with respects to the Lebesgue measure, there then exists $\rho^{(n)}$ such that,

$$\alpha^{(n)}(B_1 \times \cdots \times B_n) = \int_{B_1} \cdots \int_{B_n} \rho^{(n)}(\mathbf{x}_1, \dots, \mathbf{x}_n)\lambda_{\mathbb{S}^2}(d\mathbf{x}_1) \cdots \lambda_{\mathbb{S}^2}(d\mathbf{x}_n), \tag{1}$$

then $\rho^{(n)}$ is the nth-order product intensity function [2].

Theorem 1. *Let X be an isotropic spheroidal point process with constant intensity function ρ. Further we assume the existence of all n^{th}-order factorial moment measures for both X and its reduced Palm process, $X^!_{\mathbf{x}}$. Then the F- and H-functions have the following series representation,*

$$F(r) = -\sum_{n=1}^{\infty} \frac{(-1)^n}{n!}\alpha^{(n)}(B_{\mathbb{S}^2}(\mathbf{o}, r), \dots, B_{\mathbb{S}^2}(\mathbf{o}, r))$$
$$H(r) = -\sum_{n=1}^{\infty} \frac{(-1)^n}{n!}\alpha^{!(n)}_{\mathbf{o}}(B_{\mathbb{S}^2}(\mathbf{o}, r) \dots, B_{\mathbb{S}^2}(\mathbf{o}, r))$$

where $\alpha^{(n)}$ and $\alpha^{!(n)}_{\mathbf{x}}$ are the factorial moment measure for X and $X^!_{\mathbf{x}}$ and $B_{\mathbb{S}^2}(\mathbf{o}, r)$ is the spherical cap of radius r at the origin $\mathbf{o} \in \mathbb{S}^2$. These representations hold provided the series is absolutely convergent, that is if $\lim_{n\to\infty} |a_{n+1}/a_n| < 1$ *or* $\limsup_{n\to\infty}(|a_n|)^{1/n} < 1$, *where $a_n = ((-1)^n/n!)\alpha^{(n)}(B_{\mathbb{S}^2}(\mathbf{o}, r), \dots, B_{\mathbb{S}^2}(\mathbf{o}, r))$ for the F-function or $a_n = ((-1)^n/n!)$ $\alpha^{!(n)}_{\mathbf{o}}(B_{\mathbb{S}^2}(\mathbf{o}, r) \dots, B_{\mathbb{S}^2}(\mathbf{o}, r))$ for the H-function.*

Proof. Let $\{B_1, \ldots, B_n\}$ be a partition of $B_{\mathbb{S}^2}(\mathbf{o}, r)$ into n sets, such that as n increases the area of each B_i decreases. Then,

$$\begin{aligned}
F(r) &= 1 - P_{X \cap B_{\mathbb{S}^2}(\mathbf{o},r)}(\emptyset) \\
&= 1 - P((X_{B_1} = \emptyset) \cap \cdots \cap (X_{B_n} = \emptyset)) \\
&= 1 - \left(1 - \sum_{i=1}^{n} P(X_{B_i} \neq \emptyset) + \sum_{\substack{i,j=1 \\ i<j}}^{n} P((X_{B_i} \neq \emptyset) \cap (X_{B_j} \neq \emptyset)) - \cdots \right) \qquad (2) \\
&= \sum_{i=1}^{n} P(X_{B_i} \neq \emptyset) - \sum_{\substack{i,j=1 \\ i<j}}^{n} P((X_{B_i} \neq \emptyset) \cap (X_{B_j} \neq \emptyset)) + \cdots , \qquad (3)
\end{aligned}$$

where the (2) follows from the inclusion-exclusion principle. Next we shall consider the first term. Define $a_n = \sum_{i=1}^{n} \mathbb{1}[X_{B_i} \neq \emptyset]$ and $a = \sum_{\mathbf{x} \in X} \mathbb{1}[\mathbf{x} \in B_{\mathbb{S}^2}(\mathbf{o}, r)]$. Then it can easily be seen that a_n is a monotonically increasing sequence since as the number of partitions increases the number of partitions containing more than one point of X decreases. Further the maxmimum of a_n is a. To see this consider small neighbourhoods of each $\mathbf{x} \in X$ such that these neighbourhoods are all disjoint (this is possible since X is a simple process), label these $B_{\mathbf{x}}$, then $\mathbb{1}[X_{B_{\mathbf{x}}} \neq \emptyset] = 1$, $\forall \mathbf{x} \in X$ and so for this partition $a_n = a$ and cannot increase as this would require at least one point of X to be in two different disjoint $B_{\mathbf{x}}$: a contradiction. Hence as $n \to \infty$, $a_n \to a$. Therefore,

$$\begin{aligned}
\sum_{i=1}^{n} P(X_{B_i} \neq \emptyset) &= \mathbb{E} \sum_{i=1}^{n} \mathbb{1}[X_{B_i} \neq \emptyset] \\
&= \int_{N_{lf}} \sum_{i=1}^{n} \mathbb{1}[x \neq \emptyset] dP(x).
\end{aligned}$$

Since $a_n \leq a$ and by assumption $\alpha^{(1)} \equiv \alpha$ exists $\mathbb{E}[a] = \mathbb{E} \sum_{\mathbf{x} \in X} \mathbb{1}[\mathbf{x} \in B_{\mathbb{S}^2}(\mathbf{o}, r)] = \alpha(B_{\mathbb{S}^2}(\mathbf{o}, r)) < \infty$, we can therefore apply the dominated convergence theorem when taking the limit as n increases and the volumes of B_i decrease, i.e.

$$\begin{aligned}
\lim_{\substack{n \to \infty \\ |B_i| \to 0}} \sum_{i=1}^{n} P(X_{B_i} \neq \emptyset) &= \lim_{\substack{n \to \infty \\ |B_i| \to 0}} \int_{N_{lf}} \sum_{i=1}^{n} \mathbb{1}[x \neq \emptyset] dP(x) \\
&= \int_{N_{lf}} \lim_{\substack{n \to \infty \\ |B_i| \to 0}} \sum_{i=1}^{n} \mathbb{1}[x \neq \emptyset] dP(x)
\end{aligned}$$

$$= \int_{N_{lf}} \sum_{\mathbf{y} \in x} \mathbb{1}[y \in B_{\mathbb{S}^2}(\mathbf{o}, r)] dP(x)$$

$$= \mathbb{E} \sum_{\mathbf{x} \in X} \mathbb{1}[x \in B_{\mathbb{S}^2}(\mathbf{o}, r)]$$

$$= \alpha(B_{\mathbb{S}^2}(\mathbf{o}, r))$$

An identical approach can be used for the remaining terms of 3. By considering the kth term of 3 we have,

$$(-1)^{k+1} \sum_{\substack{i_1,\dots,i_k=1 \\ i_1<\cdots<i_k}}^{n} P((X_{B_{i_1}} \neq \emptyset) \cap \cdots \cap (X_{B_{i_k}} \neq \emptyset))$$

$$= \frac{(-1)^{k+1}}{k!} \sum_{i_1=1}^{n} \cdots \sum_{\substack{i_k=1 \\ i_k \notin \{i_1,\dots,i_{k-1}\}}}^{n} P((X_{B_{i_1}} \neq \emptyset) \cap \cdots \cap (X_{B_{i_k}} \neq \emptyset)).$$

By defining $a_n = \sum^{\neq}_{i_1,\dots,i_k \in \{1,\dots,n\}} \mathbb{1}[X_{B_{i_1}} = \emptyset, \dots, X_{B_{i_k}} = \emptyset]$ and $a = \sum^{\neq}_{\mathbf{x}_1,\dots,\mathbf{x}_k \in X} \mathbb{1}[\mathbf{x}_1 \in B_{\mathbb{S}^2}(\mathbf{o}, r), \dots, \mathbf{x}_k \in B_{\mathbb{S}^2}(\mathbf{o}, r)]$ identical arguments can be made as in the case for $k = 1$ giving,

$$\lim_{\substack{n \to \infty \\ |B_i| \to 0}} (-1)^{k+1} \sum_{\substack{i_1,\dots,i_k=1 \\ i_1<\cdots<i_k}}^{n} P((X_{B_{i_1}} \neq \emptyset) \cap \cdots \cap (X_{B_{i_k}} \neq \emptyset)) = \frac{-(-1)^k}{k!} \alpha^{(k)}(B_{\mathbb{S}^2}(\mathbf{o}, r), \dots, B_{\mathbb{S}^2}(\mathbf{o}, r)),$$

and so gives the series for the F-function.

The series representation for the H-function follows an identical argument to that of the F-function, instead using the factorial moment measure for the reduced Palm point process, $X^!_{\mathbf{x}}$. □

The following corollary shows that the infinite series for the F-, and H-functions can also be represented using the nth-order product densities, used by van Lieshout [3].

Corollary 1. *(White [1]) Under the same assumptions as Theorem 1, let X be an isotropic spheroidal point process with constant intensity function ρ. Further we assume the existence of all n^{th}-order product intensities for both X and its reduced Palm process, $X^!_{\mathbf{x}}$. Then the F- and H-functions have the following series representation,*

$$F(r) = -\sum_{n=1}^{\infty} \frac{(-1)^n}{n!} \int_{B_{\mathbb{S}^2}(\mathbf{o},r)} \cdots \int_{B_{\mathbb{S}^2}(\mathbf{o},r)} \rho^{(n)}(\mathbf{x}_1, \dots, \mathbf{x}_n) \lambda_{\mathbb{S}^2}(d\mathbf{x}_1) \cdots \lambda_{\mathbb{S}^2}(d\mathbf{x}_n)$$

$$H(r) = -\sum_{n=1}^{\infty} \frac{(-1)^n}{n!} \int_{B_{\mathbb{S}^2}(\mathbf{o},r)} \cdots \int_{B_{\mathbb{S}^2}(\mathbf{o},r)} \frac{\rho^{(n+1)}(\mathbf{o}, \mathbf{x}_1, \ldots, \mathbf{x}_n)}{\rho} \lambda_{\mathbb{S}^2}(d\mathbf{x}_1) \cdots \lambda_{\mathbb{S}^2}(d\mathbf{x}_n)$$

provided the series is absolutely convergent, where $B_{\mathbb{S}^2}(\mathbf{o}, r)$ is the spherical cap of radius r at the origin $\mathbf{o} \in \mathbb{S}^2$.

Proof. The infinite series for F follows immediately from Theorem 1 and Equation 1. Further, by assumption we also know that,

$$\alpha_{\mathbf{o}}^{!(n)}(B_1 \times \cdots \times B_n) = \int_{B_1} \cdots \int_{B_n} \rho_{\mathbf{o}}^{!(n)}(\mathbf{x}_1, \ldots, \mathbf{x}_n) \lambda_{\mathbb{S}^2}(d\mathbf{x}_1) \cdots \lambda_{\mathbb{S}^2}(d\mathbf{x}_n).$$

Then we have the following relationship between the nth-order product intensities of X and $X_{\mathbf{o}}^{!}$ (see for example Coeurjolly et al. [2]),

$$\rho_{\mathbf{x}}^{!(n)}(\mathbf{x}_1, \ldots, \mathbf{x}_m) = \frac{\rho^{(n+1)}(\mathbf{x}, \mathbf{x}_1, \ldots, \mathbf{x}_n)}{\rho(\mathbf{x})},$$

and so for our point process X,

$$\alpha_{\mathbf{o}}^{!(n)}(B_1 \times \cdots \times B_n) = \int_{B_1} \cdots \int_{B_n} \frac{\rho_{\mathbf{o}}^{(n+1)}(\mathbf{o}, \mathbf{x}_1, \ldots, \mathbf{x}_n)}{\rho} \lambda_{\mathbb{S}^2}(d\mathbf{x}_1) \cdots \lambda_{\mathbb{S}^2}(d\mathbf{x}_n),$$

and then by Theorem 1 we have the infinite series for the H-function in terms of the nth-order product intensities. □

S2. Poissonial Invariance

In this section we show that a Poisson process lying on an arbitrary bounded convex space $\mathbb{D} \subset \mathbb{R}^3$ can be mapped to another Poisson process on a sphere, known as the Mapping Theorem Kingman [4]. We also show that no two different Poisson processes on $\mathbb{D}$ map to the same Poisson process on the sphere under the same mapping.

Before starting the main theorem of this section we first introduce a lemma (see for example Møller and Waagepetersen [5, Proposition 3.1, pp 15-16]) which is an expansion of the probability measure for a Poisson process on an arbitrary metric space. We shall state it in the context of an arbitrary convex shape in $\mathbb{R}^3$, represented by $\mathbb{D}$.

Lemma S1. *(Møller et al. [5]) X is a Poisson process with intensity function $\rho : \mathbb{D} \mapsto \mathbb{R}$ on $\mathbb{D}$ if and only if for all $B \subseteq \mathbb{D}$ with $\mu(B) = \int_B \rho(\mathbf{x})d\mathbf{x} < \infty$ and all $F \subseteq N_{lf}$,*

$$P(X_B \in F) = \sum_{n=0}^{\infty} \frac{\exp(-\mu(B))}{n!} \int_B \cdots \int_B \mathbb{1}[\{\mathbf{x}_1, \ldots, \mathbf{x}_n\} \in F] \prod_{i=1}^{n} \rho(\mathbf{x}_i)\lambda_{\mathbb{D}}(d\mathbf{x}_1) \cdots \lambda_{\mathbb{D}}(d\mathbf{x}_n), \tag{4}$$

where the integral for $n = 0$ is read as $\mathbb{1}[\emptyset \in F]$.

Proof. See Proposition 3.1 of Møller and Waagepetersen [5]. □

The following lemma shows that the function $f(\mathbf{x}) = \mathbf{x}/||\mathbf{x}||$ is bijective and hence has a well-defined inverse.

Lemma 1. *Let $\mathbb{D}$ be a convex subspace of $\mathbb{R}^3$ such that the origin in $\mathbb{R}^3$ is in the interior of $\mathbb{D}$, i.e. $\mathbf{o} \in \mathbb{D}_{int}$. Then the function $f(\mathbf{x}) = \mathbf{x}/||\mathbf{x}||, f : \mathbb{D} \mapsto \mathbb{S}^2$ is bijective.*

Proof. To show that f is bijective we need to show that it is both injective and surjective. For surjectivity we need to show that for any $\mathbf{x}' \in \mathbb{S}^2$, there exists $\mathbf{x} \in \mathbb{D}$ such that $f(\mathbf{x}) = \mathbf{x}'$. To do this first fix any $\mathbf{x}' \in \mathbb{S}^2$ and define the half line, $L_{\mathbf{x}'} = \{\mathbf{y} \in \mathbb{R}^3 : \mathbf{y} = \lambda\mathbf{x}',\ \lambda \in \mathbb{R}^+\}$, where $\mathbb{R}^+$ is the positive real line including 0. Then since $\mathbb{D}$ is compact (i.e. closed and bounded) then the half line must intersect the $\mathbb{D}$ and so there exists $\mathbf{x} \in \mathbb{D}$ such that $\mathbf{x} \in L_{\mathbf{x}'}$. Therefore there must exists $\lambda \in \mathbb{R}^+, \mathbf{x} = \lambda\mathbf{x}'$. Taking norms of both sides and noting that since $\mathbf{x}' \in \mathbb{S}^2$ meaning $||\mathbf{x}'|| = 1$ then $\lambda = ||\mathbf{x}||$ and so, $\mathbf{x}/||\mathbf{x}|| = \mathbf{x}'$ and so f is surjective.

For injectivity we need to show that for any $\mathbf{x}, \mathbf{y} \in \mathbb{D}$ if $f(\mathbf{x}) = f(\mathbf{y})$ then $\mathbf{x} = \mathbf{y}$. Fix $\mathbf{x}, \mathbf{y} \in \mathbb{D}$ such that $f(\mathbf{x}) = f(\mathbf{y})$ and define $\mathbf{x}' = f(\mathbf{x}) = f(\mathbf{y})$. Again define the line $L_{\mathbf{x}'}$ as previous and by convexity of $\mathbb{D}$, the fact that $\mathbf{o} \in \mathbb{D}$ and since $L_{\mathbf{x}'}$ is a half line then there is precisely only one intersection of $L_{\mathbf{x}'}$ with $\mathbb{D}$. Therefore both $\mathbf{x}$ and $\mathbf{y}$ must be this point of intersection meaning $\mathbf{x} = \mathbf{y}$. Hence f is bijective. □

Now we give the main theorem of our work which shows that a Poisson process on $\mathbb{D}$ is mapped to a Poisson process on $\mathbb{S}^2$. This is known as the Mapping Theorem [4]. Here we use Lemma S1.

Before beginning this theorem we lay down a little notation in order to avoid confusion. $\mathbf{x} = (x, y, z)$ will be an element of $\mathbb{R}^3$ where we may subscript with an $n \in \mathbb{N}$ when we are referring to a single vector within a set. x will be an element of N_{lf} and may also be subscripted with $n \in \mathbb{N}$ when we are referring to a single element in a set of finite point configurations. Notice that we are using x to be both the first element of $\mathbf{x}$ and an element in N_{lf}, based on context it will be clear to which we are referring too.

Theorem 2. *Let X be a Poisson process on an arbitrary bounded convex shape $\mathbb{D} \subset \mathbb{R}^3$ with intensity function $\rho : \mathbb{D} \mapsto \mathbb{R}$. We assume that $\mathbb{D} = \{\mathbf{x} \in \mathbb{R}^3 : g(\mathbf{x}) = 0\}$ where $g(\mathbf{x}) = 0$ is the zero set function and is defined as,*

$$g(\mathbf{x}) = \begin{cases} g_1(\mathbf{x}) = 0, & \mathbf{x} \in \mathbb{D}_1 \\ & \vdots \\ g_n(\mathbf{x}) = 0, & \mathbf{x} \in \mathbb{D}_n \end{cases}$$

such that $\cup_{i=1}^{n} \mathbb{D}_i = \mathbb{D}$ and $\mathbb{D}_i \cap \mathbb{D}_j = \emptyset$, $\forall i \neq j$. Then define $Y = f(X)$, where $f(\mathbf{x}) = \mathbf{x}/||\mathbf{x}||$ and we have taken the convention that $f(X) = \{\mathbf{y} \in \mathbb{S}^2 : \mathbf{y} = \mathbf{x}/||\mathbf{x}||, \mathbf{x} \in X\}$. Then Y is a Poisson process on $\mathbb{S}^2$, with intensity function,

$$\rho^*(\mathbf{x}) = \begin{cases} \rho(f^{-1}(\mathbf{x})) l_1(f^{-1}(\mathbf{x})) J_{(1,f^*)}(\mathbf{x}) \sqrt{1 - x^2 - y^2}, & \mathbf{x} \in f(\mathbb{D}_1) \\ \vdots & \\ \rho(f^{-1}(\mathbf{x})) l_n(f^{-1}(\mathbf{x})) J_{(n,f^*)}(\mathbf{x}) \sqrt{1 - x^2 - y^2}, & \mathbf{x} \in f(\mathbb{D}_n) \end{cases}$$

where,

$$
\begin{aligned}
z &= \tilde{g}_i(x,y)\\
l_i(\mathbf{x}) &= \left[1+\left(\frac{\partial \tilde{g}_i}{\partial x}\right)^2+\left(\frac{\partial \tilde{g}_i}{\partial y}\right)^2\right]^{\frac{1}{2}}\\
J_{(i,f^{*-1})}(\mathbf{x}) &= \frac{1}{(x^2+y^2+\tilde{g}_i^2(x,y))^3}\\
&\det\left[\begin{pmatrix} y^2+\tilde{g}_i^2(x,y)-x\tilde{g}_i(x,y)\frac{\partial \tilde{g}_i}{\partial x} & -x\left(y+\tilde{g}_i(x,y)\frac{\partial \tilde{g}_i}{\partial y}\right)\\ -y\left(x+\tilde{g}_i(x,y)\frac{\partial \tilde{g}_i}{\partial x}\right) & x^2+\tilde{g}_i^2(x,y)-y\tilde{g}_i(x,y)\frac{\partial \tilde{g}_i}{\partial y}\end{pmatrix}\right]\\
J_{(i,f^*)}(\mathbf{x}) &= \frac{1}{J_{(i,f^{*-1})}(f^{-1}(\mathbf{x}))},
\end{aligned}
$$

where $\mathbf{x}=(x,y,z)^T$*,* f^{-1} *is the inverse of* f*,* $\det(\cdot)$ *is the determinant operator, and* $f^*:\mathbb{R}^2\mapsto\mathbb{R}^2$ *is the function which maps* $x\mapsto x/||\mathbf{x}||$ *and* $y\mapsto y/||\mathbf{x}||$*.*

Proof. In order to show that $Y\equiv f(X)$ is a Poisson process we show that its distribution function can be expanded as given by Equation 4. Then $\forall B\subseteq\mathbb{D}$ and $\forall F\subseteq N_{lf}$ and noting that f is a measurable map (since the map is bijective and hence an inverse exists) we have that,

$$
\begin{aligned}
P(Y_B\in F) &= P(f^{-1}(Y_B)\in f^{-1}(F))\\
&= P(X_{f^{-1}(B)}\in f^{-1}(F))
\end{aligned}
$$

Now we define $f_i^{-1}(F)$, for $i=1,\dots,n$ where $F\subseteq N_{lf}$,

$$
\begin{aligned}
F = &\quad \{x\in N_{lf}: x=\{\mathbf{x}_1,\dots,\mathbf{x}_m\}, m\in\mathbb{N}, x\in F\}\\
f^{-1}(F) = &\quad \{f^{-1}(x): f^{-1}(x)=\{f^{-1}(\mathbf{x}_1),\dots,f^{-1}(\mathbf{x}_m)\}, m\in\mathbb{N}, x\in F\}
\end{aligned}
$$

we want to partition $f^{-1}(F)$ over each $f^{-1}(\mathbb{D}_i), i=1,\dots,n$

$$
\begin{aligned}
f^{-1}(F) = &\quad \big\{\cup_{i=1}^n f^{-1}(x_i): f^{-1}(x_i)=\{f^{-1}(\mathbf{x}_{(i,1)}),\dots,f^{-1}(\mathbf{x}_{(i,m_i)})\},\\
&\quad f^{-1}(\mathbf{x}_{(i,j)})\in f^{-1}(\mathbb{D}_i), j=1,\dots,m_i,\ m_i\in\mathbb{N},\ i=1,\dots,n, \cup_{i=1}^n x_i\in F\big\}.
\end{aligned}
$$

To understand the notation $\mathbf{x}_{(i,j)}$ consider first a single element $x\in F$. Then since F is a subset of N_{lf} this means that $|x|\in\mathbb{N}$. Then define $m_i=|x\cap\mathbb{D}_i|$, hence $\sum_{i=1}^n m_i=|x|$.

Then $\mathbf{x}_{(i,j)}$ is j^{th} element of $x \cap \mathbb{D}_i$ such that $j = 1, \ldots, m_i$. We define $f_i^{-1}(F) \equiv \{f^{-1}(x) : f^{-1}(x) \equiv \{f^{-1}(\mathbf{x}_1), \ldots, f^{-1}(\mathbf{x}_n)\},\ f^{-1}(\mathbf{x}_i) \in f^{-1}(\mathbb{D}_i), n \in \mathbb{N}, \exists y \in F \text{ such that } x \subseteq y\}$. Then,

$$\begin{aligned}
P(X_{f^{-1}(B)} \in f^{-1}(F)) &= P(\{X_{f^{-1}(B)\cap\mathbb{D}_1}, \ldots, X_{f^{-1}(B)\cap\mathbb{D}_n}\} \in f^{-1}(F)) \\
&= P(X_{f^{-1}(B)\cap\mathbb{D}_1} \in f_1^{-1}(F), \ldots, X_{f^{-1}(B)\cap\mathbb{D}_n} \in f_n^{-1}(F)) \\
&= P(X_{f_1^{-1}(B)} \in f_1^{-1}(F), \ldots, X_{f_n^{-1}(B)} \in f_n^{-1}(F)) \\
&= \prod_{i=1}^{n} P(X_{f_i^{-1}(B)} \in f_i^{-1}(F))
\end{aligned}$$

where $f_i^{-1}(A) = \{(x,y,z)^T \in A : (x,y,z)^T \in \mathbb{D}_i\}$ if $A \subseteq \mathbb{D}$. We emphasize the dual meaning of $f_i^{-1}(A)$ where the defintion depends on the nature of A, i.e. if $A \subseteq \mathbb{D}$ or if $A \subseteq N_{lf}$. Without loss of generality lets suppose that all projections of each $\mathbb{D}_i$ onto $\mathbb{R}^2$ are invertible, if not then we can divide $\mathbb{D}_i$ into further subsets $\cup_{j=1}^{m}\mathbb{D}_{i,j}$ such that the projection of each $\mathbb{D}_{i,j}$ is then invertible. For example an ellipsoid is defined by the zero-set equation $x^2/a^2 + y^2/b^2 + z^2/c^2 = 1$, but if the entire space were projected down to $\mathbb{R}^2$ its inverse does not exists, instead we divide the ellipsoid into the upper and lower hemiellipsoids and then the projections restricted to these segments of the ellipsoids are then invertible. Let us also define the projection of $\mathbb{D}_i$ to $\mathbb{R}^2$ as $P_{\mathbb{D}_i}$. Further, for $\mathbf{x} = (x,y,z)^T$, then $\mathbf{x}$ lies on $\mathbb{D}$ if $g(\mathbf{x}) = 0$, we define $\tilde{g}$ to be the rearrangement of g such that z is a function of x and y, i.e. $z = \tilde{g}(x,y)$. Then by Lemma S1,

$$\begin{aligned}
&P(X_{f_i^{-1}(B)} \in f_i^{-1}(F)) \\
&= \sum_{n=0}^{\infty} \frac{\exp(-\mu(f_i^{-1}(B)))}{n!} \\
&\qquad \int_{f_i^{-1}(B)} \cdots \int_{f_i^{-1}(B)} \mathbb{1}[\{\mathbf{x}_1, \ldots, \mathbf{x}_n\} \in f_i^{-1}(F)] \prod_{i=1}^{n} \rho(\mathbf{x}_i)\lambda_{\mathbb{D}_i}(d\mathbf{x}_1)\cdots\lambda_{\mathbb{D}_i}(d\mathbf{x}_n) \\
&= \sum_{n=0}^{\infty} \frac{\exp(-\mu(f_i^{-1}(B))}{n!} \\
&\qquad \iint_{P_{\mathbb{D}_i}[f_i^{-1}(B)]} \cdots \iint_{P_{\mathbb{D}_i}[f_i^{-1}(B)]} \mathbb{1}[\{(x_1, y_1, \tilde{g}_1(x_1,y_1))^T, \ldots, (x_n, y_n, \tilde{g}_1(x_n,y_n))^T\} \in f_i^{-1}(F)]
\end{aligned}$$

$$\prod_{i=1}^{n} \rho\left(x_i, y_i, \tilde{g}_i(x_i, y_i)\right) \sqrt{1 + \left(\frac{\partial \tilde{g}_i}{\partial x_i}\right)^2 + \left(\frac{\partial \tilde{g}_i}{\partial y_i}\right)^2} dx_i dy_i$$

$$= \sum_{n=0}^{\infty} \frac{\exp(-\mu(f_i^{-1}(B))}{n!}$$

$$\iint_{P_{\mathbb{D}_i}[f_i^{-1}(B)]} \cdots \iint_{P_{\mathbb{D}_i}[f_i^{-1}(B)]} \mathbb{1}[\{(x_1, y_1, \tilde{g}_1(x_1, y_1))^T, \ldots, (x_n, y_n, \tilde{g}_1(x_n, y_n))^T\} \in f_i^{-1}(F)]$$

$$\prod_{i=1}^{n} \rho\left(x_i, y_i, \tilde{g}_i(x_i, y_i)\right) l_i(x_i, y_i) dx_i dy_i,$$

where $l_i(x_i, y_i) = \sqrt{1 + (\partial \tilde{g}_i/\partial x_i)^2 + (\partial \tilde{g}_i/\partial y_i)^2}$. Now consider the indicator term, we need to show that when $x_i \mapsto x_i/||\mathbf{x}||$ and $y_i \mapsto y_i/||\mathbf{x}||$ then $\mathbb{1}[\{\mathbf{x}_1, \ldots, \mathbf{x}_n\} \in f_i^{-1}(F)] \mapsto \mathbb{1}[\{\mathbf{y}_1, \ldots, \mathbf{y}_n\} \in F_i]$, where $F_i = \{x : x = \{\mathbf{x}_1, \ldots, \mathbf{x}_m\}, \mathbf{x}_j \in f(\mathbb{D}_i), j = 1, \ldots, m, m \in \mathbb{N}, \exists y \in F \text{ such that } x \subseteq y\}$. Let us consider first an individual point $\mathbf{x} \in \mathbb{D}$. Further let us define $r = |\mathbf{x}| = \sqrt{x^2 + y^2 + z^2}$. Then since $z = \tilde{g}_i(x, y)$, r is thus a function of x and y, let us write $r(x, y) = ||\mathbf{x}||$. Thus we can rewrite $z = \tilde{g}_i(x, y) = \sqrt{r^2(x, y) - x^2 - y^2}$. Then suppose we apply the transformations $x' = x/r(x, y)$ and $y' = y/r(x, y)$, we have $z = \sqrt{r^2(x, y) - r^2(x, y)x'^2 - r^2(x, y)y'^2} \Rightarrow z = r(x, y)\sqrt{1 - x'^2 - y'^2}$. Therefore,

$$\mathbb{1}[\{\mathbf{x}_1, \ldots, \mathbf{x}_n\} \in f_i^{-1}(F)] = \mathbb{1}[\{(x_1, y_1, \tilde{g}_1(x_1, y_1))^T, \ldots, (x_n, y_n, \tilde{g}_1(x_n, y_n))^T\} \in f_i^{-1}(F)]$$

apply transformations $x' = x/r(x, y)$ and $y' = y/r(x, y)$,

$$= \mathbb{1}\left[\left\{\left(r(x_1, y_1)x'_1, r(x_1, y_1)y'_1, r(x_1, y_1)\sqrt{1 - x'^2_1 - y'^2_1}\right)^T, \ldots, \right.\right.$$
$$\left.\left.\left(r(x_n, y_n)x'_n, r(x_n, y_n)y'_n, r(x_n, y_n)\sqrt{1 - x'^2_n - y'^2_n}\right)^T\right\} \in f_i^{-1}(F)\right]$$
$$= \mathbb{1}\left[\left\{\left(x'_1, y'_1, \sqrt{1 - x'^2_1 - y'^2_1}\right)^T, \ldots, \left(x'_n, y'_n, \sqrt{1 - x'^2_n - y'^2_n}\right)^T\right\} \in F_i\right]$$
$$= \mathbb{1}[\{\mathbf{y}_1, \ldots, \mathbf{y}_n\} \in F_i]$$

Let us return to $P(X_{f_i^{-1}(B)} \in f_i^{-1}(F))$. We apply the transformation $f^*(x, y) = (x/r(x, y), y/r(x, y))^T$ and define the inverse as $f^{*-1}(x, y)$. We have,

$$P(X_{f_i^{-1}(B)} \in f_i^{-1}(F))$$

$$= \sum_{n=0}^{\infty} \frac{\exp(-\mu(f_i^{-1}(B))}{n!} \iint\limits_{f^*(P_{\mathbb{D}_i}[f_i^{-1}(B)])} \cdots \iint\limits_{f^*(P_{\mathbb{D}_i}[f_i^{-1}(B)])} \mathbb{1}[\{\mathbf{y}, \ldots, \mathbf{y}_n\} \in F_i]$$
$$\prod_{i=1}^{n} \left[\rho\left(f_1^{*-1}(x_i', y_i'), f_2^{*-1}(x_i', y_i'), \tilde{g}_i(f_1^{*-1}(x_i', y_i'), f_2^{*-1}(x_i', y_i'))\right)\right.$$
$$\left. l_i(f_1^{*-1}(x_i', y_i'), f_2^{*-1}(x_i', y_i')) J_{(i,f^*)}(x_i', y_i') dx_i' dy_i'\right],$$

where $J_{(i,f^*)}(x_i', y_i')$ is the Jacobian of the transformation f^*. The Jacobian of the transformation is defined as,

$$J_{(i,f^*)}(\mathbf{x}) = \frac{1}{J_{(i,f)}(f^{*-1}(\mathbf{x}))}$$

where we can use the inverse property to obtain $J_{(i,f^{*-1})}$,

$$J_{(i,f^{*-1})}(\mathbf{x}) = \det \left[\begin{pmatrix} \frac{\partial x'}{\partial x} & \frac{\partial x'}{\partial y} \\ \frac{\partial y'}{\partial x} & \frac{\partial y'}{\partial y} \end{pmatrix} \right]$$

The entries of $J_{(i,f^{*-1})}(\mathbf{x})$ are given as follows,

$$\begin{aligned}
\frac{\partial x'}{\partial x} &= \frac{y^2 + \tilde{g}_i^2(x,y) - x\tilde{g}(x,y)\frac{\partial \tilde{g}}{\partial x}}{r^3(x,y)} \\
\frac{\partial x'}{\partial y} &= \frac{-x\left(y + \tilde{g}(x,y)\frac{\partial \tilde{g}}{\partial y}\right)}{r^3(x,y)} \\
\frac{\partial y'}{\partial x} &= \frac{-y\left(x + \tilde{g}(x,y)\frac{\partial \tilde{g}}{\partial x}\right)}{r^3(x,y)} \\
\frac{\partial y'}{\partial y} &= \frac{x^2 + \tilde{g}_i^2(x,y) - y\tilde{g}(x,y)\frac{\partial \tilde{g}}{\partial y}}{r^3(x,y)},
\end{aligned}$$

where $r(x,y) = ||\mathbf{x}||$, and so

$$\begin{aligned}
J_{(i,f^{*-1})}(\mathbf{x}) &= J_{(i,f^{*-1})}(x,y) \\
&= \frac{1}{r^6(x,y)} \det \left[\begin{pmatrix} y^2 + \tilde{g}_i^2(x,y) - x\tilde{g}(x,y)\frac{\partial \tilde{g}}{\partial x} & -x\left(y + \tilde{g}(x,y)\frac{\partial \tilde{g}}{\partial y}\right) \\ -y\left(x + \tilde{g}(x,y)\frac{\partial \tilde{g}}{\partial x}\right) & x^2 + \tilde{g}_i^2(x,y) - y\tilde{g}(x,y)\frac{\partial \tilde{g}}{\partial y} \end{pmatrix} \right].
\end{aligned}$$

Therefore,

$$J_{(i,f^*)}(\mathbf{x'}) = J_{(i,f^*)}(x', y') = \frac{1}{J_{(i,f)}(f_1^{*-1}(x', y'), f_2^{*-1}(x', y'))}$$

Projecting onto the sphere,

$$P(X_{f_i^{-1}(B)} \in f_i^{-1}(F)) = \sum_{n=0}^{\infty} \frac{\exp(-\mu(f_i^{-1}(B))}{n!}$$

$$\int\limits_{P_{\mathbb{S}^2}^{-1}[f^*(P_{\mathbb{D}_i}[f_i^{-1}(B)])]} \cdots \int\limits_{P_{\mathbb{S}^2}^{-1}[f^*(P_{\mathbb{D}_i}[f_i^{-1}(B)])]} \mathbb{1}[\{\mathbf{y}, \ldots, \mathbf{y}_n\} \in F_i] \prod_{i=1}^{n} \rho_i^*(\mathbf{y}_i) \lambda_{\mathbb{S}^2}(d\mathbf{y}_i), \quad (5)$$

where $\rho_i^*(\mathbf{y}) = \rho(f_1^{*-1}(x) \ , f_2^{*-1}(y), \ \tilde{g}_i(f_1^{*-1}(x), \ f_2^{*-1}(y))) \ l_i(f_1^{*-1}(x), f_2^{*-1}(y)) \ J_{i,f^{-1}}(x, y) \sqrt{1 - x^2 - y^2}$, $\mathbf{y} = (x, y, z)^T \in \mathbb{S}^2$.

We now need to show that $P_{\mathbb{S}^2}^{-1}[f^*(P_{\mathbb{D}_i}[f_i^{-1}(B)])] = B \cap f(\mathbb{D}_i)$. Equivalently, we can show that $f^*(P_{\mathbb{D}_i}[f_i^{-1}(B)]) = P_{\mathbb{S}^2}[B \cap f(\mathbb{D}_i)]$. It is easy to see that,

$$f^*(P_{\mathbb{D}_i}[f_i^{-1}(B)]) = \{(x/||\mathbf{x}||, y/||\mathbf{x}||)^2 \in \mathbb{R}^2 : f(\mathbf{x}) \in B \cap f(\mathbb{D}_i), \mathbf{x} = (x, y, z)^T \in \mathbb{D}_i\}$$
$$P_{\mathbb{S}^2}[B \cap f(\mathbb{D}_i)] = \{(x, y) \in \mathbb{R}^2 : (x, y, z)^T \in B \cap f(\mathbb{D}_i)\}.$$

Then since f is bijective (see Lemma 1) this means that for all $\mathbf{x} \in \mathbb{D}$, there exists $\mathbf{y} \in \mathbb{S}^2$ such that $\mathbf{y} = f(\mathbf{x})$. Further since $\mathbb{D}_i, \ i = 1, \ldots, n$ partition $\mathbb{D}$ this means that $f(\mathbb{D}_i), \ i = 1, \ldots, n$ partitions $\mathbb{S}^2$ and so $\mathbf{x} \in f^{-1}(B) \cap \mathbb{D}_i \Rightarrow \mathbf{y} = f(\mathbf{x}) \in B \cap f(\mathbb{D}_i)$. Hence taking the set $P_{\mathbb{S}^2}[B \cap f(\mathbb{D}_i)]$,

$$\begin{aligned}
P_{\mathbb{S}^2}[B \cap f(\mathbb{D}_i)] &= \{(x, y) \in \mathbb{R}^2 : (x, y, z)^T \in B \cap f(\mathbb{D}_i)\} \\
&= \{(x, y) \in \mathbb{R}^2 : (x, y, z)^T = f(\mathbf{x}') \in B \cap f(\mathbb{D}_i), \mathbf{x}' \in \mathbb{D}_i\} \\
&= \{(x, y) \in \mathbb{R}^2 : (x, y, z)^T = (x'/||\mathbf{x}'||, y'/||\mathbf{x}'||, z'/||\mathbf{x}'||)^T \in B \cap f(\mathbb{D}_i), \\
&\qquad \mathbf{x}' = (x', y', z')^T \in \mathbb{D}_i\} \\
&= \{(x'/||\mathbf{x}'||, y'/||\mathbf{x}'||)^T \in \mathbb{R}^2 : (x'/||\mathbf{x}'||, y'/||\mathbf{x}'||, z'/||\mathbf{x}'||)^T \in B \cap f(\mathbb{D}_i), \\
&\qquad \mathbf{x}' = (x', y', z')^T \in \mathbb{D}_i\} \\
&= f^*(P_{\mathbb{D}_i}[f_i^{-1}(B)]).
\end{aligned}$$

Therefore,

$$P(X_{f_i^{-1}(B)} \in f_i^{-1}(F)) = \sum_{n=0}^{\infty} \frac{\exp(-\mu(f_i^{-1}(B))}{n!}$$
$$\int\limits_{B \cap f(\mathbb{D}_i)} \cdots \int\limits_{B \cap f(\mathbb{D}_i)} \mathbb{1}[\{\mathbf{y}, \ldots, \mathbf{y}_n\} \in F_i] \prod_{i=1}^{n} \rho_i^*(\mathbf{y}_i) \lambda_{\mathbb{S}^2}(d\mathbf{y}_i).$$

With this identity we thus have,

$$P(Y_B \in F) = \prod_{i=1}^{n} P(X_{f_i^{-1}(B)} \in f_i^{-1}(F))$$
$$= \prod_{i=1}^{n} \left(\sum_{n=0}^{\infty} \frac{\exp(-\mu(f_i^{-1}(B))}{n!} \int\limits_{B\cap f(\mathbb{D}_i)} \cdots \int\limits_{B\cap f(\mathbb{D}_i)} \mathbb{1}[\{\mathbf{y},\dots,\mathbf{y}_n\} \in F_i] \prod_{i=1}^{n} \rho_i^*(\mathbf{y}_i)\lambda_{\mathbb{S}^2}(d\mathbf{y}_i) \right).$$

Consider the multiplication of two of the multiplicands $i \neq j$. Define $\rho_{i,j}^*(\mathbf{x}) = \rho_i^*(\mathbf{x})$ if $\mathbf{x} \in \mathbb{S}^2 \cap f(\mathbb{D}_i)$ and $\rho_{i,j}^*(\mathbf{x}) = \rho_j^*(\mathbf{x})$ if $\mathbf{x} \in \mathbb{S}^2 \cap f(\mathbb{D}_j)$ and also let $k_{p,n}(\{\mathbf{y}_i\}_{i=1}^n) = \mathbb{1}[\{\mathbf{y}_1,\dots,\mathbf{y}_n\} \in F_p] \prod_{i=1}^n \rho_i^*(\mathbf{y}_i)$, for $p = i, j$. Then we have,

$$P(X_{f_i^{-1}(B)} \in f_i^{-1}(F))P(X_{f_j^{-1}(B)} \in f_j^{-1}(F))$$
$$= \left(\sum_{n=0}^{\infty} \frac{\exp(-\mu(f_i^{-1}(B)))}{n!} \int_{B\cap f(\mathbb{D}_i)} \cdots \int_{B\cap f(\mathbb{D}_i)} k_{i,n}(\{\mathbf{y}_i\}_{i=1}^n)\lambda_{\mathbb{S}^2}(d\mathbf{y}_1)\cdots\lambda_{\mathbb{S}^2}(d\mathbf{y}_n) \right)$$
$$\times \left(\sum_{m=0}^{\infty} \frac{\exp(-\mu(f_j^{-1}(B)))}{m!} \int_{B\cap f(\mathbb{D}_j)} \cdots \int_{B\cap f(\mathbb{D}_j)} k_{j,m}(\{\mathbf{y}_i\}_{i=1}^m)\lambda_{\mathbb{S}^2}(d\mathbf{y}_1)\cdots\lambda_{\mathbb{S}^2}(d\mathbf{y}_m) \right)$$
$$= \sum_{n=0}^{\infty}\sum_{m=0}^{\infty} \exp(-\mu(f_i^{-1}(B)))\exp(-\mu(f_j^{-1}(B)))$$
$$\times \left(\frac{1}{n!} \int_{B\cap f(\mathbb{D}_i)} \cdots \int_{B\cap f(\mathbb{D}_i)} k_{i,n}(\{\mathbf{y}_i\}_{i=1}^n)\lambda_{\mathbb{S}^2}(d\mathbf{y}_1)\cdots\lambda_{\mathbb{S}^2}(d\mathbf{y}_n) \right)$$
$$\times \left(\frac{1}{m!} \int_{B\cap f(\mathbb{D}_j)} \cdots \int_{B\cap f(\mathbb{D}_j)} k_{j,m}(\{\mathbf{y}_i\}_{i=1}^m)\lambda_{\mathbb{S}^2}(d\mathbf{y}_1)\cdots\lambda_{\mathbb{S}^2}(d\mathbf{y}_m) \right)$$

we then use the substitution $m' = m + n$,

$$= \sum_{n=0}^{\infty}\sum_{m=n}^{\infty} \exp(-\mu(f_i^{-1}(B)))\exp(-\mu(f_j^{-1}(B)))$$
$$\times \left(\frac{1}{n!} \int_{B\cap f(\mathbb{D}_i)} \cdots \int_{B\cap f(\mathbb{D}_i)} k_{i,n}(\{\mathbf{y}_i\}_{i=1}^n)\lambda_{\mathbb{S}^2}(d\mathbf{y}_1)\cdots\lambda_{\mathbb{S}^2}(d\mathbf{y}_n) \right)$$
$$\times \left(\frac{1}{(m-n)!} \int_{B\cap f(\mathbb{D}_j)} \cdots \int_{B\cap f(\mathbb{D}_j)} k_{j,m-n}(\{\mathbf{y}_i\}_{i=1}^{m-n})\lambda_{\mathbb{S}^2}(d\mathbf{y}_1)\cdots\lambda_{\mathbb{S}^2}(d\mathbf{y}_{m-n}) \right)$$
$$= \sum_{m=0}^{\infty}\sum_{n=0}^{m} \exp(-\mu(f_i^{-1}(B)))\exp(-\mu(f_j^{-1}(B)))\frac{1}{m!}\frac{m!}{n!(m-n)!}$$

$$\times \left(\int_{B\cap f(\mathbb{D}_i)} \cdots \int_{B\cap f(\mathbb{D}_i)} k_{i,n}(\{\mathbf{y}_i\}_{i=1}^{n}) \lambda_{\mathbb{S}^2}(d\mathbf{y}_1)\cdots\lambda_{\mathbb{S}^2}(d\mathbf{y}_n)\right)$$
$$\times \left(\int_{B\cap f(\mathbb{D}_j)} \cdots \int_{B\cap f(\mathbb{D}_j)} k_{j,m-n}(\{\mathbf{y}_i\}_{i=1}^{m-n}) \lambda_{\mathbb{S}^2}(d\mathbf{y}_1)\cdots\lambda_{\mathbb{S}^2}(d\mathbf{y}_{m-n})\right)$$
$$= \sum_{m=0}^{\infty} \frac{\exp(-\mu(f_i^{-1}(B)))\exp(-\mu(f_j^{-1}(B)))}{m!}$$
$$\sum_{n=0}^{m} \binom{m}{n} \left(\int_{B\cap f(\mathbb{D}_i)} \cdots \int_{B\cap f(\mathbb{D}_i)} k_{i,n}(\{\mathbf{y}_i\}_{i=1}^{n}) \lambda_{\mathbb{S}^2}(d\mathbf{y}_1)\cdots\lambda_{\mathbb{S}^2}(d\mathbf{y}_n)\right)$$
$$\times \left(\int_{B\cap f(\mathbb{D}_j)} \cdots \int_{B\cap f(\mathbb{D}_j)} k_{j,m-n}(\{\mathbf{y}_i\}_{i=1}^{m-n}) \lambda_{\mathbb{S}^2}(d\mathbf{y}_1)\cdots\lambda_{\mathbb{S}^2}(d\mathbf{y}_{m-n})\right)$$
$$= \sum_{m=0}^{\infty} \frac{\exp(-\mu(f_i^{-1}(B)))\exp(-\mu(f_j^{-1}(B)))}{m!}$$
$$\sum_{n=0}^{m} \binom{m}{n} \left(\int_{B\cap f(\mathbb{D}_i)} \cdots \int_{B\cap f(\mathbb{D}_i)} k_{i,n}(\{\mathbf{y}_i\}_{i=1}^{n}) \lambda_{\mathbb{S}^2}(d\mathbf{y}_1)\cdots\lambda_{\mathbb{S}^2}(d\mathbf{y}_n)\right)$$
$$\times \left(\int_{B\cap f(\mathbb{D}_j)} \cdots \int_{B\cap f(\mathbb{D}_j)} k_{j,m-n}(\{\mathbf{y}_i\}_{i=n+1}^{m}) \lambda_{\mathbb{S}^2}(d\mathbf{y}_{n+1})\cdots\lambda_{\mathbb{S}^2}(d\mathbf{y}_m)\right)$$
$$= \sum_{m=0}^{\infty} \frac{\exp(-\mu(f_i^{-1}(B)))\exp(-\mu(f_j^{-1}(B)))}{m!}$$
$$\times \left(\int_{B\cap f(\mathbb{D}_i)} + \int_{B\cap f(\mathbb{D}_j)}\right)\cdots$$
$$\left(\int_{B\cap f(\mathbb{D}_i)} + \int_{B\cap f(\mathbb{D}_j)}\right) k_{i,n}(\{\mathbf{y}\}_{i=1}^{n}) k_{j,m-n}(\{\mathbf{y}\}_{i=n+1}^{m}) \lambda_{\mathbb{S}^2}(d\mathbf{y}_1)\cdots\lambda_{\mathbb{S}^2}(d\mathbf{y}_m)$$

Define $k_{i,j,m}(\{\mathbf{y}_i\}_{i=1}^{m}) = \mathbb{1}[\{\mathbf{y}_1,\ldots,\mathbf{y}_n\} \in F_{i,j}] \prod_{i=1}^{n} \rho^*_{i,j}(\mathbf{y}_i)$, where $F_{i,j} = \{x : x = \{\mathbf{x}_1, \ldots, \mathbf{x}_n\}, n \in \mathbb{N}, \mathbf{x}_i \in \mathbb{D}_i \cup \mathbb{D}_j, \exists y \in F \text{ such that } x \subseteq y\}$. Then it can be seen that $k_{i,n}(\{\mathbf{y}\}_{i=1}^{n}) k_{j,m-n}(\{\mathbf{y}\}_{i=n+1}^{m}) = k_{i,j,m}(\{\mathbf{y}_i\}_{i=1}^{m})$ and so,

$$= \sum_{m=0}^{\infty} \frac{\exp(-\mu(f_i^{-1}(B)))\exp(-\mu(f_j^{-1}(B)))}{m!}$$
$$\times \left(\int_{B\cap f(\mathbb{D}_i)} + \int_{B\cap f(\mathbb{D}_j)}\right)\cdots$$
$$\left(\int_{B\cap f(\mathbb{D}_i)} + \int_{B\cap f(\mathbb{D}_j)}\right) k_{i,j,m}(\{\mathbf{y}_i\}_{i=1}^{m}) \lambda_{\mathbb{S}^2}(d\mathbf{y}_1)\cdots\lambda_{\mathbb{S}^2}(d\mathbf{y}_m)$$

$$= \sum_{m=0}^{\infty} \frac{\exp(-\mu(f_i^{-1}(B))) \exp(-\mu(f_j^{-1}(B)))}{m!}$$
$$\times \int_{B \cap (f(\mathbb{D}_i) \cup f(\mathbb{D}_j))} \cdots \int_{B \cap (f(\mathbb{D}_i) \cup f(\mathbb{D}_j))} k_{i,j,m}(\{\mathbf{y}_i\}_{i=1}^m) \lambda_{\mathbb{S}^2}(d\mathbf{y}_1) \cdots \lambda_{\mathbb{S}^2}(d\mathbf{y}_m)$$

Define $B_{i,j} = B \cap (f(\mathbb{D}_i) \cup f(\mathbb{D}_j))$ and $f_{i,j}^{-1}(B) = f_i^{-1}(B) \cup f_j^{-1}(B)$ and noting that $f_i^{-1}(B) \cap f_j^{-1}(B) = \emptyset$,

$$= \sum_{m=0}^{\infty} \frac{\exp(-\mu(f_{i,j}^{-1}(B)))}{m!} \int_{B_{i,j}} \cdots \int_{B_{i,j}} k_{i,j,m}(\{\mathbf{y}_i\}_{i=1}^m) \lambda_{\mathbb{S}^2}(d\mathbf{y}_1) \cdots \lambda_{\mathbb{S}^2}(d\mathbf{y}_m)$$
$$= \sum_{m=0}^{\infty} \frac{\exp(-\mu(f_{i,j}^{-1}(B)))}{m!} \int_{B_{i,j}} \cdots \int_{B_{i,j}} \mathbb{1}[\{\mathbf{y}_1, \ldots, \mathbf{y}_m\} \in F_{i,j}] \prod_{i=1}^{m} \rho_{i,j}^*(\mathbf{y}_i) \lambda_{\mathbb{S}^2}(d\mathbf{y}_1) \cdots \lambda_{\mathbb{S}^2}(d\mathbf{y}_m)$$

We can then repeat this argument for each multiplicand in Equation 5 and reduce the probability measure $P(Y_B \in F)$ to,

$$P(Y_B \in F) = \sum_{n=0} \frac{\exp(-\mu(f^{-1}(B))}{n!} \int_B \cdots \int_B \mathbb{1}[\{\mathbf{y}, \ldots, \mathbf{y}_n\} \in F] \prod_{i=1}^{n} \rho^*(\mathbf{y}_i) d\mathbf{y}_i,$$

where,

$$\rho^*(\mathbf{x}) = \begin{cases} \rho_1^*(\mathbf{x}), & \mathbf{x} \in f(\mathbb{D}_1) \\ & \vdots \\ \rho_n^*(\mathbf{x}), & \mathbf{x} \in f(\mathbb{D}_n). \end{cases} \tag{6}$$

Then defining $\mu^*(B) = \mu(f^{-1}(B))$ we need to show that $\mu^*(B) = \int_B \rho^*(\mathbf{x}) d\mathbf{x}$. This follows since,

$$\begin{aligned} \mu^*(B) &= \mu(f^{-1}(B)) \\ &= \int_{f^{-1}(B)} \rho(\mathbf{x}) d\mathbf{x} \\ &= \sum_{i=1}^{n} \int_{f_i^{-1}(B)} \rho(\mathbf{x}) d\mathbf{x} \\ &= \sum_{i=1}^{n} \int_{B \cap f(\mathbb{D}_j)} \rho_i^*(\mathbf{x}) d\mathbf{x} \\ &= \int_B \rho^*(\mathbf{x}) d\mathbf{x}, \end{aligned}$$

where the penultimate line follows by a the same argument used before, first projecting from $\mathbb{D}_i$ to $\mathbb{R}^2$, then applying the transformation $x' \mapsto x/||\mathbf{x}||$ and $y' \mapsto y/||\mathbf{x}||$, then doing the inverse projection back to the sphere. This finishes the proof by again applying Lemma S1. $\square$

The following corollary shows that two Poisson processes lying on $\mathbb{D}$ with different intensity functions maps to distinct Poisson processes on $\mathbb{S}^2$ under the same transformation, $f(\mathbf{x}) = \mathbf{x}/||\mathbf{x}||$.

Corollary S1. *Suppose that X_1 and X_2 are two Poisson processes on $\mathbb{D}$ with intensity functions ρ_1 and ρ_2 respectively, such that $\rho_1(\mathbf{x}) \neq \rho_2(\mathbf{x})$ for at least one $\mathbf{x} \in \mathbb{D}$. Define the transformed processes $Y_1 = f(X_1)$ and $Y_2 = f(X_2)$ from $\mathbb{D}$ to $\mathbb{S}^2$ where $f(\mathbf{x}) = \mathbf{x}/||\mathbf{x}||$. Then the Y_1 and Y_2 are Poisson processes with intensity functions ρ_1^* and ρ_2^* respectively such that $\rho_1^*(\mathbf{x}) \neq \rho_2^*(\mathbf{x})$ for at least one $\mathbf{x} \in \mathbb{S}^2$.*

Proof. By assumption we suppose there is at least one point in $\mathbb{D}$ such that $\rho_1(\mathbf{x}) \neq \rho_2(\mathbf{x})$. Denote this point $\mathbf{x}^*$ and define $\mathbf{y}^* \equiv f(\mathbf{x}^*) \in f(\mathbb{D}_j)$ for some $j \in \{1, \ldots, n\}$. Then at the point $\mathbf{y}^*$ the intensities of Y_1 and Y_2 are,

$$\rho_1^*(\mathbf{y}^*) = \rho_1(f^{-1}(\mathbf{y}^*))l_1(f^{-1}(\mathbf{y}^*))J_{(1,f^{-1})}(\mathbf{y}^*)\sqrt{1 - y_1^{*2} - y_2^{*2}}$$
$$\rho_2^*(\mathbf{y}^*) = \rho_1(f^{-1}(\mathbf{y}^*))l_1(f^{-1}(\mathbf{y}^*))J_{(1,f^{-1})}(\mathbf{y}^*)\sqrt{1 - y_1^{*2} - y_2^{*2}},$$

respectively. Then $\rho_1^*(\mathbf{y}^*) \neq \rho_2^*(\mathbf{y}^*)$, since $\rho_1(f^{-1}(\mathbf{y}^*)) = \rho_1(\mathbf{x}^*) \neq \rho_2(\mathbf{x}^*) = \rho_2(f^{-1}(\mathbf{y}^*))$. $\square$

The following theorem is the inverse of Theorem 2, where we take $\mathbf{y} = (x, y, z) \in \mathbb{R}^3$.

Theorem S1. *Let X be a Poisson process on $\mathbb{S}^2$ with intensity function $\rho : \mathbb{S}^2 \mapsto \mathbb{R}$. Further let $\mathbb{D} \subset \mathbb{R}^3$ be an arbitrary bounded convex shape that $\mathbb{D} = \{\mathbf{y} \in \mathbb{R}^3 : g(\mathbf{y}) = 0\}$ where $g(\mathbf{y}) = 0$ is the zero set function and is defined as,*

$$g(\mathbf{y}) = \begin{cases} g_1(\mathbf{y}) = 0, & \mathbf{y} \in \mathbb{D}_1 \\ \vdots & \\ g_n(\mathbf{y}) = 0, & \mathbf{y} \in \mathbb{D}_n \end{cases}$$

such that $\cup_{i=1}^{n}\mathbb{D}_i = \mathbb{D}$ *and* $\mathbb{D}_i \cap \mathbb{D}_j = \emptyset$, $\forall i \neq j$. *Then define* $Y = f^{-1}(X)$, *where* $f(\mathbf{y}) = \mathbf{y}/||\mathbf{y}||$ *and we have taken the convention that* $f^{-1}(X) = \{\mathbf{y} \in \mathbb{D} : f(\mathbf{y}) \in X\}$. *Then* Y *is a Poisson process on* $\mathbb{D}$, *with intensity function,*

$$\rho^*(\mathbf{y}) = \begin{cases} \frac{\rho(\mathbf{y})J_{1,f^*-1}(\mathbf{y})}{l_1(\mathbf{y})\sqrt{1-||\mathbf{y}||^2x^2-||\mathbf{y}||^2y^2}}, & \mathbf{y} \in \mathbb{D}_1 \\ & \vdots \\ \frac{\rho(\mathbf{y})J_{n,f^*-1}(\mathbf{y})}{l_n(\mathbf{y})\sqrt{1-||\mathbf{y}||^2x^2-||\mathbf{y}||^2y^2}}, & \mathbf{y} \in \mathbb{D}_n, \end{cases}$$

where,

$$\begin{aligned} z &= \tilde{g}_i(x,y) \\ l_i(\mathbf{y}) &= \left[1 + \left(\frac{\partial \tilde{g}_i}{\partial x}\right)^2 + \left(\frac{\partial \tilde{g}_i}{\partial y}\right)^2\right]^{\frac{1}{2}} \\ J_{(i,f^*-1)}(\mathbf{y}) &= \frac{1}{(x^2+y^2+\tilde{g}_i^2(x,y))^6} \\ &\quad \det\left[\begin{pmatrix} y^2+\tilde{g}_i^2(x,y) - x\tilde{g}_i(x,y)\frac{\partial \tilde{g}_i}{\partial x} & -x\left(y+\tilde{g}_i(x,y)\frac{\partial \tilde{g}_i}{\partial y}\right) \\ -y\left(x+\tilde{g}_i(x,y)\frac{\partial \tilde{g}_i}{\partial x}\right) & x^2+\tilde{g}_i^2(x,y) - y\tilde{g}_i(x,y)\frac{\partial \tilde{g}_i}{\partial y} \end{pmatrix}\right], \end{aligned}$$

with f^{-1} *is the inverse of* f, $\det(\cdot)$ *is the determinant operator, and* $f^* : \mathbb{R}^2 \mapsto \mathbb{R}^2$ *is the function which maps* $x \mapsto x/||\mathbf{y}||$ *and* $y \mapsto y/||\mathbf{y}||$.

Proof. Proof follows identically to the proof of Theorem 2 but following the argument in reverse. □

S3. Expectation of functional summary statistics

In this section we derive the means for the estimators of F_{inhom}-, H_{inhom}-, and K_{inhom}-functions. To do this we will make use of the Campbell-Mecke Theorem [5] on $\mathbb{S}^2$,

Theorem S2. *Let X be a point process on $\mathbb{S}^2$ and h be any non-negative, measurable function such that $h : \mathbb{S}^2 \times N_{lf} \mapsto \mathbb{R}$. Then,*

$$\mathbb{E}\sum_{\mathbf{x}\in X} h(\mathbf{x}, X \setminus \{\mathbf{x}\}) = \int_{\mathbb{S}^2} \mathbb{E}[h(\mathbf{x}, X^!_{\mathbf{x}})]\mu(d\mathbf{x}),$$

and if the intensity function exists then,

$$\mathbb{E}\sum_{\mathbf{x}\in X} h(\mathbf{x}, X \setminus \{\mathbf{x}\}) = \int_{\mathbb{S}^2} \mathbb{E}[h(\mathbf{x}, X^!_{\mathbf{x}})]\rho(\mathbf{x})\lambda_{\mathbb{S}^2}(d\mathbf{x}).$$

Proof. See Møller and Waagepetersen [5, Appendix C, pp. 248-249]. □

Noting that the reduced Palm process, $X^!_{\mathbf{x}}$, for a Poisson process, X, is again the same Poisson process [2] we get the Slivnyak-Mecke Theorem [5, Theorem 3.2, p. 21],

$$\mathbb{E}\sum_{\mathbf{x}\in X} h(\mathbf{x}, X \setminus \{\mathbf{x}\}) = \int_{\mathbb{S}^2} \mathbb{E}[h(\mathbf{x}, X)]\rho(\mathbf{x})\lambda_{\mathbb{S}^2}(d\mathbf{x}).$$

We now prove give the expectation of estimates of the inhomogeneous functional summary statistics,

Theorem 3. *Let X be a spherical Poisson process on $\mathbb{S}^2$ with known intensity function $\rho : \mathbb{S}^2 \mapsto \mathbb{R}_+$, such that $\bar{\rho} = \inf_{\mathbf{x}\in\mathbb{S}^2} \rho(\mathbf{x}) > 0$. Then the estimators for $\hat{F}_{inhom}(r)$, and $\hat{K}_{inhom}(r)$ are unbiased whilst $\hat{H}_{inhom}(r)$ is ratio-unbiased. More precisely,*

$$\begin{aligned}
\mathbb{E}[\hat{F}_{inhom}(r)] &= 1 - \exp(-\bar{\rho}2\pi(1-\cos r)) \\
\mathbb{E}[\hat{H}_{inhom}(r)] &= 1 - \frac{\exp(-\bar{\rho}2\pi(1-\cos r)) - \exp(-\mu(\mathbb{S}^2))}{1 - \frac{\bar{\rho}2\pi(1-\cos r)}{\mu(\mathbb{S}^2)}} \\
\mathbb{E}[\hat{K}_{inhom}(r)] &= 2\pi(1-\cos r),
\end{aligned}$$

where $r \in [0, \pi]$, and $\bar{\rho} = \inf_{\mathbf{x}\in\mathbb{S}^2} \rho(\mathbf{x}) > 0$. Further by unbiasedness and ratio-unbiasedness of $\hat{F}_{inhom}(r)$ and $\hat{H}_{inhom}(r)$, respectively, we immediately have ratio-unbiasedness of $\hat{J}_{inhom}(r)$.

Proof. Proofs for the expectation of $\hat{F}_{\text{inhom}}$-, and $\hat{K}_{\text{inhom}}$-functions are found in van Lieshout [3] and Lawrence et al. [6], Møller and Rubak [7] respectively, whilst ratio-unbiasedness of the $\hat{H}_{\text{inhom}}$-function is also found in van Lieshout [3]. The proofs found in van Lieshout [3] are in $\mathbb{R}^n$ but can be extended easily to $\mathbb{S}^2$. Then for the expectation of $\hat{H}_{\text{inhom}}(r)$,

$$\begin{aligned}
&\mathbb{E}\left[\frac{1}{N_X(\mathbb{S}^2)}\sum_{\mathbf{x}\in X}\prod_{\mathbf{y}\in X\setminus\{\mathbf{x}\}}\left(1-\frac{\bar{\rho}\mathbb{1}[\mathbf{y}\in B_{\mathbb{S}^2}(\mathbf{x},r)]}{\rho(\mathbf{y})}\right)\right] \\
&\quad=\mathbb{E}\left[\sum_{\mathbf{x}\in X}\frac{1}{N_X(\mathbb{S}^2)}\prod_{\mathbf{y}\in X\setminus\{\mathbf{x}\}}\left(1-\frac{\bar{\rho}\mathbb{1}[\mathbf{y}\in B_{\mathbb{S}^2}(\mathbf{x},r)]}{\rho(\mathbf{y})}\right)\right] \\
&\quad=\mathbb{E}\left[\sum_{\mathbf{x}\in X}\frac{1}{|X|}\prod_{\mathbf{y}\in X\setminus\{\mathbf{x}\}}\left(1-\frac{\bar{\rho}\mathbb{1}[\mathbf{y}\in B_{\mathbb{S}^2}(\mathbf{x},r)]}{\rho(\mathbf{y})}\right)\right] \\
&\quad=\mathbb{E}\left[\sum_{\mathbf{x}\in X}\frac{1}{|X\setminus\{\mathbf{x}\}\cup\{\mathbf{x}\}|}\prod_{\mathbf{y}\in X\setminus\{\mathbf{x}\}}\left(1-\frac{\bar{\rho}\mathbb{1}[\mathbf{y}\in B_{\mathbb{S}^2}(\mathbf{x},r)]}{\rho(\mathbf{y})}\right)\right],
\end{aligned}$$

applying the Slivnyak-Mecke Theorem,

$$\begin{aligned}
&=\int_{\mathbb{S}^2}\mathbb{E}\left[\frac{1}{|X\cup\{\mathbf{x}\}|}\prod_{\mathbf{y}\in X}\left(1-\frac{\bar{\rho}\mathbb{1}[\mathbf{y}\in B_{\mathbb{S}^2}(\mathbf{x},r)]}{\rho(\mathbf{y})}\right)\right]\rho(\mathbf{x})\lambda_{\mathbb{S}^2}(d\mathbf{x}) \\
&=\int_{\mathbb{S}^2}\mathbb{E}\left[\frac{1}{N_X(\mathbb{S}^2)+1}\prod_{\mathbf{y}\in X}\left(1-\frac{\bar{\rho}\mathbb{1}[\mathbf{y}\in B_{\mathbb{S}^2}(\mathbf{x},r)]}{\rho(\mathbf{y})}\right)\right]\rho(\mathbf{x})\lambda_{\mathbb{S}^2}(d\mathbf{x}). \qquad (7)
\end{aligned}$$

We then take the expectation in the integrand and handle it separately, using the definition of a Poisson process being the independent distribution of a Poisson number of points.

$$\begin{aligned}
&\mathbb{E}\left[\frac{1}{N_X(\mathbb{S}^2)+1}\prod_{\mathbf{y}\in X}\left(1-\frac{\bar{\rho}\mathbb{1}[\mathbf{y}\in B_{\mathbb{S}^2}(\mathbf{x},r)]}{\rho(\mathbf{y})}\right)\right] \\
&\quad=\mathbb{E}\left[\frac{1}{N_X(\mathbb{S}^2)+1}\mathbb{E}\left[\prod_{\mathbf{y}\in X}\left(1-\frac{\bar{\rho}\mathbb{1}[\mathbf{y}\in B_{\mathbb{S}^2}(\mathbf{x},r)]}{\rho(\mathbf{y})}\right)\middle| N_X(\mathbb{S}^2)=n\right]\right] \\
&\quad=\mathbb{E}\left[\frac{1}{N_X(\mathbb{S}^2)+1}\mathbb{E}\left[\prod_{i=1}^{n}\left(1-\frac{\bar{\rho}\mathbb{1}[\mathbf{X}_i\in B_{\mathbb{S}^2}(\mathbf{x},r)]}{\rho(\mathbf{X}_i)}\right)\right]\right],
\end{aligned}$$

where $\mathbf{X}_i$ are independently distributed across $\mathbb{S}^2$ with density $\frac{\rho(\mathbf{x})}{\mu(\mathbb{S}^2)}$. Then taking the first

expectation,

$$\mathbb{E}\left[\prod_{i=1}^{n}\left(1-\frac{\bar{\rho}\mathbb{1}[\mathbf{X}_i \in B_{\mathbb{S}^2}(\mathbf{x},r)]}{\rho(\mathbf{X}_i)}\right)\right] = \overbrace{\int_{\mathbb{S}^2}\cdots\int_{\mathbb{S}^2}}^{n}\prod_{i=1}^{n}\left(1-\frac{\bar{\rho}\mathbb{1}[\mathbf{x}_i \in B_{\mathbb{S}^2}(\mathbf{x},r)]}{\rho(\mathbf{x}_i)}\right)\frac{\rho(\mathbf{x}_i)}{\mu(\mathbb{S}^2)}\lambda_{\mathbb{S}^2}(d\mathbf{x})_i$$

$$= \left(\int_{\mathbb{S}^2}\frac{\rho(\mathbf{y})}{\mu(\mathbb{S}^2)} - \bar{\rho}\mathbb{1}[\mathbf{y} \in B_{\mathbb{S}^2}(\mathbf{x},r)]d\mathbf{y}\right)^n$$

$$= \left(1-\frac{\bar{\rho}}{\mu(\mathbb{S}^2)}2\pi(1-\cos r)\right)^n$$

Returning to the expectation over $N_X(\mathbb{S}^2)$ we have,

$$\mathbb{E}\left[\frac{1}{N_X(\mathbb{S}^2)+1}\prod_{\mathbf{y}\in X}\left(1-\frac{\bar{\rho}\mathbb{1}[\mathbf{y} \in B_{\mathbb{S}^2}(\mathbf{x},r)]}{\rho(\mathbf{y})}\right)\right]$$

$$= \mathbb{E}\left[\frac{1}{N_X(\mathbb{S}^2)+1}\left(1-\frac{\bar{\rho}}{\mu(\mathbb{S}^2)}2\pi(1-\cos r)\right)^{N_X(\mathbb{S}^2)}\right],$$

define $A \equiv 1-\frac{\bar{\rho}}{\mu(\mathbb{S}^2)}2\pi(1-\cos r)$, then

$$= \mathbb{E}\left[\frac{1}{N_X(\mathbb{S}^2)+1}A^{N_X(\mathbb{S}^2)}\right],$$

$$= \sum_{n=0}^{\infty}\frac{A^n}{n+1}\frac{\lambda^n \mathrm{e}^{-\lambda}}{n!},$$

where $\lambda \equiv \mu(\mathbb{S}^2)$,

$$= \frac{\mathrm{e}^{-\lambda}}{A\lambda}\sum_{n=0}^{\infty}\frac{(A\lambda)^{n+1}}{(n+1)!}$$

$$= \frac{\mathrm{e}^{-\lambda}}{A\lambda}\sum_{n=1}^{\infty}\frac{(A\lambda)^{n}}{n!}$$

$$= \frac{\mathrm{e}^{-\lambda}}{A\lambda}\left(\sum_{n=0}^{\infty}\frac{(A\lambda)^{n}}{n!}-1\right)$$

$$= \frac{\mathrm{e}^{-\lambda}}{A\lambda}\left(\mathrm{e}^{A\lambda}-1\right)$$

$$= \frac{\mathrm{e}^{-\bar{\rho}2\pi(1-\cos r)}-\mathrm{e}^{-\mu(\mathbb{S}^2)}}{\mu(\mathbb{S}^2)-\bar{\rho}2\pi(1-\cos r)},$$

plugging this into Equation 7,

$$\int_{\mathbb{S}^2}\frac{\mathrm{e}^{-\bar{\rho}2\pi(1-\cos r)}-\mathrm{e}^{-\mu(\mathbb{S}^2)}}{\mu(\mathbb{S}^2)-\bar{\rho}2\pi(1-\cos r)}\rho(\mathbf{x})\lambda_{\mathbb{S}^2}(d\mathbf{x}) = \frac{\mathrm{e}^{-\bar{\rho}2\pi(1-\cos r)}-\mathrm{e}^{-\mu(\mathbb{S}^2)}}{1-\frac{\bar{\rho}2\pi(1-\cos r)}{\mu(\mathbb{S}^2)}},$$

and so,

$$\mathbb{E}[\hat{H}_{\text{inhom}}(r)] = 1 - \frac{\mathrm{e}^{-\bar{\rho}2\pi(1-\cos r)} - \mathrm{e}^{-\mu(\mathbb{S}^2)}}{1 - \frac{\bar{\rho}2\pi(1-\cos r)}{\mu(\mathbb{S}^2)}}.$$

□

The following corollary puts a bound on the bias of $\hat{H}_{\text{inhom}}(r)$ when we consider the scenario of CSR on $\mathbb{D}$ mapped on $\mathbb{S}^2$.

Corollary 2. *With the same assumptions as Theorem 3, let X be a spherical Poisson process on $\mathbb{S}^2$ with intensity function $\rho : \mathbb{S}^2 \mapsto \mathbb{R}_+$. Defining $\bar{\rho} = \inf_{\mathbf{x}\in\mathbb{S}^2} \rho(\mathbf{x})$, the bias of the estimator $\hat{H}_{inhom}(r)$ is bounded by*

$$|Bias(\hat{H}_{inhom}(r))| \leq \exp(-\mu(\mathbb{S}^2)) \leq \exp(-4\pi\bar{\rho}),$$

for all $r \in [0, \pi]$.

Proof.

$$\begin{aligned}
\text{Bias}(\hat{H}_{\text{inhom}}(r)) &= \left(1 - \frac{\mathrm{e}^{-\bar{\rho}2\pi(1-\cos r)} - \mathrm{e}^{-\mu(\mathbb{S}^2)}}{1 - \frac{\bar{\rho}2\pi(1-\cos r)}{\mu(\mathbb{S}^2)}}\right) - \left(1 - \mathrm{e}^{-\bar{\rho}2\pi(1-\cos r)}\right) \\
&= \frac{\exp(\mu(\mathbb{S}^2)) - \frac{\bar{\rho}2\pi(1-\cos r)}{\mu(\mathbb{S}^2)} \exp(-2\pi(1-\cos r)\bar{\rho})}{1 - \frac{\bar{\rho}2\pi(1-\cos r)}{\mu(\mathbb{S}^2)}} \\
&= \frac{\mu(\mathbb{S}^2)\exp(\mu(\mathbb{S}^2)) - \bar{\rho}2\pi(1-\cos r)\exp(-2\pi(1-\cos r)\bar{\rho})}{\mu(\mathbb{S}^2) - \bar{\rho}2\pi(1-\cos r)}
\end{aligned}$$

Taking the absolute value of the bias and the numerator is bounded above by,

$$\begin{aligned}
&|\mu(\mathbb{S}^2)\exp(\mu(\mathbb{S}^2)) - \bar{\rho}2\pi(1-\cos r)\exp(-2\pi(1-\cos r)\bar{\rho})| \\
&\qquad \leq \mu(\mathbb{S}^2)\exp(\mu(\mathbb{S}^2)) + \bar{\rho}2\pi(1-\cos r)\exp(-2\pi(1-\cos r)\bar{\rho}) \\
&\qquad \leq \mu(\mathbb{S}^2)\exp(\mu(\mathbb{S}^2)),
\end{aligned}$$

where the fist line follows from the triangle inequality. The denominator is bounded below,

$$\begin{aligned}
|\mu(\mathbb{S}^2) - \bar{\rho}2\pi(1-\cos r)| &\geq \mu(\mathbb{S}^2) + \bar{\rho}2\pi(1-\cos r) \\
&\geq \mu(\mathbb{S}^2),
\end{aligned}$$

where again the first line follows from the triangle inequality, since $|a-b+b| \leq |a-b|+|b| \Rightarrow |a| - |b| \leq |a-b|$, for $a, b \in \mathbb{R}$. Hence the absolute of the bias is bounded by,

$$|\text{Bias}(\hat{H}_{\text{inhom}}(r))| \leq \exp(\mu(\mathbb{S}^2)).$$

The final inequality follows by noting that $\mu(\mathbb{S}^2) = \int_{\mathbb{S}^2} \rho(\mathbf{x})\lambda_{\mathbb{S}^2}(d\mathbf{x}) \geq \int_{\mathbb{S}^2} \bar{\rho}\lambda_{\mathbb{S}^2}(d\mathbf{x}) = 4\pi\bar{\rho}$. $\square$

S4. Variance of functional summary statistics

In this section we derive the variance of the functional summary statistics. Throughout this section we will frequently refer to the area of a spherical cap, at any point $\mathbf{o} \in \mathbb{S}^2$ with geodesic distance r by $B_{\mathbb{S}^2}(\mathbf{o}, r)$. We will also make use of the extended Campbell-Mecke Theorem [5] throughout,

Theorem S3. *Let X be a point process on $\mathbb{S}^2$ and h be any non-negative, measurable function such that $h : (\times_{i=1}^n \mathbb{S}^2) \times N_{lf} \mapsto \mathbb{R}$. Then,*

$$\mathbb{E} \sum_{\mathbf{x}_1,\ldots,\mathbf{x}_n \in X}^{\neq} h(\mathbf{x}_1, \ldots, \mathbf{x}_n, X \setminus \{\mathbf{x}_1, \ldots, \mathbf{x}_n\}) \\ = \int_{\mathbb{S}^2} \cdots \int_{\mathbb{S}^2} \mathbb{E}[h(\mathbf{x}_1, \ldots, \mathbf{x}_n, X^!_{\mathbf{x}_1,\ldots,\mathbf{x}_n})]\alpha(\lambda_{\mathbb{S}^2}(d\mathbf{x})_1, \ldots, \lambda_{\mathbb{S}^2}(d\mathbf{x})_n)$$

and if the intensity function of order n exists then,

$$\mathbb{E} \sum_{\mathbf{x}_1,\ldots,\mathbf{x}_n \in X}^{\neq} h(\mathbf{x}_1, \ldots, \mathbf{x}_n, X \setminus \{\mathbf{x}_1, \ldots, \mathbf{x}_n\}) \\ = \int_{\mathbb{S}^2} \cdots \int_{\mathbb{S}^2} \mathbb{E}[h(\mathbf{x}_1, \ldots, \mathbf{x}_n, X^!_{\mathbf{x}_1,\ldots,\mathbf{x}_n})]\rho^{(n)}(\mathbf{x}_1, \ldots, \mathbf{x}_n)\lambda_{\mathbb{S}^2}(\lambda_{\mathbb{S}^2}(d\mathbf{x})_1) \cdots \lambda_{\mathbb{S}^2}(\lambda_{\mathbb{S}^2}(d\mathbf{x})_n),$$

where $X^!_{\mathbf{x}_1,\ldots,\mathbf{x}_n}$ is the nth-order reduced Palm process of X.

Proof. See Møller and Waagepetersen [5, Appendix C, pp. 248-249]. □

Again, as in the case for when $n = 1$, for any order n the reduced Palm process of order n, $X^!_{\mathbf{x}_1,\ldots,\mathbf{x}_n}$, for a Poisson process, X, is again the same Poisson process. Hence we get the extended Slivnyak-Mecke Theorem [5, Theorem 3.3, p. 22],

$$\mathbb{E} \sum_{\mathbf{x}_1,\ldots,\mathbf{x}_n \in X}^{\neq} h(\mathbf{x}_1, \ldots, \mathbf{x}_n, X \setminus \{\mathbf{x}_1, \ldots, \mathbf{x}_n\}) \\ = \int_{\mathbb{S}^2} \cdots \int_{\mathbb{S}^2} \mathbb{E}[h(\mathbf{x}_1, \ldots, \mathbf{x}_n, X)] \prod_{i=1}^n \rho(\mathbf{x}_i)\lambda_{\mathbb{S}^2}(\lambda_{\mathbb{S}^2}(d\mathbf{x})_1) \cdots \lambda_{\mathbb{S}^2}(\lambda_{\mathbb{S}^2}(d\mathbf{x})_n), \tag{8}$$

To derive the variance of $\hat{K}_{\text{inhom}}(r)$, before which we require the following lemma,

Lemma S2. *Let X be a finite set and define X^n as the Cartesian product of X n times, i.e. $X^n = X \times \cdots \times X$, and the following sets,*

$$Y = \{(\mathbf{x}_1, \mathbf{x}_2, \mathbf{x}_3, \mathbf{x}_4)^T \in X^4 : \mathbf{x}_1 \in X, \mathbf{x}_2 \in X \setminus \{\mathbf{x}_1\}, \mathbf{x}_3 \in X, \mathbf{x}_4 \in X \setminus \{\mathbf{x}_3\}\}$$
$$Y_1 = \{(\mathbf{x}_1, \mathbf{x}_2, \mathbf{x}_3, \mathbf{x}_4)^T \in X^4 : \mathbf{x}_1 \in X, \mathbf{x}_2 \in X \setminus \{\mathbf{x}_1\}, \mathbf{x}_3 \in X \setminus \{\mathbf{x}_1, \mathbf{x}_2\}, \mathbf{x}_4 \in X \setminus \{\mathbf{x}_1, \mathbf{x}_2, \mathbf{x}_3\}\}$$
$$Y_2 = \{(\mathbf{x}_1, \mathbf{x}_2, \mathbf{x}_3, \mathbf{x}_4)^T \in X^4 : \mathbf{x}_1 \in X, \mathbf{x}_2 \in X \setminus \{\mathbf{x}_1\}, \mathbf{x}_3 \in \{\mathbf{x}_1, \mathbf{x}_2\}, \mathbf{x}_4 \in \{\mathbf{x}_1, \mathbf{x}_2\} \setminus \{\mathbf{x}_3\}\}$$
$$Y_3 = \{(\mathbf{x}_1, \mathbf{x}_2, \mathbf{x}_3, \mathbf{x}_4)^T \in X^4 : \mathbf{x}_1 \in X, \mathbf{x}_2 \in X \setminus \{\mathbf{x}_1\}, \mathbf{x}_3 \in X \setminus \{\mathbf{x}_1, \mathbf{x}_2\}, \mathbf{x}_4 \in \{\mathbf{x}_1, \mathbf{x}_2\} \setminus \{\mathbf{x}_3\}\}$$
$$Y_4 = \{(\mathbf{x}_1, \mathbf{x}_2, \mathbf{x}_3, \mathbf{x}_4)^T \in X^4 : \mathbf{x}_1 \in X, \mathbf{x}_2 \in X \setminus \{\mathbf{x}_1\}, \mathbf{x}_3 \in \{\mathbf{x}_1, \mathbf{x}_2\}, \mathbf{x}_4 \in X \setminus \{\mathbf{x}_1, \mathbf{x}_2, \mathbf{x}_3\}\},$$

then $Y = \cup_{i=1,\ldots,4} Y_i$ and $Y_i, i = 1, \ldots, 4$ are pairwise disjoint.

Proof. Pairwise disjointness of the sets $Y_i, i = 1 \ldots 4$ follows from the definitions of the sets. To prove equality we will show the following,

$$\mathbf{r} \in Y \Rightarrow \mathbf{r} \in \cup_{i=1,\ldots,4} Y_i, \quad \text{and} \tag{9}$$
$$\mathbf{r} \in \cup_{i=1,\ldots,4} Y_i \Rightarrow \mathbf{r} \in Y \tag{10}$$

Statement 10, holds by considering each set Y_i in turn. In particular it is clear by the definitions of the sets that for each $i = 1, \ldots, 4, Y_i \subseteq Y$ and so Statement 10 holds.

To show Statement 9 let $\mathbf{x} \in Y$ and fix $\mathbf{x}_1 \in X$ and $\mathbf{x}_2 \in X \setminus \{\mathbf{x}_1\}$. Then there are two possibilities for $\mathbf{x}_3$, either $\mathbf{x}_3 \in X \setminus \{\mathbf{x}_1, \mathbf{x}_2\}$ or $\mathbf{x}_3 \in \{\mathbf{x}_1, \mathbf{x}_2\}$. If the former holds then $\mathbf{x}_4$ can either be in $X \setminus \{\mathbf{x}_1, \mathbf{x}_2, \mathbf{x}_3\}$ or $\{\mathbf{x}_1, \mathbf{x}_2\} \setminus \{\mathbf{x}_3\}$. If the first holds then $\mathbf{x} \in Y_1$ and if the second holds then $\mathbf{x} \in Y_3$. Considering all possible combinations proves Statement 9. Hence it follows that $Y = \cup_{i=1,\ldots,4} Y_i$. □

We now proceed with the proof for the variance of estimates of the inhomogeneous functional summary statistics.

Theorem 4. *Let X be a spherical Poisson process on $\mathbb{S}^2$ with known intensity function $\rho : \mathbb{S}^2 \mapsto \mathbb{R}_+$, such that $\bar{\rho} = \inf_{\mathbf{x} \in \mathbb{S}^2} \rho(\mathbf{x}) > 0$. Then the estimators $\hat{K}_{\mathrm{inhom}}(r)$, $\hat{F}_{\mathrm{inhom}}(r)$, and $\hat{H}_{\mathrm{inhom}}(r)$ have variance,*

$$\mathrm{Var}(\hat{K}_{\mathrm{inhom}}(r)) = \frac{1}{8\pi^2} \int_{\mathbb{S}^2} \int_{\mathbb{S}^2} \frac{\mathbb{1}[d(\mathbf{x}, \mathbf{y}) \leq r]}{\rho(\mathbf{x})\rho(\mathbf{y})} \lambda_{\mathbb{S}^2}(d\mathbf{x}) \lambda_{\mathbb{S}^2}(d\mathbf{y}) + (1 - \cos r)^2 \int_{\mathbb{S}^2} \frac{1}{\rho(\mathbf{x})} \lambda_{\mathbb{S}^2}(d\mathbf{x}),$$

$$\mathrm{Var}(\hat{F}_{\mathrm{inhom}}(r)) = \frac{\exp\left(-2\bar{\rho}\lambda_{\mathbb{S}^2}(B_{\mathbb{S}^2}(\mathbf{o},r))\right)}{|P|^2} \sum_{\mathbf{p}\in P}\sum_{\mathbf{p}'\in P} \exp\left(\int_{B_{\mathbb{S}^2}(\mathbf{p},r)\cap B_{\mathbb{S}^2}(\mathbf{p}',r)} \frac{\bar{\rho}^2}{\rho(\mathbf{x})}\lambda_{\mathbb{S}^2}(d\mathbf{x})\right) - \exp\left(-2\bar{\rho}\lambda_{\mathbb{S}^2}(B_{\mathbb{S}^2}(\mathbf{o},r))\right),$$

$$\begin{aligned}
&\mathrm{Var}(\hat{H}_{\mathrm{inhom}}(r)) \\
&= \frac{1}{\mu^2(\mathbb{S}^2)}\int_{\mathbb{S}^2}\int_{\mathbb{S}^2} \left(\rho(\mathbf{x}) - \bar{\rho}\mathbb{1}[\mathbf{x}\in B_{\mathbb{S}^2}(\mathbf{y},r)]\right)\left(\rho(\mathbf{y}) - \bar{\rho}\mathbb{1}[\mathbf{y}\in B_{\mathbb{S}^2}(\mathbf{x},r)]\right) \\
&\quad \frac{\mathrm{e}^{-\mu(\mathbb{S}^2)}}{A_1^2(\mathbf{x},\mathbf{y})}\left(\mathrm{e}^{\mu(\mathbb{S}^2)A_1(\mathbf{x},\mathbf{y})} - 1 - \mathrm{Ei}(\mu(\mathbb{S}^2)\mathrm{A}_1(\mathbf{x},\mathbf{y})) + \gamma + \log(\mu(\mathbb{S}^2)\mathrm{A}_1(\mathbf{x},\mathbf{y}))\right)\lambda_{\mathbb{S}^2}(d\mathbf{x})\lambda_{\mathbb{S}^2}(d\mathbf{y}) \\
&\quad + \frac{1}{\mu(\mathbb{S}^2)}\int_{\mathbb{S}^2}\frac{\mathrm{e}^{-\mu(\mathbb{S}^2)}}{A_2(\mathbf{x})}\left(\gamma + \log(\mu(\mathbb{S}^2)A_2(\mathbf{x})) - \mathrm{Ei}(\mu(\mathbb{S}^2)\mathrm{A}_2(\mathbf{x}))\right)\rho(\mathbf{y})\lambda_{\mathbb{S}^2}(d\mathbf{y}) \\
&\quad - \frac{\mathrm{e}^{-2\mu(\mathbb{S}^2)}}{\left(1 - \frac{\bar{\rho}}{\mu(\mathbb{S}^2)}2\pi(1-\cos r)\right)^2}\left(\mathrm{e}^{\mu(\mathbb{S}^2)\left(1-\frac{\bar{\rho}}{\mu(\mathbb{S}^2)}2\pi(1-\cos r)\right)} - 1\right)^2
\end{aligned}$$

where,

$$\begin{aligned}
A_1(\mathbf{x},\mathbf{y}) &= 1 - \frac{2\bar{\rho}}{\mu(\mathbb{S}^2)}2\pi(1-\cos r) + \frac{\bar{\rho}^2}{\mu(\mathbb{S}^2)}\int_{B_{\mathbb{S}^2}(\mathbf{x},r)\cap B_{\mathbb{S}^2}(\mathbf{y},r)}\frac{1}{\rho(\mathbf{z})}d\mathbf{z} \\
A_2(\mathbf{x}) &= 1 - \frac{2\bar{\rho}}{\mu(\mathbb{S}^2)}2\pi(1-\cos r) + \frac{\bar{\rho}^2}{\mu(\mathbb{S}^2)}\int_{B_{\mathbb{S}^2}(\mathbf{x},r)}\frac{1}{\rho(\mathbf{y})}\lambda_{\mathbb{S}^2}(d\mathbf{y}) \\
\mathrm{Ei}(\mathrm{x}) &= -\int_{-x}^{\infty}\frac{\mathrm{e}^{-t}}{t}dt
\end{aligned}$$

and $\mathrm{Ei}(\mathrm{x})$ *is the exponential integral and* $r \in [0,\pi]$.

The proof is spread over three parts, one for each functional summary statistic.

Proof. Variance of $\hat{K}_{inhom}(r)$

We expand the variance as $\mathrm{Var}(X) = \mathbb{E}[X^2] - \mathbb{E}^2[X]$,

$$\begin{aligned}
\mathrm{Var}(\tilde{K}_{\mathrm{inhom}}(r)) &= \mathrm{Var}\left(\frac{1}{4\pi}\sum_{\mathbf{x}\in X}\sum_{\mathbf{y}\in X\setminus\{\mathbf{x}\}}\frac{\mathbb{1}[d(\mathbf{x},\mathbf{y})\le r]}{\rho(\mathbf{x})\rho(\mathbf{y})}\right) \\
&= \frac{1}{16\pi^2}\Bigg[\underbrace{\mathbb{E}\left(\sum_{\mathbf{x}\in X}\sum_{\mathbf{y}\in X\setminus\{\mathbf{x}\}}\frac{\mathbb{1}[d(\mathbf{x},\mathbf{y})\le r]}{\rho(\mathbf{x})\rho(\mathbf{y})}\right)^2}_{(1)} - \underbrace{\mathbb{E}^2\sum_{\mathbf{x}\in X}\sum_{\mathbf{y}\in X\setminus\{\mathbf{x}\}}\frac{\mathbb{1}(d(\mathbf{x},\mathbf{y})\le r)}{\rho(\mathbf{x})\rho(\mathbf{y})}}_{(2)}\Bigg],
\end{aligned} \tag{11}$$

We deal with each of the terms individually. First consider term (2) of the previous equation, this is simply the inhomogeneous K-function for a Poisson process,

$$\begin{aligned}\frac{1}{16\pi^2}\mathbb{E}^2\sum_{\mathbf{x}\in X}\sum_{\mathbf{y}\in X\setminus\{\mathbf{x}\}}\frac{\mathbb{1}(d(\mathbf{x},\mathbf{y})\le r)}{\rho(\mathbf{x})\rho(\mathbf{y})} &= \mathbb{E}^2\frac{1}{4\pi}\sum_{\mathbf{x}\in X}\sum_{\mathbf{y}\in X\setminus\{\mathbf{x}\}}\frac{\mathbb{1}(d(\mathbf{x},\mathbf{y})\le r)}{\rho(\mathbf{x})\rho(\mathbf{y})}\\ &= K^2_{\text{inhom}}(r)\\ &= 4\pi^2(1-\cos r)^2,\end{aligned}$$

where the penultimate equality follows from the definition of K_{inhom} (taking the arbitrary area B to be $\mathbb{S}^2$) and the final equality follows from Proposition 3. To handle term (1) we first expand the square,

$$\begin{aligned}&\frac{1}{16\pi^2}\mathbb{E}\left(\sum_{\mathbf{x}\in X}\sum_{\mathbf{y}\in X\setminus\{\mathbf{x}\}}\frac{\mathbb{1}[d(\mathbf{x},\mathbf{y})\le r]}{\rho(\mathbf{x})\rho(\mathbf{y})}\right)^2\\ &\quad= \frac{1}{16\pi^2}\mathbb{E}\sum_{\mathbf{x}\in X}\sum_{\mathbf{y}\in X\setminus\{\mathbf{x}\}}\frac{\mathbb{1}[d(\mathbf{x},\mathbf{y})\le r]}{\rho(\mathbf{x})\rho(\mathbf{y})}\sum_{\mathbf{x}'\in X}\sum_{\mathbf{y}'\in X\setminus\{\mathbf{x}'\}}\frac{\mathbb{1}[d(\mathbf{x}',\mathbf{y}')\le r]}{\rho(\mathbf{x}')\rho(\mathbf{y}')}\\ &\quad= \frac{1}{16\pi^2}\mathbb{E}\sum_{\mathbf{x}\in X}\sum_{\mathbf{y}\in X\setminus\{\mathbf{x}\}}\sum_{\mathbf{x}'\in X}\sum_{\mathbf{y}'\in X\setminus\{\mathbf{x}'\}}\frac{\mathbb{1}[d(\mathbf{x},\mathbf{y})\le r]\mathbb{1}[d(\mathbf{x}',\mathbf{y}')\le r]}{\rho(\mathbf{x})\rho(\mathbf{y})\rho(\mathbf{x}')\rho(\mathbf{y}')}\end{aligned}$$

Let us define the summand as,

$$f_r(\mathbf{x},\mathbf{y},\mathbf{x}',\mathbf{y}') = \frac{\mathbb{1}[d(\mathbf{x},\mathbf{y})\le r]\mathbb{1}[d(\mathbf{x}',\mathbf{y}')\le r]}{\rho(\mathbf{x})\rho(\mathbf{y})\rho(\mathbf{x}')\rho(\mathbf{y}')},$$

and then by Lemma S2 we can divide the sum in the expectation into 4 terms,

$$\begin{aligned}&\underbrace{\frac{1}{16\pi^2}\mathbb{E}\sum_{\mathbf{x}\in X}\sum_{\mathbf{y}\in X\setminus\{\mathbf{x}\}}\sum_{\mathbf{x}'\in X\setminus\{\mathbf{x},\mathbf{y}\}}\sum_{\mathbf{y}'\in X\setminus\{\mathbf{x}',\mathbf{x},\mathbf{y}\}} f_r(\mathbf{x},\mathbf{y},\mathbf{x}',\mathbf{y}')}_{(a)}\\ &\quad+\underbrace{\frac{1}{16\pi^2}\mathbb{E}\sum_{\mathbf{x}\in X}\sum_{\mathbf{y}\in X\setminus\{\mathbf{x}\}}\sum_{\mathbf{x}'\in\{\mathbf{x},\mathbf{y}\}}\sum_{\mathbf{y}'\in\{\mathbf{x},\mathbf{y}\}\setminus\{\mathbf{x}'\}} f_r(\mathbf{x},\mathbf{y},\mathbf{x}',\mathbf{y}')}_{(b)}\\ &\quad+\underbrace{\frac{1}{16\pi^2}\mathbb{E}\sum_{\mathbf{x}\in X}\sum_{\mathbf{y}\in X\setminus\{\mathbf{x}\}}\sum_{\mathbf{x}'\in X\setminus\{\mathbf{x},\mathbf{y}\}}\sum_{\mathbf{y}'\in\{\mathbf{x},\mathbf{y}\}\setminus\{\mathbf{x}'\}} f_r(\mathbf{x},\mathbf{y},\mathbf{x}',\mathbf{y}')}_{(c)}\end{aligned}$$

$$
+\underbrace{\frac{1}{16\pi^2}\mathbb{E}\sum_{\mathbf{x}\in X}\sum_{\mathbf{y}\in X\setminus\{\mathbf{x}\}}\sum_{\mathbf{x}'\in\{\mathbf{x},\mathbf{y}\}}\sum_{\mathbf{y}'\in X\setminus\{\mathbf{x},\mathbf{y},\mathbf{x}'\}} f_r(\mathbf{x},\mathbf{y},\mathbf{x}',\mathbf{y}')}_{(d)}\,.
$$

We handle these terms independently. For term (a) we can directly apply the extended Slivnyak-Mecke Theorem given by Equation 8,

$$
\begin{aligned}
&\frac{1}{16\pi^2}\mathbb{E}\sum_{\mathbf{x}\in X}\sum_{\mathbf{y}\in X\setminus\{\mathbf{x}\}}\sum_{\mathbf{x}'\in X\setminus\{\mathbf{x},\mathbf{y}\}}\sum_{\mathbf{y}'\in X\setminus\{\mathbf{x}',\mathbf{x},\mathbf{y}\}} f_r(\mathbf{x},\mathbf{y},\mathbf{x}',\mathbf{y}') \\
&\qquad= \frac{1}{16\pi^2}\int_{\mathbb{S}^2}\cdots\int_{\mathbb{S}^2} f_r(\mathbf{x},\mathbf{y},\mathbf{x}',\mathbf{y}')\rho(\mathbf{x})\rho(\mathbf{y})\rho(\mathbf{x}')\rho(\mathbf{y}')\lambda_{\mathbb{S}^2}(d\mathbf{x})\lambda_{\mathbb{S}^2}(d\mathbf{y})\lambda_{\mathbb{S}^2}(d\mathbf{x})'\lambda_{\mathbb{S}^2}(d\mathbf{y})' \\
&\qquad= \frac{1}{16\pi^2}\int_{\mathbb{S}^2}\cdots\int_{\mathbb{S}^2} \mathbb{1}[d(\mathbf{x},\mathbf{y})\le r]\mathbb{1}[d(\mathbf{x}',\mathbf{y}')\le r]\lambda_{\mathbb{S}^2}(d\mathbf{x})\lambda_{\mathbb{S}^2}(d\mathbf{y})\lambda_{\mathbb{S}^2}(d\mathbf{x})'\lambda_{\mathbb{S}^2}(d\mathbf{y})' \\
&\qquad= \frac{1}{16\pi^2}\left(\int_{\mathbb{S}^2}\int_{\mathbb{S}^2}\mathbb{1}[d(\mathbf{x},\mathbf{y})\le r]\lambda_{\mathbb{S}^2}(d\mathbf{x})\lambda_{\mathbb{S}^2}(d\mathbf{y})\right)^2 \\
&\qquad= \frac{1}{16\pi^2}\left(\int_{\mathbb{S}^2}\lambda_{\mathbb{S}^2}(B_{\mathbb{S}^2}(\mathbf{o},r))\lambda_{\mathbb{S}^2}(d\mathbf{y})\right)^2 \\
&\qquad= \frac{1}{16\pi^2}\left(4\pi\lambda_{\mathbb{S}^2}(B_{\mathbb{S}^2}(\mathbf{o},r))\right)^2 \\
&\qquad= \lambda_{\mathbb{S}^2}(B_{\mathbb{S}^2}(\mathbf{o},r))^2,
\end{aligned}
$$

where the penultimate equality follows since the area of the spherical cap is constant for a fixed geodesic radius for any centre, $\mathbf{0}$ indicates any arbitrary point in $\mathbb{S}^2$. Term (b) can be handled in a similar manner as term (a),

$$
\begin{aligned}
&\frac{1}{16\pi^2}\mathbb{E}\sum_{\mathbf{x}\in X}\sum_{\mathbf{y}\in X\setminus\{\mathbf{x}\}}\sum_{\mathbf{x}'\in\{\mathbf{x},\mathbf{y}\}}\sum_{\mathbf{y}'\in\{\mathbf{x},\mathbf{y}\}\setminus\{\mathbf{x}'\}} f_r(\mathbf{x},\mathbf{y},\mathbf{x}',\mathbf{y}') \\
&\qquad= \frac{1}{16\pi^2}\mathbb{E}\sum_{\mathbf{x}\in X}\sum_{\mathbf{y}\in X\setminus\{\mathbf{x}\}} f_r(\mathbf{x},\mathbf{y},\mathbf{x},\mathbf{y}) + f_r(\mathbf{x},\mathbf{y},\mathbf{y},\mathbf{x}) \\
&\qquad= \frac{1}{16\pi^2}\mathbb{E}\sum_{\mathbf{x}\in X}\sum_{\mathbf{y}\in X\setminus\{\mathbf{x}\}} \frac{\mathbb{1}[d(\mathbf{x},\mathbf{y})\le r]\mathbb{1}[d(\mathbf{x},\mathbf{y})\le r]}{\rho(\mathbf{x})\rho(\mathbf{y})\rho(\mathbf{x})\rho(\mathbf{y})} \\
&\qquad\qquad + \frac{\mathbb{1}[d(\mathbf{x},\mathbf{y})\le r]\mathbb{1}[d(\mathbf{y},\mathbf{x})\le r]}{\rho(\mathbf{x})\rho(\mathbf{y})\rho(\mathbf{y})\rho(\mathbf{x})} \\
&\qquad= \frac{1}{8\pi^2}\mathbb{E}\sum_{\mathbf{x}\in X}\sum_{\mathbf{y}\in X\setminus\{\mathbf{x}\}} \frac{\mathbb{1}[d(\mathbf{x},\mathbf{y})\le r]}{\rho^2(\mathbf{x})\rho^2(\mathbf{y})}
\end{aligned}
$$

Hence by the extended Slivnyak-Mecke Theorem again we have,

$$\frac{1}{8\pi^2}\mathbb{E}\sum_{\mathbf{x}\in X}\sum_{\mathbf{y}\in X\setminus\{\mathbf{x}\}}\frac{\mathbb{1}[d(\mathbf{x},\mathbf{y})\leq r]}{\rho^2(\mathbf{x})\rho^2(\mathbf{y})} = \frac{1}{8\pi^2}\int_{\mathbb{S}^2}\int_{\mathbb{S}^2}\frac{\mathbb{1}[d(\mathbf{x},\mathbf{y})\leq r]}{\rho^2(\mathbf{x})\rho^2(\mathbf{y})}\rho(\mathbf{x})\rho(\mathbf{y})\lambda_{\mathbb{S}^2}(d\mathbf{x})\lambda_{\mathbb{S}^2}(d\mathbf{y})$$
$$= \frac{1}{8\pi^2}\int_{\mathbb{S}^2}\int_{\mathbb{S}^2}\frac{\mathbb{1}[d(\mathbf{x},\mathbf{y})\leq r]}{\rho(\mathbf{x})\rho(\mathbf{y})}\lambda_{\mathbb{S}^2}(d\mathbf{x})\lambda_{\mathbb{S}^2}(d\mathbf{y})$$

We now consider term (c),

$$\frac{1}{16\pi^2}\mathbb{E}\sum_{\mathbf{x}\in X}\sum_{\mathbf{y}\in X\setminus\{\mathbf{x}\}}\sum_{\mathbf{x}'\in X\setminus\{\mathbf{x},\mathbf{y}\}}\sum_{\mathbf{y}'\in\{\mathbf{x},\mathbf{y}\}\setminus\{\mathbf{x}'\}} f(\mathbf{x},\mathbf{y},\mathbf{x}',\mathbf{y}')$$
$$= \frac{1}{16\pi^2}\mathbb{E}\sum_{\mathbf{x}\in X}\sum_{\mathbf{y}\in X\setminus\{\mathbf{x}\}}\sum_{\mathbf{x}'\in X\setminus\{\mathbf{x},\mathbf{y}\}}\underbrace{f_r(\mathbf{x},\mathbf{y},\mathbf{x}',\mathbf{x})}_{(I)}+\underbrace{f_r(\mathbf{x},\mathbf{y},\mathbf{x}',\mathbf{y})}_{(II)}.$$

We can handle these terms independently.

$$\frac{1}{16\pi^2}\mathbb{E}\sum_{\mathbf{x}\in X}\sum_{\mathbf{y}\in X\setminus\{\mathbf{x}\}}\sum_{\mathbf{x}'\in X\setminus\{\mathbf{x},\mathbf{y}\}} f_r(\mathbf{x},\mathbf{y},\mathbf{x}',\mathbf{x})$$
$$= \frac{1}{16\pi^2}\mathbb{E}\sum_{\mathbf{x}\in X}\sum_{\mathbf{y}\in X\setminus\{\mathbf{x}\}}\sum_{\mathbf{x}'\in X\setminus\{\mathbf{x},\mathbf{y}\}}\frac{\mathbb{1}[d(\mathbf{x},\mathbf{y})\leq r]\mathbb{1}[d(\mathbf{x}',\mathbf{x})\leq r]}{\rho(\mathbf{x})\rho(\mathbf{y})\rho(\mathbf{x}')\rho(\mathbf{x})}$$
$$= \frac{1}{16\pi^2}\int_{\mathbb{S}^2}\int_{\mathbb{S}^2}\int_{\mathbb{S}^2}\frac{\mathbb{1}[d(\mathbf{x},\mathbf{y})\leq r]\mathbb{1}[d(\mathbf{x}',\mathbf{x})\leq r]}{\rho^2(\mathbf{x})\rho(\mathbf{y})\rho(\mathbf{x}')}\rho(\mathbf{x})\rho(\mathbf{y})\rho(\mathbf{x}')\lambda_{\mathbb{S}^2}(d\mathbf{x})\lambda_{\mathbb{S}^2}(d\mathbf{y})\lambda_{\mathbb{S}^2}(d\mathbf{x})'$$
$$= \frac{1}{16\pi^2}\int_{\mathbb{S}^2}\int_{\mathbb{S}^2}\int_{\mathbb{S}^2}\frac{\mathbb{1}[d(\mathbf{x},\mathbf{y})\leq r]\mathbb{1}[d(\mathbf{x}',\mathbf{x})\leq r]}{\rho(\mathbf{x})}\lambda_{\mathbb{S}^2}(d\mathbf{x})\lambda_{\mathbb{S}^2}(d\mathbf{y})\lambda_{\mathbb{S}^2}(d\mathbf{x})'$$
$$= \frac{1}{16\pi^2}\int_{\mathbb{S}^2}\int_{\mathbb{S}^2}\frac{\mathbb{1}[d(\mathbf{x},\mathbf{y})\leq r]}{\rho(\mathbf{x})}\left(\int_{\mathbb{S}^2}\mathbb{1}[d(\mathbf{x}',\mathbf{x})\leq r]\lambda_{\mathbb{S}^2}(d\mathbf{x})'\right)\lambda_{\mathbb{S}^2}(d\mathbf{x})\lambda_{\mathbb{S}^2}(d\mathbf{y})$$
$$= \frac{\lambda_{\mathbb{S}^2}(B_{\mathbb{S}^2}(\mathbf{o},r))}{16\pi^2}\int_{\mathbb{S}^2}\frac{1}{\rho(\mathbf{x})}\left(\int_{\mathbb{S}^2}\mathbb{1}[d(\mathbf{x},\mathbf{y})\leq r]\lambda_{\mathbb{S}^2}(d\mathbf{y})\right)\lambda_{\mathbb{S}^2}(d\mathbf{x})$$
$$= \frac{\lambda_{\mathbb{S}^2}(B_{\mathbb{S}^2}(\mathbf{o},r))^2}{16\pi^2}\int_{\mathbb{S}^2}\frac{1}{\rho(\mathbf{x})}\lambda_{\mathbb{S}^2}(d\mathbf{x}),$$

where the second equality follows by the Slivnyak-Mecke Theorem. By an identical argument term II is given by,

$$\frac{1}{16\pi^2}\mathbb{E}\sum_{\mathbf{x}\in X}\sum_{\mathbf{y}\in X\setminus\{\mathbf{x}\}}\sum_{\mathbf{x}'\in X\setminus\{\mathbf{x},\mathbf{y}\}} f_r(\mathbf{x},\mathbf{y},\mathbf{x}',\mathbf{y}) = \frac{\lambda_{\mathbb{S}^2}(B_{\mathbb{S}^2}(\mathbf{o},r))^2}{16\pi^2}\int_{\mathbb{S}^2}\frac{1}{\rho(\mathbf{y})}\lambda_{\mathbb{S}^2}(d\mathbf{y})$$

Term (d) can be handled in an identical manner as term (c). To see this consider the summation over $\mathbf{y}'$ in term (d). Since $\mathbf{x}'\in\{\mathbf{x},\mathbf{y}\}$ this means that the set $X\setminus\{\mathbf{x},\mathbf{y},\mathbf{x}'\}$ is

identical to $X \setminus \{\mathbf{x}, \mathbf{y}\}$ and so the summations over $\mathbf{x}'$ and $\mathbf{y}'$ can be interchanged. Therefore,

$$\frac{1}{16\pi^2}\mathbb{E}\sum_{\mathbf{x}\in X}\sum_{\mathbf{y}\in X\setminus\{\mathbf{x}\}}\sum_{\mathbf{x}'\in\{\mathbf{x},\mathbf{y}\}}\sum_{\mathbf{y}'\in X\setminus\{\mathbf{x},\mathbf{y},\mathbf{x}'\}} f_r(\mathbf{x},\mathbf{y},\mathbf{x}',\mathbf{y}') = \frac{2\lambda_{\mathbb{S}^2}(B_{\mathbb{S}^2}(\mathbf{o},r))^2}{16\pi^2}\int_{\mathbb{S}^2}\frac{1}{\rho(\mathbf{x})}\lambda_{\mathbb{S}^2}(d\mathbf{x}).$$

Further we note that the area of spherical cap is given by $\lambda_{\mathbb{S}^2}(B_{\mathbb{S}^2}(\mathbf{o},r)) = 2\pi(1-\cos r)$. Thus collecting all the terms gives the form of the variance,

$$\mathrm{Var}(\hat{K}_{\mathrm{inhom}}(r)) = \frac{1}{8\pi^2}\int_{\mathbb{S}^2}\int_{\mathbb{S}^2}\frac{\mathbb{1}[d(\mathbf{x},\mathbf{y})\le r]}{\rho(\mathbf{x})\rho(\mathbf{y})}\lambda_{\mathbb{S}^2}(d\mathbf{x})\lambda_{\mathbb{S}^2}(d\mathbf{y}) + (1-\cos r)^2\int_{\mathbb{S}^2}\frac{1}{\rho(\mathbf{x})}\lambda_{\mathbb{S}^2}(d\mathbf{x}). \tag{12}$$

□

Proof. Variance of $\hat{F}_{inhom}(r)$

Restating the estimator for $F_{\mathrm{inhom}(r)}$,

$$\hat{F}_{\mathrm{inhom}}(r) = 1 - \frac{\sum_{\mathbf{p}\in P}\prod_{\mathbf{x}\in X\cap B_{\mathbb{S}^2}(\mathbf{p},r)}\left(1-\frac{\bar{\rho}}{\rho(\mathbf{x})}\right)}{|P|}.$$

Taking the variance using $\mathrm{Var}(X) = \mathbb{E}[X^2] - \mathbb{E}^2[X]$,

$$\begin{aligned}\mathrm{Var}(\hat{F}_{\mathrm{inhom}}(r)) &= \frac{1}{|P|^2}\mathrm{Var}\sum_{\mathbf{p}\in P}\prod_{\mathbf{x}\in X}\left(1-\frac{\bar{\rho}\mathbb{1}[\mathbf{x}\in B_{\mathbb{S}^2}(\mathbf{p},r)]}{\rho(\mathbf{x})}\right)\\ &= \frac{1}{|P|^2}\mathbb{E}\left(\sum_{\mathbf{p}\in P}\prod_{\mathbf{x}\in X}\left(1-\frac{\bar{\rho}\mathbb{1}[\mathbf{x}\in B_{\mathbb{S}^2}(\mathbf{p},r)]}{\rho(\mathbf{x})}\right)\right)^2 - \frac{1}{|P|^2}\mathbb{E}^2\sum_{\mathbf{p}\in P}\prod_{\mathbf{x}\in X}\left(1-\frac{\bar{\rho}\mathbb{1}[\mathbf{x}\in B_{\mathbb{S}^2}(\mathbf{p},r)]}{\rho(\mathbf{x})}\right)\end{aligned} \tag{13}$$

Dealing with each term independently, we have

$$\begin{aligned}&\mathbb{E}\left(\sum_{\mathbf{p}\in P}\prod_{\mathbf{x}\in X}\left(1-\frac{\bar{\rho}\mathbb{1}[\mathbf{x}\in B_{\mathbb{S}^2}(\mathbf{p},r)]}{\rho(\mathbf{x})}\right)\right)^2\\ &\quad= \mathbb{E}\sum_{\mathbf{p}\in P}\prod_{\mathbf{x}\in X}\left(1-\frac{\bar{\rho}\mathbb{1}[\mathbf{x}\in B_{\mathbb{S}^2}(\mathbf{p},r)]}{\rho(\mathbf{x})}\right)\sum_{\mathbf{p}'\in P}\prod_{\mathbf{y}\in X}\left(1-\frac{\bar{\rho}\mathbb{1}[\mathbf{y}\in B_{\mathbb{S}^2}(\mathbf{p}',r)]}{\rho(\mathbf{y})}\right)\\ &\quad= \sum_{\mathbf{p}\in P}\sum_{\mathbf{p}'\in P}\mathbb{E}\prod_{\mathbf{x}\in X}\left(1-\frac{\bar{\rho}\mathbb{1}[\mathbf{x}\in B_{\mathbb{S}^2}(\mathbf{p},r)]}{\rho(\mathbf{x})}\right)\prod_{\mathbf{y}\in X}\left(1-\frac{\bar{\rho}\mathbb{1}[\mathbf{y}\in B_{\mathbb{S}^2}(\mathbf{p}',r)]}{\rho(\mathbf{y})}\right)\end{aligned} \tag{14}$$

From the proof of Theorem 1 given by van Lieshout [3], we have the following identity,

$$\prod_{\mathbf{y}\in X}\left(1-\frac{\bar{\rho}\mathbb{1}[\mathbf{y}\in B_{\mathbb{S}^2}(\mathbf{x},r)]}{\rho(\mathbf{y})}\right) = 1 + \sum_{n=1}^{\infty}\frac{(-\bar{\rho})^n}{n!}\sum_{\mathbf{x}_1,\ldots,\mathbf{x}_n\in X}^{\neq}\prod_{i=1}^{n}\frac{\mathbb{1}[\mathbf{x}_i\in B_{\mathbb{S}^2}(\mathbf{x},r)]}{\rho(\mathbf{x}_i)},$$

and using the convention that a sum over an emptyset is 0, i.e. $\sum_{k=1}^{0} = \sum_{x\subseteq\emptyset} = \sum_{\emptyset\in x} = 1$,

$$\prod_{\mathbf{y}\in X}\left(1-\frac{\bar{\rho}\mathbb{1}[\mathbf{y}\in B_{\mathbb{S}^2}(\mathbf{x},r)]}{\rho(\mathbf{y})}\right)=\sum_{n=0}^{\infty}\frac{(-\bar{\rho})^n}{n!}\sum_{\mathbf{x}_1,\ldots,\mathbf{x}_n\in X}^{\neq}\prod_{i=1}^{n}\frac{\mathbb{1}[\mathbf{x}_i\in B_{\mathbb{S}^2}(\mathbf{x},r)]}{\rho(\mathbf{x}_i)}. \tag{15}$$

Taking just the expectation from Equation 14 we expand the first product over $\mathbf{x}$ using the previous identity to give,

$$\mathbb{E}\prod_{\mathbf{x}\in X}\left(1-\frac{\bar{\rho}\mathbb{1}[\mathbf{x}\in B_{\mathbb{S}^2}(\mathbf{p},r)]}{\rho(\mathbf{x})}\right)\prod_{\mathbf{y}\in X}\left(1-\frac{\bar{\rho}\mathbb{1}[\mathbf{y}\in B_{\mathbb{S}^2}(\mathbf{p}',r)]}{\rho(\mathbf{y})}\right)$$

$$=\mathbb{E}\left(\sum_{n=0}^{\infty}\frac{(-\bar{\rho})^n}{n!}\sum_{\mathbf{x}_1,\ldots,\mathbf{x}_n\in X}^{\neq}\prod_{i=1}^{n}\frac{\mathbb{1}[\mathbf{x}_i\in B_{\mathbb{S}^2}(\mathbf{p},r)]}{\rho(\mathbf{x}_i)}\right)\prod_{\mathbf{y}\in X}\left(1-\frac{\bar{\rho}\mathbb{1}[\mathbf{y}\in B_{\mathbb{S}^2}(\mathbf{p}',r)]}{\rho(\mathbf{y})}\right)$$

$$=\mathbb{E}\sum_{n=0}^{\infty}\frac{(-\bar{\rho})^n}{n!}\sum_{\mathbf{x}_1,\ldots,\mathbf{x}_n\in X}^{\neq}\left(\prod_{i=1}^{n}\frac{\mathbb{1}[\mathbf{x}_i\in B_{\mathbb{S}^2}(\mathbf{p},r)]}{\rho(\mathbf{x}_i)}\right.$$
$$\left.\prod_{\mathbf{y}\in\{X\setminus\{\mathbf{x}_1,\ldots,\mathbf{x}_n\},\{\mathbf{x}_1,\ldots,\mathbf{x}_n\}\}}\left(1-\frac{\bar{\rho}\mathbb{1}[\mathbf{y}\in B_{\mathbb{S}^2}(\mathbf{p}',r)]}{\rho(\mathbf{y})}\right)\right)$$

$$=\mathbb{E}\sum_{n=0}^{\infty}\frac{(-\bar{\rho})^n}{n!}\sum_{\mathbf{x}_1,\ldots,\mathbf{x}_n\in X}^{\neq}\left(\prod_{i=1}^{n}\frac{\mathbb{1}[\mathbf{x}_i\in B_{\mathbb{S}^2}(\mathbf{p},r)]}{\rho(\mathbf{x}_i)}\prod_{\mathbf{y}\in\{\mathbf{x}_1,\ldots,\mathbf{x}_n\}}\left(1-\frac{\bar{\rho}\mathbb{1}[\mathbf{y}\in B_{\mathbb{S}^2}(\mathbf{p}',r)]}{\rho(\mathbf{y})}\right)\right.$$
$$\left.\prod_{\mathbf{y}\in X\setminus\{\mathbf{x}_1,\ldots,\mathbf{x}_n\}}\left(1-\frac{\bar{\rho}\mathbb{1}[\mathbf{y}\in B_{\mathbb{S}^2}(\mathbf{p}',r)]}{\rho(\mathbf{y})}\right)\right)$$

$$=\mathbb{E}\sum_{n=0}^{\infty}\frac{(-\bar{\rho})^n}{n!}\sum_{\mathbf{x}_1,\ldots,\mathbf{x}_n\in X}^{\neq}\left(\prod_{i=1}^{n}\frac{\mathbb{1}[\mathbf{x}_i\in B_{\mathbb{S}^2}(\mathbf{p},r)]}{\rho(\mathbf{x}_i)}\left(1-\frac{\bar{\rho}\mathbb{1}[\mathbf{x}_i\in B_{\mathbb{S}^2}(\mathbf{p}',r)]}{\rho(\mathbf{x}_i)}\right)\right.$$
$$\left.\prod_{\mathbf{y}\in X\setminus\{\mathbf{x}_1,\ldots,\mathbf{x}_n\}}\left(1-\frac{\bar{\rho}\mathbb{1}[\mathbf{y}\in B_{\mathbb{S}^2}(\mathbf{p}',r)]}{\rho(\mathbf{y})}\right)\right)$$

$$=\sum_{n=0}^{\infty}\frac{(-\bar{\rho})^n}{n!}\mathbb{E}\sum_{\mathbf{x}_1,\ldots,\mathbf{x}_n\in X}^{\neq}\left(\prod_{i=1}^{n}\frac{\mathbb{1}[\mathbf{x}_i\in B_{\mathbb{S}^2}(\mathbf{p},r)]}{\rho(\mathbf{x}_i)}\left(1-\frac{\bar{\rho}\mathbb{1}[\mathbf{x}_i\in B_{\mathbb{S}^2}(\mathbf{p}',r)]}{\rho(\mathbf{x}_i)}\right)\right.$$
$$\left.\prod_{\mathbf{y}\in X\setminus\{\mathbf{x}_1,\ldots,\mathbf{x}_n\}}\left(1-\frac{\bar{\rho}\mathbb{1}[\mathbf{y}\in B_{\mathbb{S}^2}(\mathbf{p}',r)]}{\rho(\mathbf{y})}\right)\right)$$

Using the extended Slivnyak-Mecke Theorem,

$$=\sum_{n=0}^{\infty}\frac{(-\bar{\rho})^n}{n!}\overbrace{\int_{\mathbb{S}^2}\cdots\int_{\mathbb{S}^2}}^{n}$$

$$\mathbb{E}\left(\prod_{i=1}^{n}\frac{\mathbb{1}[\mathbf{x}_i\in B_{\mathbb{S}^2}(\mathbf{p},r)]}{\rho(\mathbf{x}_i)}\left(1-\frac{\bar{\rho}\mathbb{1}[\mathbf{x}_i\in B_{\mathbb{S}^2}(\mathbf{p}',r)]}{\rho(\mathbf{x}_i)}\right)\right.$$
$$\left.\prod_{\mathbf{y}\in X}\left(1-\frac{\bar{\rho}\mathbb{1}[\mathbf{y}\in B_{\mathbb{S}^2}(\mathbf{p}',r)]}{\rho(\mathbf{y})}\right)\right)\prod_{i=1}^{n}\rho(\mathbf{x}_i)\lambda_{\mathbb{S}^2}(d\mathbf{x}_i)$$
$$=\sum_{n=0}^{\infty}\frac{(-\bar{\rho})^n}{n!}\overbrace{\int_{\mathbb{S}^2}\cdots\int_{\mathbb{S}^2}}^{n}$$
$$\prod_{i=1}^{n}\frac{\mathbb{1}[\mathbf{x}_i\in B_{\mathbb{S}^2}(\mathbf{p},r)]}{\rho(\mathbf{x}_i)}\left(1-\frac{\bar{\rho}\mathbb{1}[\mathbf{x}_i\in B_{\mathbb{S}^2}(\mathbf{p}',r)]}{\rho(\mathbf{x}_i)}\right)$$
$$\mathbb{E}\prod_{\mathbf{y}\in X}\left(1-\frac{\bar{\rho}\mathbb{1}[\mathbf{y}\in B_{\mathbb{S}^2}(\mathbf{p}',r)]}{\rho(\mathbf{y})}\right)\prod_{i=1}^{n}\rho(\mathbf{x}_i)\lambda_{\mathbb{S}^2}(d\mathbf{x}_i)$$
$$=\sum_{n=0}^{\infty}\frac{(-\bar{\rho})^n}{n!}\mathbb{E}\prod_{\mathbf{y}\in X}\left(1-\frac{\bar{\rho}\mathbb{1}[\mathbf{y}\in B_{\mathbb{S}^2}(\mathbf{p}',r)]}{\rho(\mathbf{y})}\right)$$
$$\overbrace{\int_{\mathbb{S}^2}\cdots\int_{\mathbb{S}^2}}^{n}\prod_{i=1}^{n}\frac{\mathbb{1}[\mathbf{x}_i\in B_{\mathbb{S}^2}(\mathbf{p},r)]}{\rho(\mathbf{x}_i)}\left(1-\frac{\bar{\rho}\mathbb{1}[\mathbf{x}_i\in B_{\mathbb{S}^2}(\mathbf{p}',r)]}{\rho(\mathbf{x}_i)}\right)\prod_{i=1}^{n}\rho(\mathbf{x}_i)\lambda_{\mathbb{S}^2}(d\mathbf{x}_i)$$

Note the expectation is just the definition of the generating functional of X which does not depend on the point $\mathbf{p}'$ (see proof of Theorem 1 by van Lieshout [3]),

$$=\sum_{n=0}^{\infty}\frac{(-\bar{\rho})^n}{n!}G(1-u_r^{\mathbf{y}})\overbrace{\int_{\mathbb{S}^2}\cdots\int_{\mathbb{S}^2}}^{n}\prod_{i=1}^{n}\frac{\mathbb{1}[\mathbf{x}_i\in B_{\mathbb{S}^2}(\mathbf{p},r)]}{\rho(\mathbf{x}_i)}$$
$$\left(1-\frac{\bar{\rho}\mathbb{1}[\mathbf{x}_i\in B_{\mathbb{S}^2}(\mathbf{p}',r)]}{\rho(\mathbf{x}_i)}\right)\prod_{i=1}^{n}\rho(\mathbf{x}_i)\lambda_{\mathbb{S}^2}(d\mathbf{x}_i)$$
$$=\sum_{n=0}^{\infty}\frac{(-\bar{\rho})^n}{n!}G(1-u_r^{\mathbf{y}})$$
$$\overbrace{\int_{\mathbb{S}^2\cap B_{\mathbb{S}^2}(\mathbf{p},r)}\cdots\int_{\mathbb{S}^2\cap B_{\mathbb{S}^2}(\mathbf{p},r)}}^{n}\prod_{i=1}^{n}\left(1-\frac{\bar{\rho}\mathbb{1}[\mathbf{x}_i\in B_{\mathbb{S}^2}(\mathbf{p}',r)]}{\rho(\mathbf{x}_i)}\right)\lambda_{\mathbb{S}^2}(d\mathbf{x})_1\cdots\lambda_{\mathbb{S}^2}(d\mathbf{x})_n$$
$$=\sum_{n=0}^{\infty}\frac{(-\bar{\rho})^n}{n!}G(1-u_r^{\mathbf{y}})\left(\int_{\mathbb{S}^2\cap B_{\mathbb{S}^2}(\mathbf{p},r)}\left(1-\frac{\bar{\rho}\mathbb{1}[\mathbf{x}\in B_{\mathbb{S}^2}(\mathbf{p}',r)]}{\rho(\mathbf{x})}\right)\lambda_{\mathbb{S}^2}(d\mathbf{x})\right)^n$$
$$=G(1-u_r^{\mathbf{y}})\sum_{n=0}^{\infty}\frac{(-\bar{\rho})^n}{n!}\left(\lambda_{\mathbb{S}^2}(\mathbb{S}^2_{B_{\mathbb{S}^2}(\mathbf{p},r)})-\int_{\mathbb{S}^2_{B_{\mathbb{S}^2}(\mathbf{p},r)\cap B_{\mathbb{S}^2}(\mathbf{p}',r)}}\frac{\bar{\rho}}{\rho(\mathbf{x})}\lambda_{\mathbb{S}^2}(d\mathbf{x})\right)^n$$

Using the series definition for the exponential function,

$$= G(1-u_r^{\mathbf{y}}) \exp\left(-\bar{\rho}\left(\lambda_{\mathbb{S}^2}(\mathbb{S}^2_{B_{\mathbb{S}^2}(\mathbf{p},r)}) - \int_{\mathbb{S}^2_{B_{\mathbb{S}^2}(\mathbf{o},r)\cap B_{\mathbb{S}^2}(\mathbf{p}',r)}} \frac{\bar{\rho}}{\rho(\mathbf{x})}\lambda_{\mathbb{S}^2}(d\mathbf{x})\right)\right)$$

$$= G(1-u_r^{\mathbf{y}}) \exp\left(\bar{\rho}\int_{\mathbb{S}^2_{B_{\mathbb{S}^2}(\mathbf{p},r)\cap B_{\mathbb{S}^2}(\mathbf{p}',r)}} \frac{\bar{\rho}}{\rho(\mathbf{x})}\lambda_{\mathbb{S}^2}(d\mathbf{x}) - \bar{\rho}\lambda_{\mathbb{S}^2}(B_{\mathbb{S}^2}(\mathbf{o},r))\right)$$

$$= G(1-u_r^{\mathbf{y}}) \exp\left(-\bar{\rho}\lambda_{\mathbb{S}^2}(B_{\mathbb{S}^2}(\mathbf{o},r))\right) \exp\left(\int_{\mathbb{S}^2_{B_{\mathbb{S}^2}(\mathbf{p},r)\cap B_{\mathbb{S}^2}(\mathbf{p}',r)}} \frac{\bar{\rho}^2}{\rho(\mathbf{x})}\lambda_{\mathbb{S}^2}(d\mathbf{x})\right)$$

Substituting this into the first term of Equation 13 gives,

$$\frac{1}{|P|^2}\mathbb{E}\left(\sum_{\mathbf{p}\in P}\prod_{\mathbf{x}\in X}\left(1-\frac{\bar{\rho}\mathbb{1}[\mathbf{x}\in B_{\mathbb{S}^2}(\mathbf{p},r)]}{\rho(\mathbf{x})}\right)\right)^2$$

$$= G(1-u_r^{\mathbf{y}}) \exp\left(-\bar{\rho}\lambda_{\mathbb{S}^2}(B_{\mathbb{S}^2}(\mathbf{o},r))\right) \frac{1}{|P|^2}\sum_{\mathbf{p}\in P}\sum_{\mathbf{p}'\in P}\exp\left(\int_{\mathbb{S}^2_{B_{\mathbb{S}^2}(\mathbf{p},r)\cap B_{\mathbb{S}^2}(\mathbf{p}',r)}} \frac{\bar{\rho}^2}{\rho(\mathbf{x})}\lambda_{\mathbb{S}^2}(d\mathbf{x})\right)$$

The second term of Equation 13, by unbiasedness of $\hat{F}_{\text{inhom}}$ shown in Theorem 3, gives,

$$\begin{aligned}\mathbb{E}^2\frac{1}{|P|}\sum_{\mathbf{p}\in P}\prod_{\mathbf{x}\in X}\left(1-\frac{\bar{\rho}\mathbb{1}[\mathbf{x}\in B_{\mathbb{S}^2}(\mathbf{p},r)]}{\rho(\mathbf{x})}\right) &= \mathbb{E}^2\left[1-\hat{F}_{\text{inhom}}(r)\right] \\ &= \left(1-\mathbb{E}[\hat{F}_{\text{inhom}}(r)]\right)^2 \\ &= G^2(1-u_r^{\mathbf{y}}),\end{aligned}$$

where the generating functional does not depend on $\mathbf{y}$. Thus the variance is,

$$\begin{aligned}&G(1-u_r^{\mathbf{y}}) \exp\left(-\bar{\rho}\lambda_{\mathbb{S}^2}(B_{\mathbb{S}^2}(\mathbf{o},r))\right) \\ &\quad\times\frac{1}{|P|^2}\sum_{\mathbf{p}\in P}\sum_{\mathbf{p}'\in P}\exp\left(\int_{\mathbb{S}^2_{B_{\mathbb{S}^2}(\mathbf{p},r)\cap B_{\mathbb{S}^2}(\mathbf{p}',r)}} \frac{\bar{\rho}^2}{\rho(\mathbf{x})}\lambda_{\mathbb{S}^2}(d\mathbf{x})\right) - G^2(1-u_r^{\mathbf{y}}),\end{aligned}$$

For a Poisson process, $G(1-u_r^{\mathbf{y}}) = \exp\left(-\bar{\rho}\lambda_{\mathbb{S}^2}(\mathcal{S}^2_{B_{\mathbb{S}^2}(\mathbf{o},r)})\right)$, thus the variance is,

$$\begin{aligned}&\exp\left(-2\bar{\rho}\lambda_{\mathbb{S}^2}(B_{\mathbb{S}^2}(\mathbf{o},r))\right)\frac{1}{|P|^2}\sum_{\mathbf{p}\in P}\sum_{\mathbf{p}'\in P}\exp\left(\int_{\mathbb{S}^2_{B_{\mathbb{S}^2}(\mathbf{p},r)\cap B_{\mathbb{S}^2}(\mathbf{p}',r)}} \frac{\bar{\rho}^2}{\rho(\mathbf{x})}\lambda_{\mathbb{S}^2}(d\mathbf{x})\right) \\ &\quad - \exp\left(-2\bar{\rho}\lambda_{\mathbb{S}^2}(\mathcal{S}^2_{B_{\mathbb{S}^2}(\mathbf{o},r)})\right).\end{aligned}$$

□

Before the proof for the variance of $\hat{H}_{\text{inhom}}(r)$ we introduce the exponential integral. The exponential integral, denoted $\text{Ei}(x)$, is defined as the following integral,

$$\text{Ei}(x) = -\int_{-x}^{\infty} \frac{\text{e}^{-t}}{t} dt.$$

It can then be shown that the exponential integral has the following infinite series representation,

$$\text{Ei}(x) = \gamma + \log(x) + \sum_{k=1}^{\infty} \frac{x^k}{k \cdot k!}, \tag{16}$$

where γ is known as the Euler-Mascheroni constant and defined as,

$$\gamma = \lim_{n \to \infty} \left(-\log(n) + \sum_{k=1}^{n} \frac{1}{k} \right).$$

The variance for $\hat{H}_{\text{inhom}}(r)$ will be given in terms of $\text{Ei}(x)$.

Proof. Variance of $\hat{H}_{inhom}(r)$

Restating the estimator for $\hat{H}_{\text{inhom}}(r)$,

$$\hat{H}_{\text{inhom}}(r) = 1 - \frac{\sum_{\mathbf{x} \in X} \prod_{\mathbf{y} \in X \setminus \{\mathbf{x}\}} \left(1 - \frac{\bar{\rho} \mathbb{1}[\mathbf{y} \in B_{\mathbb{S}^2}(\mathbf{x}, r)]}{\rho(\mathbf{y})}\right)}{N_X(\mathbb{S}^2)},$$

we note that this estimator is only well defined when $\mathbb{1}[N_X(\mathbb{S}^2) > 0]$. In the event that $N_X(\mathbb{S}^2) = 0$ we shall define $\hat{H}_{\text{inhom}}(r) = 0$, in which case we can write our estimator as,

$$\hat{H}_{\text{inhom}}(r) = \mathbb{1}[N_X(\mathbb{S}^2) > 0] \left(1 - \frac{\sum_{\mathbf{x} \in X} \prod_{\mathbf{y} \in X \setminus \{\mathbf{x}\}} \left(1 - \frac{\bar{\rho} \mathbb{1}[\mathbf{y} \in B_{\mathbb{S}^2}(\mathbf{x}, r)]}{\rho(\mathbf{y})}\right)}{N_X(\mathbb{S}^2)} \right).$$

By using the law of total variance, conditioning on $N_X(\mathbb{S}^2) = n$,

$$\text{Var}(\hat{H}_{\text{inhom}}(r)) = \underbrace{\mathbb{E}[\text{Var}(\hat{H}_{\text{inhom}}(r) | N_X(\mathbb{S}^2 = n)]}_{(1)} + \underbrace{\text{Var}(\mathbb{E}[\hat{H}_{\text{inhom}}(r) | N_X(\mathbb{S}^2) = n])}_{(2)}. \tag{17}$$

The variance in term (1) is,

$$\text{Var}(\hat{H}_{\text{inhom}}(r) | N_X(\mathbb{S}^2 = n) = \mathbb{1}[n > 0] \text{Var} \left(1 + \frac{1}{n} \sum_{i=1}^{n} \prod_{\substack{j=1 \\ j \neq i}}^{n} \left(1 - \frac{\bar{\rho} \mathbb{1}[\mathbf{X}_j \in B(\mathbf{X}_i, r)]}{\rho(\mathbf{X}_j)} \right) \right),$$

$$= \frac{\mathbb{1}[n>0]}{n^2} \mathrm{Var}\left(\sum_{i=1}^{n} \prod_{\substack{j=1 \\ j\neq i}}^{n} \left(1 - \frac{\bar{\rho}\mathbb{1}[\mathbf{X}_j \in B(\mathbf{X}_i, r)]}{\rho(\mathbf{X}_j)} \right) \right),$$

where $\mathbf{X}_k,\ k = 1, \ldots, n$ are independently distributed points with density proportional to $\rho(\mathbf{x}_k)$, by definition of a Poisson process. We use the identity $\mathrm{Var}(\sum_{i=1}^{n} X_i) = \sum_{i=1}^{n} \sum_{j=1}^{n} \mathrm{Cov}(X_i, X_j)$ and define $L_i = \prod_{\substack{j=1 \\ j\neq i}}^{n} \left(1 - \frac{\bar{\rho}\mathbb{1}[\mathbf{X}_j \in B(\mathbf{X}_i, r)]}{\rho(\mathbf{X}_j)}\right)$,

$$\begin{aligned}
&= \frac{\mathbb{1}[n>0]}{n^2} \sum_{p=1}^{n} \sum_{q=1}^{n} \mathrm{Cov}\left(L_p, L_q\right) \\
&= \frac{\mathbb{1}[n>0]}{n^2} \sum_{p=1}^{n} \sum_{q=1}^{n} \Big(\mathbb{E}[L_p L_q] - \mathbb{E}[L_p]\mathbb{E}[L_q] \Big) \\
&= \frac{\mathbb{1}[n>0]}{n^2} \sum_{p,q\in\{1,\ldots,n\}}^{\neq} \left(\underbrace{\mathbb{E}[L_p L_q]}_{(a)} - \underbrace{\mathbb{E}[L_p]\mathbb{E}[L_q]}_{(b)} \right) \\
&\quad + \frac{\mathbb{1}[n>0]}{n^2} \sum_{p=1}^{n} \left(\underbrace{\mathbb{E}[L_p^2]}_{(c)} - \underbrace{\mathbb{E}^2[L_p]}_{(d)} \right)
\end{aligned} \tag{18}$$

Taking term (a),

$$\mathbb{E}[L_p L_q] = \mathbb{E}\left[\prod_{\substack{j=1 \\ j\neq p}}^{n} \left(1 - \frac{\bar{\rho}\mathbb{1}[\mathbf{X}_j \in B_{\mathbb{S}^2}(\mathbf{X}_p, r)]}{\rho(\mathbf{X}_j)} \right) \prod_{\substack{j=1 \\ j\neq q}}^{n} \left(1 - \frac{\bar{\rho}\mathbb{1}[\mathbf{X}_j \in B_{\mathbb{S}^2}(\mathbf{X}_q, r)]}{\rho(\mathbf{X}_j)} \right) \right]$$

using iterated expectation, conditioning on $\mathbf{X_p} = \mathbf{x}_p, \mathbf{X_q} = \mathbf{x}_q$,

$$\begin{aligned}
&= \mathbb{E}\Bigg[\mathbb{E}\Bigg[\prod_{\substack{j=1 \\ j\neq p}}^{n} \left(1 - \frac{\bar{\rho}\mathbb{1}[\mathbf{X}_j \in B_{\mathbb{S}^2}(\mathbf{X}_p, r)]}{\rho(\mathbf{X}_j)} \right) \\
&\qquad \prod_{\substack{j=1 \\ j\neq q}}^{n} \left(1 - \frac{\bar{\rho}\mathbb{1}[\mathbf{X}_j \in B_{\mathbb{S}^2}(\mathbf{X}_q, r)]}{\rho(\mathbf{X}_j)} \right) \Bigg| \mathbf{X_p} = \mathbf{x}_p, \mathbf{X_q} = \mathbf{x}_q \Bigg]\Bigg] \\
&= \mathbb{E}\Bigg[\left(1 - \frac{\bar{\rho}\mathbb{1}[\mathbf{X}_p \in B_{\mathbb{S}^2}(\mathbf{X}_q, r)]}{\rho(\mathbf{X}_p)} \right) \left(1 - \frac{\bar{\rho}\mathbb{1}[\mathbf{X}_q \in B_{\mathbb{S}^2}(\mathbf{X}_p, r)]}{\rho(\mathbf{X}_q)} \right)
\end{aligned}$$

$$\times \mathbb{E}\left[\prod_{\substack{j=1\\ j\neq p,q}}^{n}\left(1-\frac{\bar{\rho}\mathbb{1}[\mathbf{X}_j \in B_{\mathbb{S}^2}(\mathbf{X}_p,r)]}{\rho(\mathbf{X}_j)}\right)\left(1-\frac{\bar{\rho}\mathbb{1}[\mathbf{X}_j \in B_{\mathbb{S}^2}(\mathbf{X}_q,r)]}{\rho(\mathbf{X}_j)}\right)\middle|\mathbf{X_p}=\mathbf{x}_p,\mathbf{X_q}=\mathbf{x}_q\right]\Bigg]$$

The expectation conditioned on $(\mathbf{X}_q, \mathbf{X}_p)$ is,

$$\begin{aligned}
&\mathbb{E}\left[\prod_{\substack{j=1\\ j\neq p,q}}^{n}\left(1-\frac{\bar{\rho}\mathbb{1}[\mathbf{X}_j \in B_{\mathbb{S}^2}(\mathbf{X}_p,r)]}{\rho(\mathbf{X}_j)}\right)\left(1-\frac{\bar{\rho}\mathbb{1}[\mathbf{X}_j \in B_{\mathbb{S}^2}(\mathbf{X}_q,r)]}{\rho(\mathbf{X}_j)}\right)\middle|\mathbf{X_p}=\mathbf{x}_p,\mathbf{X_q}=\mathbf{x}_q\right]\\
&\quad=\overbrace{\int_{\mathbb{S}^2}\cdots\int_{\mathbb{S}^2}}^{n-2}\prod_{\substack{j=1\\ j\neq p,q}}^{n}\left(1-\frac{\bar{\rho}\mathbb{1}[\mathbf{x}_j \in B_{\mathbb{S}^2}(\mathbf{x}_p,r)]}{\rho(\mathbf{x}_j)}\right)\left(1-\frac{\bar{\rho}\mathbb{1}[\mathbf{x}_j \in B_{\mathbb{S}^2}(\mathbf{x}_q,r)]}{\rho(\mathbf{x}_j)}\right)\frac{\rho(\mathbf{x}_j)}{\mu(\mathbb{S}^2)}\lambda_{\mathbb{S}^2}(d\mathbf{x}_j)\\
&\quad=\left(\int_{\mathbb{S}^2}\left(1-\frac{\bar{\rho}\mathbb{1}[\mathbf{x} \in B_{\mathbb{S}^2}(\mathbf{x}_p,r)]}{\rho(\mathbf{x})}\right)\left(1-\frac{\bar{\rho}\mathbb{1}[\mathbf{x} \in B_{\mathbb{S}^2}(\mathbf{x}_q,r)]}{\rho(\mathbf{x})}\right)\frac{\rho(\mathbf{x})}{\mu(\mathbb{S}^2)}\lambda_{\mathbb{S}^2}(d\mathbf{x})\right)^{n-2}\\
&\quad=\Bigg(\int_{\mathbb{S}^2}\frac{\rho(\mathbf{x})}{\mu(\mathbb{S}^2)}-\frac{\bar{\rho}}{\mu(\mathbb{S}^2)}\mathbb{1}[\mathbf{x} \in B_{\mathbb{S}^2}(\mathbf{x}_p,r)]-\frac{\bar{\rho}}{\mu(\mathbb{S}^2)}\mathbb{1}[\mathbf{x} \in B_{\mathbb{S}^2}(\mathbf{x}_q,r)]\\
&\qquad\quad+\frac{\bar{\rho}^2}{\mu(\mathbb{S}^2)}\frac{\mathbb{1}[\mathbf{x} \in B_{\mathbb{S}^2}(\mathbf{x}_p,r),\mathbf{x} \in B_{\mathbb{S}^2}(\mathbf{x}_q,r)]}{\rho(\mathbf{x})}\lambda_{\mathbb{S}^2}(d\mathbf{x})\Bigg)^{n-2}\\
&\quad=\left(1-\frac{2\bar{\rho}}{\mu(\mathbb{S}^2)}2\pi(1-\cos r)+\frac{\bar{\rho}^2}{\mu(\mathbb{S}^2)}\int_{B_{\mathbb{S}^2}(\mathbf{x}_p,r)\cap B_{\mathbb{S}^2}(\mathbf{x}_q,r)}\frac{1}{\rho(\mathbf{x})}\lambda_{\mathbb{S}^2}(d\mathbf{x})\right)^{n-2}
\end{aligned}$$

We then define $A_1(\mathbf{x}_p,\mathbf{x}_q) \equiv 1-\frac{2\bar{\rho}}{\mu(\mathbb{S}^2)}2\pi(1-\cos r)+\frac{\bar{\rho}^2}{\mu(\mathbb{S}^2)}\int_{B_{\mathbb{S}^2}(\mathbf{x}_p,r)\cap B_{\mathbb{S}^2}(\mathbf{x}_q,r)}\frac{1}{\rho(\mathbf{x})}\lambda_{\mathbb{S}^2}(d\mathbf{x})$ and returning to $\mathbb{E}[L_pL_q]$,

$$\begin{aligned}
\mathbb{E}[L_pL_q]&=\mathbb{E}\left[\left(1-\frac{\bar{\rho}\mathbb{1}[\mathbf{X}_p \in B_{\mathbb{S}^2}(\mathbf{X}_q,r)]}{\rho(\mathbf{X}_p)}\right)\left(1-\frac{\bar{\rho}\mathbb{1}[\mathbf{X}_q \in B_{\mathbb{S}^2}(\mathbf{X}_p,r)]}{\rho(\mathbf{X}_q)}\right)A_1^{n-2}(\mathbf{X}_p,\mathbf{X}_q)\right]\\
&=\int_{\mathbb{S}^2}\int_{\mathbb{S}^2}\left(1-\frac{\bar{\rho}\mathbb{1}[\mathbf{x}_p \in B_{\mathbb{S}^2}(\mathbf{x}_q,r)]}{\rho(\mathbf{x}_p)}\right)\left(1-\frac{\bar{\rho}\mathbb{1}[\mathbf{x}_q \in B_{\mathbb{S}^2}(\mathbf{x}_p,r)]}{\rho(\mathbf{x}_q)}\right)\\
&\qquad\qquad A_1^{n-2}(\mathbf{x}_p,\mathbf{x}_q)\frac{\rho(\mathbf{x}_p)\rho(\mathbf{x}_p)}{\mu^2(\mathbb{S}^2)}\lambda_{\mathbb{S}^2}(d\mathbf{x}_p)\lambda_{\mathbb{S}^2}(d\mathbf{x}_q)\\
&=\frac{1}{\mu^2(\mathbb{S}^2)}\int_{\mathbb{S}^2}\int_{\mathbb{S}^2}\left(\rho(\mathbf{x}_p)-\bar{\rho}\mathbb{1}[\mathbf{x}_p \in B_{\mathbb{S}^2}(\mathbf{x}_q,r)]\right)\left(\rho(\mathbf{x}_q)-\bar{\rho}\mathbb{1}[\mathbf{x}_q \in B_{\mathbb{S}^2}(\mathbf{x}_p,r)]\right)\\
&\qquad\qquad A_1^{n-2}(\mathbf{x}_p,\mathbf{x}_q)\lambda_{\mathbb{S}^2}(d\mathbf{x}_p)\lambda_{\mathbb{S}^2}(d\mathbf{x}_q)\\
&=\frac{1}{\mu^2(\mathbb{S}^2)}\int_{\mathbb{S}^2}\int_{\mathbb{S}^2}\left(\rho(\mathbf{x})-\bar{\rho}\mathbb{1}[\mathbf{x} \in B_{\mathbb{S}^2}(\mathbf{y},r)]\right)\left(\rho(\mathbf{y})-\bar{\rho}\mathbb{1}[\mathbf{y} \in B_{\mathbb{S}^2}(\mathbf{x},r)]\right)\\
&\qquad\qquad A_1^{n-2}(\mathbf{x},\mathbf{y})\lambda_{\mathbb{S}^2}(d\mathbf{x})\lambda_{\mathbb{S}^2}(d\mathbf{y})
\end{aligned}$$

Then taking term (b) of Equation 18 and examining one of the expectations,

$$\mathbb{E}[L_p] = \mathbb{E}\left[\prod_{\substack{j=1\\j\neq p}}^{n}\left(1 - \frac{\bar{\rho}\mathbb{1}[\mathbf{X}_j \in B_{\mathbb{S}^2}(\mathbf{X}_p, r)]}{\rho(\mathbf{X}_j)}\right)\right]$$

using iterated expectation conditioning on $\mathbf{X}_p = \mathbf{x}_p$,

$$= \mathbb{E}\left[\mathbb{E}\left[\prod_{\substack{j=1\\j\neq p}}^{n}\left(1 - \frac{\bar{\rho}\mathbb{1}[\mathbf{X}_j \in B_{\mathbb{S}^2}(\mathbf{X}_p, r)]}{\rho(\mathbf{X}_j)}\right)\middle|\mathbf{X}_p = \mathbf{x}_p\right]\right]$$
$$= \mathbb{E}\left[\mathbb{E}\left[\prod_{\substack{j=1\\j\neq p}}^{n}\left(1 - \frac{\bar{\rho}\mathbb{1}[\mathbf{X}_j \in B_{\mathbb{S}^2}(\mathbf{x}_p, r)]}{\rho(\mathbf{X}_j)}\right)\right]\right].$$

Taking the condition expectation,

$$\mathbb{E}\left[\prod_{\substack{j=1\\j\neq p}}^{n}\left(1 - \frac{\bar{\rho}\mathbb{1}[\mathbf{X}_j \in B_{\mathbb{S}^2}(\mathbf{x}_p, r)]}{\rho(\mathbf{X}_j)}\right)\right] = \overbrace{\int_{\mathbb{S}^2}\cdots\int_{\mathbb{S}^2}}^{n-1}\prod_{\substack{j=1\\j\neq p}}^{n}\left(1 - \frac{\bar{\rho}\mathbb{1}[\mathbf{x}_j \in B_{\mathbb{S}^2}(\mathbf{x}_p, r)]}{\rho(\mathbf{x}_j)}\right)\frac{\rho(\mathbf{x}_j)}{\mu(\mathbb{S}^2)}\lambda_{\mathbb{S}^2}(d\mathbf{x}_j)$$
$$= \left(\int_{\mathbb{S}^2}\left(1 - \frac{\bar{\rho}\mathbb{1}[\mathbf{x} \in B_{\mathbb{S}^2}(\mathbf{x}_p, r)]}{\rho(\mathbf{x})}\right)\frac{\rho(\mathbf{x})}{\mu(\mathbb{S}^2)}\lambda_{\mathbb{S}^2}(d\mathbf{x})\right)^{n-1}$$
$$= \left(1 - \frac{\bar{\rho}}{\mu(\mathbb{S}^2)}2\pi(1-\cos r)\right)^{n-1}.$$

Hence,

$$\mathbb{E}[L_p] = \left(1 - \frac{\bar{\rho}}{\mu(\mathbb{S}^2)}2\pi(1-\cos r)\right)^{n-1}$$

Next we will deal with term (c).

$$\mathbb{E}[L_p^2] = \mathbb{E}\left[\left(\prod_{\substack{j=1\\j\neq p}}^{n}\left(1 - \frac{\bar{\rho}\mathbb{1}[\mathbf{X}_j \in B_{\mathbb{S}^2}(\mathbf{X}_p, r)]}{\rho(\mathbf{X}_j)}\right)\right)^2\right]$$
$$= \mathbb{E}\left[\prod_{\substack{j=1\\j\neq p}}^{n}\left(1 - \frac{\bar{\rho}\mathbb{1}[\mathbf{X}_j \in B_{\mathbb{S}^2}(\mathbf{X}_p, r)]}{\rho(\mathbf{X}_j)}\right)^2\right]$$

$$= \mathbb{E}\left[\mathbb{E}\left[\prod_{\substack{j=1\\j\neq p}}^{n}\left(1-\frac{\bar{\rho}\mathbb{1}[\mathbf{X}_j \in B_{\mathbb{S}^2}(\mathbf{x}_p, r)]}{\rho(\mathbf{X}_j)}\right)^2 \middle| \mathbf{X}_p = \mathbf{x}_p\right]\right]$$

$$= \mathbb{E}\left[\prod_{\substack{j=1\\j\neq p}}^{n}\mathbb{E}\left[\left(1-\frac{\bar{\rho}\mathbb{1}[\mathbf{X}_j \in B_{\mathbb{S}^2}(\mathbf{x}_p, r)]}{\rho(\mathbf{X}_j)}\right)^2 \middle| \mathbf{X}_p = \mathbf{x}_p\right]\right]$$

$$= \mathbb{E}\left[\prod_{\substack{j=1\\j\neq p}}^{n}\int_{\mathbb{S}^2}\left(1-\frac{\bar{\rho}\mathbb{1}[\mathbf{x} \in B_{\mathbb{S}^2}(\mathbf{x}_p, r)]}{\rho(\mathbf{x})}\right)^2 \frac{\rho(\mathbf{x})}{\mu(\mathbb{S}^2)}\lambda_{\mathbb{S}^2}(d\mathbf{x})\right]$$

$$= \mathbb{E}\left[\prod_{\substack{j=1\\j\neq p}}^{n}\int_{\mathbb{S}^2}\frac{\rho(\mathbf{x})}{\mu(\mathbb{S}^2)} - \frac{2\bar{\rho}}{\mu(\mathbb{S}^2)}\mathbb{1}[\mathbf{x} \in B_{\mathbb{S}^2}(\mathbf{x}_q, r)] + \frac{\bar{\rho}^2}{\mu(\mathbb{S}^2)}\frac{\mathbb{1}[\mathbf{x} \in B_{\mathbb{S}^2}(\mathbf{x}_p, r)]}{\rho(\mathbf{x})}\lambda_{\mathbb{S}^2}(d\mathbf{x})\right]$$

$$= \mathbb{E}\left[\prod_{\substack{j=1\\j\neq p}}^{n}\left(1-\frac{2\bar{\rho}}{\mu(\mathbb{S}^2)}2\pi(1-\cos r) + \frac{\bar{\rho}^2}{\mu(\mathbb{S}^2)}\int_{\mathbb{S}^2\cap B_{\mathbb{S}^2}(\mathbf{x}_p, r)}\frac{1}{\rho(\mathbf{x})}\lambda_{\mathbb{S}^2}(d\mathbf{x})\right)\right]$$

$$= \mathbb{E}\left[\left(1-\frac{2\bar{\rho}}{\mu(\mathbb{S}^2)}2\pi(1-\cos r) + \frac{\bar{\rho}^2}{\mu(\mathbb{S}^2)}\int_{\mathbb{S}^2\cap B_{\mathbb{S}^2}(\mathbf{x}_p, r)}\frac{1}{\rho(\mathbf{x})}\lambda_{\mathbb{S}^2}(d\mathbf{x})\right)^{n-1}\right]$$

$$= \int_{\mathbb{S}^2}\left(1-\frac{2\bar{\rho}}{\mu(\mathbb{S}^2)}2\pi(1-\cos r) + \frac{\bar{\rho}^2}{\mu(\mathbb{S}^2)}\int_{\mathbb{S}^2\cap B_{\mathbb{S}^2}(\mathbf{y}, r)}\frac{1}{\rho(\mathbf{x})}\lambda_{\mathbb{S}^2}(d\mathbf{x})\right)^{n-1}\frac{\rho(\mathbf{y})}{\mu(\mathbb{S}^2)}\lambda_{\mathbb{S}^2}(d\mathbf{y})$$

Let us define $A_2(\mathbf{y}) = 1 - \frac{2\bar{\rho}}{\mu(\mathbb{S}^2)}2\pi(1-\cos r) + \frac{\bar{\rho}^2}{\mu(\mathbb{S}^2)}\int_{\mathbb{S}^2\cap B_{\mathbb{S}^2}(\mathbf{y},r)}\frac{1}{\rho(\mathbf{x})}\lambda_{\mathbb{S}^2}(d\mathbf{x})$ then,

$$= \frac{1}{\mu(\mathbb{S}^2)}\int_{\mathbb{S}^2}A_2^{n-1}(\mathbf{y})\rho(\mathbf{y})\lambda_{\mathbb{S}^2}(d\mathbf{y})$$

The final term (d) is identical to that of (b). So plugging into Equation 18 gives,

$$\mathrm{Var}(\hat{H}_{\mathrm{inhom}}(r)|N_X(\mathbb{S}^2 = n) = \frac{\mathbb{1}[n>0]}{n^2}\sum_{p,q\in\{1,\dots,n\}}^{\neq}$$

$$\left(\frac{1}{\mu^2(\mathbb{S}^2)}\int_{\mathbb{S}^2}\int_{\mathbb{S}^2}\left(\rho(\mathbf{x}) - \bar{\rho}\mathbb{1}[\mathbf{x}\in B_{\mathbb{S}^2}(\mathbf{y},r)]\right)\left(\rho(\mathbf{y}) - \bar{\rho}\mathbb{1}[\mathbf{y}\in B_{\mathbb{S}^2}(\mathbf{x},r)]\right)A_1^{n-2}(\mathbf{x},\mathbf{y})\lambda_{\mathbb{S}^2}(d\mathbf{x})\lambda_{\mathbb{S}^2}(d\mathbf{y})\right.$$

$$\left. - \left(1-\frac{\bar{\rho}}{\mu(\mathbb{S}^2)}2\pi(1-\cos r)\right)^{n-1}\left(1-\frac{\bar{\rho}}{\mu(\mathbb{S}^2)}2\pi(1-\cos r)\right)^{n-1}\right)$$

$$+\frac{\mathbb{1}[n>0]}{n^2}\sum_{p=1}^{n}\left(\frac{1}{\mu(\mathbb{S}^2)}\int_{\mathbb{S}^2}A_2^{n-1}(\mathbf{y})\rho(\mathbf{y})\lambda_{\mathbb{S}^2}(d\mathbf{y})-\left(1-\frac{\bar{\rho}}{\mu(\mathbb{S}^2)}2\pi(1-\cos r)\right)^{2n-2}\right)$$

$$=\frac{1}{\mu^2(\mathbb{S}^2)}$$

$$\int_{\mathbb{S}^2}\int_{\mathbb{S}^2}\left(\rho(\mathbf{x})-\bar{\rho}\mathbb{1}[\mathbf{x}\in B_{\mathbb{S}^2}(\mathbf{y},r)]\right)\left(\rho(\mathbf{y})-\bar{\rho}\mathbb{1}[\mathbf{y}\in B_{\mathbb{S}^2}(\mathbf{x},r)]\right)$$

$$\left(\frac{\mathbb{1}[n>0](n-1)}{n}A_1^{n-2}(\mathbf{x},\mathbf{y})\right)\lambda_{\mathbb{S}^2}(d\mathbf{x})\lambda_{\mathbb{S}^2}(d\mathbf{y})$$

$$+\frac{1}{\mu(\mathbb{S}^2)}\int_{\mathbb{S}^2}\left(\frac{\mathbb{1}[n>0]}{n}A_2^{n-1}(\mathbf{y})\right)\rho(\mathbf{y})\lambda_{\mathbb{S}^2}(d\mathbf{y})-\mathbb{1}[n>0]\left(1-\frac{\bar{\rho}}{\mu(\mathbb{S}^2)}2\pi(1-\cos r)\right)^{2n-2}$$

We need to then take the expectation of this variance over $N_X(\mathbb{S}^2)$. By application of Tonelli's Theorem we can interchange the expectation over $N_X(\mathbb{S}^2)$ with the integrals over $\mathbf{x}$ and $\mathbf{y}$. This comes by showing that both $A_1(\mathbf{x},\mathbf{y})$ and $A_2(\mathbf{x})$ are non-negative for all $\mathbf{x},\mathbf{y}\in\mathbb{S}^2$. Obviously $\rho(\mathbf{x})-\bar{\rho}\mathbb{1}[\mathbf{x}\in B_{\mathbb{S}^2}(\mathbf{y},r)]$ and $\rho(\mathbf{y})-\bar{\rho}\mathbb{1}[\mathbf{y}\in B_{\mathbb{S}^2}(\mathbf{x},r)]$ are greater than or equal to 0 since $\bar{\rho}=\inf_{\mathbf{x}\in\mathbb{S}^2}\rho(\mathbf{x})$. It then follows since the integrand of $A_1(\mathbf{x}_p,\mathbf{x}_q)$, $\left(1-\bar{\rho}\mathbb{1}[\mathbf{x}\in B_{\mathbb{S}^2}(\mathbf{x}_p,r)]/\rho(\mathbf{x})\right)\left(1-\bar{\rho}\mathbb{1}[\mathbf{x}\in B_{\mathbb{S}^2}(\mathbf{x}_q,r)]/\rho(\mathbf{x})\right)\rho(\mathbf{x})/\mu(\mathbb{S}^2)$, is then non-negative for all $\mathbf{x}$ and so the integral over $\mathbf{f}$ is non-negative and thus Tonelli's Theorem can be applied. A near identitical argument can be applied to $A_2(\mathbf{x})$ to show that it is always non-negative and hence Tonelli's Theorem can then be applied. We then calculate the following expectations,

a) $\mathbb{E}\left[\frac{\mathbb{1}[N_X(\mathbb{S}^2)>0](N_X(\mathbb{S}^2)-1)}{N_X(\mathbb{S}^2)}A_1^{N_X(\mathbb{S}^2)-2}(\mathbf{x},\mathbf{y})\right]$

b) $\mathbb{E}\left[\frac{\mathbb{1}[N_X(\mathbb{S}^2)>0]}{N_X(\mathbb{S}^2)}A_2^{N_X(\mathbb{S}^2)-1}(\mathbf{x})\right]$

c) $\mathbb{E}\left[\mathbb{1}[N_X(\mathbb{S}^2)>0]\left(1-\frac{\bar{\rho}}{\mu(\mathbb{S}^2)}2\pi(1-\cos r)\right)^{2N_X(\mathbb{S}^2)-2}\right]$

Setting $\Lambda=\mu(\mathbb{S}^2)$, expectation (a) is,

$$\mathbb{E}\left[\frac{\mathbb{1}[N_X(\mathbb{S}^2)>0](N_X(\mathbb{S}^2)-1)}{N_X(\mathbb{S}^2)}A_1^{N_X(\mathbb{S}^2)-2}(\mathbf{x},\mathbf{y})\right]$$

$$=\mathbb{E}\left[\mathbb{1}[N_X(\mathbb{S}^2)>0]A_1^{N_X(\mathbb{S}^2)-2}(\mathbf{x},\mathbf{y})\right]-\mathbb{E}\left[\frac{\mathbb{1}[N_X(\mathbb{S}^2)>0]}{N_X(\mathbb{S}^2)}A_1^{N_X(\mathbb{S}^2)-2}(\mathbf{x},\mathbf{y})\right]$$

$$=\sum_{n=0}^{\infty}\mathbb{1}[n>0]A_1^{n-2}(\mathbf{x},\mathbf{y})\frac{\Lambda^n\mathrm{e}^{-\Lambda}}{n!}-\sum_{n=0}^{\infty}\frac{\mathbb{1}[n>0]}{n}A_1^{n-2}(\mathbf{x},\mathbf{y})\frac{\Lambda^n\mathrm{e}^{-\Lambda}}{n!}$$

$$=\frac{\mathrm{e}^{-\Lambda}}{A_1^2(\mathbf{x},\mathbf{y})}\sum_{n=1}^{\infty}\frac{(\Lambda A_1(\mathbf{x},\mathbf{y}))^n}{n!}-\frac{\mathrm{e}^{-\Lambda}}{A_1^2(\mathbf{x},\mathbf{y})}\sum_{n=1}^{\infty}\frac{1}{n}\frac{(\Lambda A_1(\mathbf{x},\mathbf{y}))^n}{n!}$$

$$= \frac{\mathrm{e}^{-\Lambda}}{A_1^2(\mathbf{x},\mathbf{y})}\left(\sum_{n=0}^{\infty}\frac{(\Lambda A_1(\mathbf{x},\mathbf{y}))^n}{n!}-1\right)-\frac{\mathrm{e}^{-\Lambda}}{A_1^2(\mathbf{x},\mathbf{y})}(\mathrm{Ei}(\Lambda A_1(\mathbf{x},\mathbf{y}))-\gamma-\log(\Lambda A_1(\mathbf{x},\mathbf{y})))$$
$$= \frac{\mathrm{e}^{-\Lambda}}{A_1^2(\mathbf{x},\mathbf{y})}\left(\mathrm{e}^{\Lambda A_1(\mathbf{x},\mathbf{y})}-1-\mathrm{Ei}(\Lambda A_1(\mathbf{x},\mathbf{y}))+\gamma+\log(\Lambda A_1(\mathbf{x},\mathbf{y}))\right)$$

where the penultimate line follows from Equation 16. Similarly for (b) and (c),

$$\mathbb{E}\left[\frac{\mathbb{1}[N_X(\mathbb{S}^2)>0]}{N_X(\mathbb{S}^2)}A_2^{N_X(\mathbb{S}^2)-1}(\mathbf{x})\right]=\frac{\mathrm{e}^{-\Lambda}}{A_2(\mathbf{x})}\left(\gamma+\log(\Lambda A_2(\mathbf{x}))-\mathrm{Ei}(\Lambda A_2(\mathbf{x}))\right)$$
$$\mathbb{E}\left[\mathbb{1}[N_X(\mathbb{S}^2)>0]C^{2N_X(\mathbb{S}^2)-2}\right]=\frac{\mathrm{e}^{-\Lambda}}{C^2}\left(\mathrm{e}^{\Lambda C^2}-1\right),$$

where $C=\left(1-\frac{\bar{\rho}}{\mu(\mathbb{S}^2)}2\pi(1-\cos r)\right)$. Then taking expectations of $\mathrm{Var}(\hat{H}_{\mathrm{inhom}}(r)|N_X(\mathbb{S}^2)=n)$ over $N_X(\mathbb{S}^2)$ gives,

$$\begin{aligned}
&\mathbb{E}[\mathrm{Var}(\hat{H}_{\mathrm{inhom}}(r)|N_X(\mathbb{S}^2)=n)]\\
&=\frac{1}{\mu^2(\mathbb{S}^2)}\\
&\quad\int_{\mathbb{S}^2}\int_{\mathbb{S}^2}(\rho(\mathbf{x})-\bar{\rho}\mathbb{1}[\mathbf{x}\in B_{\mathbb{S}^2}(\mathbf{y},r)])(\rho(\mathbf{y})-\bar{\rho}\mathbb{1}[\mathbf{y}\in B_{\mathbb{S}^2}(\mathbf{x},r)])\\
&\qquad\frac{\mathrm{e}^{-\Lambda}}{A_1^2(\mathbf{x},\mathbf{y})}\left(\mathrm{e}^{\Lambda A_1(\mathbf{x},\mathbf{y})}-1-\mathrm{Ei}(\Lambda A_1(\mathbf{x},\mathbf{y}))+\gamma+\log(\Lambda A_1(\mathbf{x},\mathbf{y}))\right)\lambda_{\mathbb{S}^2}(d\mathbf{x})\lambda_{\mathbb{S}^2}(d\mathbf{y})\\
&\quad+\frac{1}{\mu(\mathbb{S}^2)}\int_{\mathbb{S}^2}\frac{\mathrm{e}^{-\Lambda}}{A_2(\mathbf{x})}\left(\gamma+\log(\Lambda A_2(\mathbf{x}))-\mathrm{Ei}(\Lambda A_2(\mathbf{x}))\right)\rho(\mathbf{y})\lambda_{\mathbb{S}^2}(d\mathbf{y})\\
&\quad-\frac{\mathrm{e}^{-\Lambda}}{\left(1-\frac{\bar{\rho}}{\mu(\mathbb{S}^2)}2\pi(1-\cos r)\right)^2}\left(\mathrm{e}^{\Lambda\left(1-\frac{\bar{\rho}}{\mu(\mathbb{S}^2)}2\pi(1-\cos r)\right)^2}-1\right)
\end{aligned}$$

Term (2) of Equation 17, the expectation conditioned on $N_X(\mathbb{S}^2)=n$,

$$\begin{aligned}
&\mathbb{E}[\hat{H}_{\mathrm{inhom}}(r)|N_X(\mathbb{S}^2)=n]=\\
&\qquad\mathbb{E}\left[1-\frac{\mathbb{1}[N_X(\mathbb{S}^2)>0]}{N_X(\mathbb{S}^2)}\sum_{\mathbf{x}\in X}\prod_{\mathbf{y}\in X\setminus\{\mathbf{x}\}}\left(1-\frac{\bar{\rho}\mathbb{1}[\mathbf{y}\in B_{\mathbb{S}^2}(\mathbf{x},r)]}{\rho(\mathbf{y})}\right)\middle|N_X(\mathbb{S}^2)=n\right]\\
&\qquad=1-\frac{\mathbb{1}[n>0]}{n}\sum_{i=1}^{n}\mathbb{E}\left[\prod_{\substack{j=1\\j\neq i}}^{n}\left(1-\frac{\bar{\rho}\mathbb{1}[\mathbf{x}_j\in B(\mathbf{x}_i,r)]}{\rho(\mathbf{x}_j)}\right)\right]\\
&\qquad=1-\frac{\mathbb{1}[n>0]}{n}\sum_{i=1}^{n}\mathbb{E}\left[L_i\right],
\end{aligned}$$

where L_i is as defined in Equation 18,

$$= 1 - \frac{\mathbb{1}[n > 0]}{n} \sum_{i=1}^{n} \left(1 - \frac{\bar{\rho}}{\mu(\mathbb{S}^2)} 2\pi(1 - \cos r)\right)^{n-1}$$
$$= 1 - \mathbb{1}[n > 0] \left(1 - \frac{\bar{\rho}}{\mu(\mathbb{S}^2)} 2\pi(1 - \cos r)\right)^{n-1}$$
$$= 1 - \mathbb{1}[n > 0] C^{n-1},$$

where $C = 1 - \frac{\bar{\rho}}{\mu(\mathbb{S}^2)} 2\pi(1 - \cos r)$. Then taking the variance over $N_X(\mathbb{S}^2)$ gives,

$$\begin{aligned}
&\mathrm{Var}(1 - \mathbb{1}[N_X(\mathbb{S}^2) > 0] C^{N_X(\mathbb{S}^2)-1}) \\
&\quad = \mathrm{Var}(\mathbb{1}[N_X(\mathbb{S}^2) > 0] C^{N_X(\mathbb{S}^2)-1}) \\
&\quad = \mathbb{E}\left[\left(\mathbb{1}[N_X(\mathbb{S}^2) > 0] C^{N_X(\mathbb{S}^2)-1}\right)^2\right] - \mathbb{E}^2\left[\mathbb{1}[N_X(\mathbb{S}^2) > 0] C^{N_X(\mathbb{S}^2)-1}\right] \\
&\quad = \mathbb{E}\left[\mathbb{1}[N_X(\mathbb{S}^2) > 0] C^{2N_X(\mathbb{S}^2)-2}\right] - \mathbb{E}^2\left[\mathbb{1}[N_X(\mathbb{S}^2) > 0] C^{N_X(\mathbb{S}^2)-1}\right] \\
&\quad = \frac{\mathrm{e}^{-\Lambda}}{C^2}\left(\mathrm{e}^{\Lambda C^2} - 1\right) - \frac{\mathrm{e}^{-2\Lambda}}{C^2}\left(\mathrm{e}^{\Lambda C} - 1\right)^2,
\end{aligned}$$

where $\Lambda = \mu(\mathbb{S}^2)$. Therefore the variance of $\hat{H}_{\mathrm{inhom}}(r)$ is,

$$\begin{aligned}
&\mathrm{Var}(\hat{H}_{\mathrm{inhom}}(r)) \\
&= \frac{1}{\mu^2(\mathbb{S}^2)} \int_{\mathbb{S}^2}\int_{\mathbb{S}^2} \left(\rho(\mathbf{x}) - \bar{\rho}\mathbb{1}[\mathbf{x} \in B_{\mathbb{S}^2}(\mathbf{y}, r)]\right)\left(\rho(\mathbf{y}) - \bar{\rho}\mathbb{1}[\mathbf{y} \in B_{\mathbb{S}^2}(\mathbf{x}, r)]\right) \\
&\frac{\mathrm{e}^{-\mu(\mathbb{S}^2)}}{A_1^2(\mathbf{x}, \mathbf{y})}\left(\mathrm{e}^{\mu(\mathbb{S}^2) A_1(\mathbf{x},\mathbf{y})} - 1 - \mathrm{Ei}(\mu(\mathbb{S}^2) A_1(\mathbf{x}, \mathbf{y})) + \gamma + \log(\mu(\mathbb{S}^2) A_1(\mathbf{x}, \mathbf{y}))\right) \lambda_{\mathbb{S}^2}(d\mathbf{x}) \lambda_{\mathbb{S}^2}(d\mathbf{y}) \\
&\quad + \frac{1}{\mu(\mathbb{S}^2)} \int_{\mathbb{S}^2} \frac{\mathrm{e}^{-\mu(\mathbb{S}^2)}}{A_2(\mathbf{x})}\left(\gamma + \log(\mu(\mathbb{S}^2) A_2(\mathbf{x})) - \mathrm{Ei}(\mu(\mathbb{S}^2) A_2(\mathbf{x}))\right) \rho(\mathbf{y}) \lambda_{\mathbb{S}^2}(d\mathbf{y}) \\
&\quad - \frac{\mathrm{e}^{-2\mu(\mathbb{S}^2)}}{\left(1 - \frac{\bar{\rho}}{\mu(\mathbb{S}^2)} 2\pi(1 - \cos r)\right)^2}\left(\mathrm{e}^{\mu(\mathbb{S}^2)\left(1 - \frac{\bar{\rho}}{\mu(\mathbb{S}^2)} 2\pi(1 - \cos r)\right)} - 1\right)^2
\end{aligned} \tag{19}$$

The final part of the proof is to ensure that the integrands are truly Lebesgue integrable, that is the integrals given in the previous equation are finite. We shall work with the second, $\int_{\mathbb{S}^2} (\mathrm{e}^{-\mu(\mathbb{S}^2)}/A_2(\mathbf{x}))\left(\gamma + \log(\mu(\mathbb{S}^2) A_2(\mathbf{x})) - \mathrm{Ei}(\mu(\mathbb{S}^2) A_2(\mathbf{x}))\right) \rho(\mathbf{y}) \lambda_{\mathbb{S}^2}(d\mathbf{y})$, the first then follows by a similar argument. By using the series expansion we know that $\mathrm{Ei}(\mu(\mathbb{S}^2) A_2(\mathbf{x})) -$

$\gamma - \log(\mu(\mathbb{S}^2)A_2(\mathbf{x})) = \sum_{n=1}^{\infty}((\mu(\mathbb{S}^2)A_2(\mathbf{x}))^n)/(n \cdot n!)$ then it can be bound from above as,

$$\begin{aligned}
\mathrm{Ei}(\mu(\mathbb{S}^2)A_2(\mathbf{x})) - \gamma - \log(\mu(\mathbb{S}^2)A_2(\mathbf{x})) \leq \sum_{n=0}^{\infty} \frac{(\mu(\mathbb{S}^2)A_2(\mathbf{x}))^n}{n \cdot n!} \\
\sum_{n=0}^{\infty} \frac{(\mu(\mathbb{S}^2)A_2(\mathbf{x}))^n}{n!} \\
= \mathrm{e}^{\mu(\mathbb{S}^2)A_2(\mathbf{x})},
\end{aligned}$$

and bound from below as,

$$\begin{aligned}
\mathrm{Ei}(\mu(\mathbb{S}^2)A_2(\mathbf{x})) - \gamma - \log(\mu(\mathbb{S}^2)A_2(\mathbf{x})) &\geq \sum_{n=1}^{\infty} \frac{(\mu(\mathbb{S}^2)A_2(\mathbf{x}))^n}{(n+1)!} \\
&= \frac{1}{\mu(\mathbb{S}^2)A_2(\mathbf{x})} \left(\sum_{n=0}^{\infty} \frac{(\mu(\mathbb{S}^2)A_2(\mathbf{x}))^{n+1}}{(n+1)!} - 1 \right) \\
&= \frac{1}{\mu(\mathbb{S}^2)A_2(\mathbf{x})} \left(\mathrm{e}^{\mu(\mathbb{S}^2)A_2(\mathbf{x})} - 1 \right)
\end{aligned}$$

Hence the integrand is bounded,

$$\begin{aligned}
&\frac{\mathrm{e}^{-\mu(\mathbb{S}^2)}}{\mu(\mathbb{S}^2)A_2^2(\mathbf{x})} \left(\mathrm{e}^{\mu(\mathbb{S}^2)A_2(\mathbf{x})} - 1 \right) \leq \\
&\qquad \frac{\mathrm{e}^{-\mu(\mathbb{S}^2)}}{A_2(\mathbf{x})} \left(\gamma + \log(\mu(\mathbb{S}^2)A_2(\mathbf{x})) - \mathrm{Ei}(\mu(\mathbb{S}^2)A_2(\mathbf{x})) \right) \leq \frac{\mathrm{e}^{-\mu(\mathbb{S}^2)}}{A_2(\mathbf{x})} \mathrm{e}^{\mu(\mathbb{S}^2)A_2(\mathbf{x})}.
\end{aligned} \tag{20}$$

We show that the lower and upper bounds can be bounded further such that they do not depend on r or $\mathbf{x}$. First we bound $\mathrm{e}^{-\mu(\mathbb{S}^2)}\mathrm{e}^{\mu(\mathbb{S}^2)A_2(\mathbf{x})}$ below and above,

$$\begin{aligned}
\mathrm{e}^{-\mu(\mathbb{S}^2)}\mathrm{e}^{\mu(\mathbb{S}^2)A_2(\mathbf{x})} &= \exp\left(-4\pi\bar{\rho}(1-\cos r) + \frac{\bar{\rho}^2}{\mu(\mathbb{S}^2)} \int_{\mathbb{S}^2 \cap B_{\mathbb{S}^2}(\mathbf{x},r)} \frac{1}{\rho(\mathbf{y})} \lambda_{\mathbb{S}^2}(d\mathbf{y}) \right) \\
&\leq \exp\left(\frac{\bar{\rho}^2}{\mu(\mathbb{S}^2)} \int_{\mathbb{S}^2 \cap B_{\mathbb{S}^2}(\mathbf{x},r)} \frac{1}{\rho(\mathbf{y})} \lambda_{\mathbb{S}^2}(d\mathbf{y}) \right) \\
&\leq \exp\left(\frac{\bar{\rho}^2}{\mu(\mathbb{S}^2)} \int_{\mathbb{S}^2 \cap B_{\mathbb{S}^2}(\mathbf{x},r)} \frac{1}{\bar{\rho}} \lambda_{\mathbb{S}^2}(d\mathbf{y}) \right) \\
&= \exp\left(\frac{4\pi\bar{\rho}}{\mu(\mathbb{S}^2)} \right) \\
&\leq \mathrm{e},
\end{aligned}$$

where the final inequality follows from $\mu(\mathbb{S}^2) \geq 4\pi\bar{\rho}$. The lower bound is,

$$\begin{aligned}
\mathrm{e}^{-\mu(\mathbb{S}^2)}\mathrm{e}^{\mu(\mathbb{S}^2)A_2(\mathbf{x})} &= \exp\left(-4\pi\bar{\rho}(1-\cos r) + \frac{\bar{\rho}^2}{\mu(\mathbb{S}^2)}\int_{\mathbb{S}^2\cap B_{\mathbb{S}^2}(\mathbf{x},r)} \frac{1}{\rho(\mathbf{y})}\lambda_{\mathbb{S}^2}(d\mathbf{y})\right) \\
&\geq \exp\left(-4\pi\bar{\rho}(1-\cos r)\right) \\
&\geq \exp\left(-8\pi\bar{\rho}\right).
\end{aligned}$$

Next we need to show that $A_2(\mathbf{x})$ is strictly greater than 0,

$$\begin{aligned}
A_2(\mathbf{x}) &= 1 - \frac{4\pi\bar{\rho}}{\mu(\mathbb{S}^2)}(1-\cos r) + \frac{\bar{\rho}^2}{\mu(\mathbb{S}^2)}\int_{\mathbb{S}^2\cap B_{\mathbb{S}^2}(\mathbf{x},r)} \frac{1}{\rho(\mathbf{y})}\lambda_{\mathbb{S}^2}(d\mathbf{y}) \\
&= \int_{\mathbb{S}^2}\left(1 - \frac{\bar{\rho}\mathbb{1}[\mathbf{y}\in B_{\mathbb{S}^2}(\mathbf{x},r)]}{\rho(\mathbf{y})}\right)^2 \frac{\rho(\mathbf{y})}{\mu(\mathbb{S}^2)}\lambda_{\mathbb{S}^2}(d\mathbf{y}) \\
&= \int_{\mathbb{S}^2\cap B_{\mathbb{S}^2}(\mathbf{x},r)}\left(1 - \frac{\bar{\rho}}{\rho(\mathbf{y})}\right)^2 \frac{\rho(\mathbf{y})}{\mu(\mathbb{S}^2)}\lambda_{\mathbb{S}^2}(d\mathbf{y}) + \int_{\mathbb{S}^2\setminus B_{\mathbb{S}^2}(\mathbf{x},r)} \frac{\rho(\mathbf{y})}{\mu(\mathbb{S}^2)}\lambda_{\mathbb{S}^2}(d\mathbf{y}).
\end{aligned}$$

Then for $r \in [0,\pi)$ $\mu_L(\mathbb{S}^2 \setminus B_{\mathbb{S}^2}(\mathbf{x},r)) > 0$ and since $\rho(\mathbf{y}) \geq \bar{\rho} > 0$ this means that the second term is strictly greater than 0. Further the first term is always non-negative since $\rho(\mathbf{y}) \geq \bar{\rho} > 0$. Then if $r = \pi$ we have that,

$$\begin{aligned}
A_2(\mathbf{x}) &= \int_{\mathbb{S}^2}\left(1 - \frac{\bar{\rho}}{\rho(\mathbf{y})}\right)^2 \frac{\rho(\mathbf{y})}{\mu(\mathbb{S}^2)}\lambda_{\mathbb{S}^2}(d\mathbf{y}) \\
&= \int_{\mathbb{S}^2\cap E}\left(1 - \frac{\bar{\rho}}{\rho(\mathbf{y})}\right)^2 \frac{\rho(\mathbf{y})}{\mu(\mathbb{S}^2)}\lambda_{\mathbb{S}^2}(d\mathbf{y}) + \int_{\mathbb{S}^2\setminus E}\left(1 - \frac{\bar{\rho}}{\rho(\mathbf{y})}\right)^2 \frac{\rho(\mathbf{y})}{\mu(\mathbb{S}^2)}\lambda_{\mathbb{S}^2}(d\mathbf{y}),
\end{aligned}$$

then by assumption $\rho(\mathbf{y}) > \bar{\rho}$ for all $\mathbf{y} \in E \subset \mathbb{S}^2$, hence the first term is strictly greater than 0 whilst the second term is non-negative and so we have shown that for any $r \in [0,\pi]$ $A_2(\mathbf{x}) > 0$, therefore when taking the reciprocal of $A_2(\mathbf{x})$ it is well defined for all $\mathbf{x} \in \mathbb{S}^2$. Then we can bound the absolute value of the reciprocal of $A_2(\mathbb{S}^2)$. Further, in the case when $r \in [0,\pi)$, $\int_{\mathbb{S}^2\setminus B_{\mathbb{S}^2}(\mathbf{x},r)} \frac{\rho(\mathbf{y})}{\mu(\mathbb{S}^2)}\lambda_{\mathbb{S}^2}(d\mathbf{y}) \geq \int_{\mathbb{S}^2\setminus B_{\mathbb{S}^2}(\mathbf{x},r)} \frac{\bar{\rho}}{\mu(\mathbb{S}^2)}\lambda_{\mathbb{S}^2}(d\mathbf{y}) = (1-\cos r)\frac{2\pi\bar{\rho}}{\mu(\mathbb{S}^2)}$ then define,

$$\bar{A}_2 = \begin{cases} (1-\cos r)\frac{2\pi\bar{\rho}}{\mu(\mathbb{S}^2)}, & r \in [0,\pi) \\ \int_{\mathbb{S}^2\setminus E}\left(1 - \frac{\bar{\rho}}{\rho(\mathbf{y})}\right)^2 \frac{\rho(\mathbf{y})}{\mu(\mathbb{S}^2)}\lambda_{\mathbb{S}^2}(d\mathbf{y}), & r = \pi, \end{cases}$$

which does not depend on $\mathbf{x}$. Then $A_2(\mathbf{x}) \geq \bar{A}_2 > 0$ and so $0 \leq \frac{1}{A_2(\mathbf{x})} \leq \frac{1}{\bar{A}_2}$, which means we can bound the the absolute value of the reciprocal of $A_2(\mathbb{S}^2)$. Further we can obtain a non-zero lower bound for $\frac{1}{A_2(\mathbf{x})}$ since $A_2(\mathbf{x}) = 1 - \frac{4\pi\bar{\rho}}{\mu(\mathbb{S}^2)}(1-\cos r) + \frac{\bar{\rho}^2}{\mu(\mathbb{S}^2)}\int_{\mathbb{S}^2\cap B_{\mathbb{S}^2}(\mathbf{x},r)} \frac{1}{\rho(\mathbf{y})}\lambda_{\mathbb{S}^2}(d\mathbf{y}) \leq$

$1 + \frac{\bar{\rho}^2}{\mu(\mathbb{S}^2)} \int_{\mathbb{S}^2} \frac{1}{\rho(\mathbf{y})} \lambda_{\mathbb{S}^2}(d\mathbf{y})$. Let us define $\tilde{A}_2 = 1 + \frac{\bar{\rho}^2}{\mu(\mathbb{S}^2)} \int_{\mathbb{S}^2} \frac{1}{\rho(\mathbf{y})} \lambda_{\mathbb{S}^2}(d\mathbf{y})$ then returning to Equation 20 we have,

$$\frac{1}{\mu(\mathbb{S}^2)\tilde{A}_2^2}\left(\mathrm{e}^{-8\pi\bar{\rho}} - \mathrm{e}^{-\mu(\mathbb{S}^2)}\right) \leq \frac{\mathrm{e}^{-\mu(\mathbb{S}^2)}}{A_2(\mathbf{x})}\left(\gamma + \log(\mu(\mathbb{S}^2)A_2(\mathbf{x})) - \mathrm{Ei}(\mu(\mathbb{S}^2)A_2(\mathbf{x}))\right) \leq \frac{\mathrm{e}}{\bar{A}_2},$$

and therefore,

$$\left|\frac{\mathrm{e}^{-\mu(\mathbb{S}^2)}}{A_2(\mathbf{x})}\left(\gamma + \log(\mu(\mathbb{S}^2)A_2(\mathbf{x})) - \mathrm{Ei}(\mu(\mathbb{S}^2)A_2(\mathbf{x}))\right)\right| \leq \max\left\{\left|\frac{1}{\mu(\mathbb{S}^2)\tilde{A}_2^2}\left(\mathrm{e}^{-8\pi\bar{\rho}} - \mathrm{e}^{-\mu(\mathbb{S}^2)}\right)\right|, \frac{\mathrm{e}}{\bar{A}_2}\right\}$$

Since the right hand side of this inequality is a constant this means that it is Lebesgue integrable over $\mathbb{S}^2$ and so by the dominated convergence theorem so is the left hand side, thus showing that the integrands are truly Lebesgue integrable. An identical approach can be used to show that the first term of Equation 19 is also Lebesgue integrable. □

S5. Existence and approximation of first and second order moments of the $\hat{J}_{\text{inhom}}$-function

In this section we discuss the existence of the first two moments of the empirical inhomogeneous J-function, outlining sufficient conditions for the moments to exist. We then derive approximations to the first and second order moments of $\hat{J}_{\text{inhom}}(r)$. The following theorem gives conditions under which the first two moments of $\hat{J}_{\text{inhom}}(r)$ exist.

S5.1. Existence of first and second moments of $J_{inhom(r)}$

Theorem 5. *Let X be a spheroidal Poisson process with intensity function $\rho : \mathbb{S}^2 \mapsto \mathbb{R}_+$ such that $\bar{\rho} \equiv \inf_{\mathbf{x}\in\mathbb{S}^2} \rho(\mathbf{x}) > 0$. Let P be any finite grid on $\mathbb{S}^2$ and define $r_{\max} = \sup\{r \in [0,\pi]$: there exists $\mathbf{p} \in P$ such that $\rho(\mathbf{x}) \neq \bar{\rho}$ for all $\mathbf{x} \in B_{\mathbb{S}^2}(\mathbf{p}, r)\}$. Then for any given $r \in [0, r_{\max}]$ both $\mathbb{E}[\hat{J}_{\text{inhom}}(r)]$ and $\text{Var}(\hat{J}_{\text{inhom}}(r))$ exist.*

Proof. Starting with the expectation,

$$\mathbb{E}[\hat{J}_{\text{inhom}}(r)] = \int_{N_{lf}} \hat{J}_{\text{inhom}}(r) P_X(dx),$$

where $P_X(X \in F), F \subseteq N_{lf}$ is the probability measure of X. Define $N_{lf,0} = \{x \in N_{lf} | n_x(\mathbb{S}^2) = 0\}$ and $N_{lf,1} = \{x \in N_{lf} | n_x(\mathbb{S}^2) > 0\}$, then $N_{lf} = N_{lf,0} \cup N_{lf,1}$ and $N_{lf,0} \cap N_{lf,1} = \emptyset$ and so,

$$\mathbb{E}[\hat{J}_{\text{inhom}}(r)] = \int_{N_{lf,0}} \hat{J}_{\text{inhom}}(r) P_X(dx) + \int_{N_{lf,1}} \hat{J}_{\text{inhom}}(r) P_X(dx),$$

taking the convention that $\frac{0}{0} = 1$, the first term is finite,

$$= \int_{N_{lf,0}} P_X(dx) + \int_{N_{lf,1}} \hat{J}_{\text{inhom}}(r) P_X(dx),$$
$$= P_X(X \in N_{lf,0}) + \int_{N_{lf,1}} \hat{J}_{\text{inhom}}(r) P_X(dx),$$

it can be shown that $P_X(X \in N_{lf,0}) = P_{N_X(\mathbb{S}^2)}(N_X(\mathbb{S}^2) = 0)$ then,

$$= P(N_X(\mathbb{S}^2) = 0) + \int_{N_{lf,1}} \hat{J}_{\text{inhom}}(r) P_X(dx).$$

We now show that the second term can be bounded and hence the expectation is well defined. Taking the integrand and noting that random variables are now deterministic,

$$\hat{J}_{\text{inhom}}(r) = \frac{|P| \sum_{\mathbf{x} \in x} \prod_{\mathbf{y} \in x \setminus \mathbf{x}} \left(1 - \frac{\bar{\rho}\mathbb{1}[\mathbf{y} \in B_{\mathbb{S}^2}(\mathbf{x}, r)]}{\rho(\mathbf{y})}\right)}{n_x(\mathbb{S}^2) \sum_{\mathbf{p} \in P} \prod_{\mathbf{z} \in x} \left(1 - \frac{\bar{\rho}\mathbb{1}[\mathbf{z} \in B_{\mathbb{S}^2}(\mathbf{p}, r)]}{\rho(\mathbf{z})}\right)}.$$

First we note that $\hat{J}_{\text{inhom}}(r) \geq 0$ and is so bounded below. Working with the numerator we have that,

$$\begin{aligned}\sum_{\mathbf{x} \in x} \prod_{\mathbf{y} \in x \setminus \mathbf{x}} \left(1 - \frac{\bar{\rho}\mathbb{1}[\mathbf{y} \in B_{\mathbb{S}^2}(\mathbf{x}, r)]}{\rho(\mathbf{y})}\right) &\leq \sum_{\mathbf{x} \in x} \prod_{y \in x \setminus \mathbf{x}} 1 \\ &= n_x(\mathbb{S}^2).\end{aligned}$$

Now working with the denominator showing that it is bounded below and hence its reciprocal bounded above. By the assumption we have that there exists $\tilde{\mathbf{p}} \in P$ such that for any $\mathbf{x} \in B_{\mathbb{S}^2}(\tilde{\mathbf{p}}, r), \rho(\mathbf{x}) \neq \bar{\rho}$, then

$$\begin{aligned}&\sum_{\mathbf{p} \in P} \prod_{\mathbf{z} \in x} \left(1 - \frac{\bar{\rho}\mathbb{1}[\mathbf{z} \in B_{\mathbb{S}^2}(\mathbf{p}, r)]}{\rho(\mathbf{z})}\right) = \\ &\qquad \prod_{\mathbf{z} \in x} \left(1 - \frac{\bar{\rho}\mathbb{1}[\mathbf{z} \in B_{\mathbb{S}^2}(\tilde{\mathbf{p}}, r)]}{\rho(\mathbf{z})}\right) + \sum_{\mathbf{p} \in P \setminus \tilde{p}} \prod_{\mathbf{z} \in x} \left(1 - \frac{\bar{\rho}\mathbb{1}[\mathbf{z} \in B_{\mathbb{S}^2}(\mathbf{p}, r)]}{\rho(\mathbf{z})}\right).\end{aligned}$$

Then the first term is strictly greater than 0 by our assumption. Thus,

$$\sum_{\mathbf{p} \in P} \prod_{\mathbf{z} \in x} \left(1 - \frac{\bar{\rho}\mathbb{1}[\mathbf{z} \in B_{\mathbb{S}^2}(\mathbf{p}, r)]}{\rho(\mathbf{z})}\right) \geq \prod_{\mathbf{z} \in x} \left(1 - \frac{\bar{\rho}\mathbb{1}[\mathbf{z} \in B_{\mathbb{S}^2}(\tilde{\mathbf{p}}, r)]}{\rho(\mathbf{z})}\right) > 0.$$

Further by the assumption we can define $\bar{\rho}_{\tilde{\mathbf{p}}} = \inf_{\mathbf{x} \in B_{\mathbb{S}^2}(\tilde{\mathbf{p}}, r)} \rho(\mathbf{x})$ and then,

$$\sum_{\mathbf{p} \in P} \prod_{\mathbf{z} \in x} \left(1 - \frac{\bar{\rho}\mathbb{1}[\mathbf{z} \in B_{\mathbb{S}^2}(\mathbf{p}, r)]}{\rho(\mathbf{z})}\right) \geq \prod_{\mathbf{z} \in x} \left(1 - \frac{\bar{\rho}}{\bar{\rho}_{\tilde{\mathbf{p}}}}\right) = \left(1 - \frac{\bar{\rho}}{\bar{\rho}_{\tilde{\mathbf{p}}}}\right)^{n_x(\mathbf{S}^2)},$$

and so,

$$\left|\hat{J}_{\text{inhom}}(r)\right| \leq \frac{|P| n_x(\mathbb{S}^2)}{n_x(\mathbb{S}^2) \left(1 - \frac{\bar{\rho}}{\bar{\rho}_{\tilde{\mathbf{p}}}}\right)^{n_x(\mathbf{S}^2)}} = |P| \left(1 - \frac{\bar{\rho}}{\bar{\rho}_{\tilde{\mathbf{p}}}}\right)^{-n_x(\mathbf{S}^2)}.$$

We now show that the right hand side of the above inequality is integrable. Define the sets $N_{lf,1,(i)} = \{x \in N_{lf,1} : n(x) = i\}$, then $N_{lf,1} = \bigcup_{i=1}^{\infty} N_{lf,1,(i)}$ and $N_{lf,1,(i)} \cap N_{lf,1,(j)} = \emptyset$ for $i \neq j$ and hence we have partitioned the space $N_{lf,1}$. Thus,

$$\int_{N_{lf,1}} |P| \left(1 - \frac{\bar{\rho}}{\bar{\rho}_{\tilde{\mathbf{p}}}}\right)^{-n_x(\mathbf{S}^2)} P_X(dx) = \sum_{i=1}^{\infty} \int_{N_{lf,1,(i)}} |P| \left(1 - \frac{\bar{\rho}}{\bar{\rho}_{\tilde{\mathbf{p}}}}\right)^{-n_x(\mathbb{S}^2)} P_X(dx)$$

$n_x(\mathbb{S}^2) = i$ for all $x \in N_{lf,1,(i)}$ and so is constant over each partition of the space,

$$\begin{aligned} &= \sum_{i=1}^{\infty} |P| \left(1 - \frac{\bar{\rho}}{\bar{\rho}_{\tilde{\mathbf{p}}}}\right)^{-i} \int_{N_{lf,1,(i)}} P_X(dx) \\ &= \sum_{i=1}^{\infty} |P| \left(1 - \frac{\bar{\rho}}{\bar{\rho}_{\tilde{\mathbf{p}}}}\right)^{-i} P_X(X \in N_{lf,1,(i)}) \end{aligned}$$

but it is easy to see that $P_X(X \in N_{lf,1,(i)}) = P_{N_X(\mathbb{S}^2)}(N_X(\mathbb{S}^2) = i)$, and since X is Poisson and defining $a = (1 - \frac{\bar{\rho}}{\bar{\rho}_{\tilde{\mathbf{p}}}})$ and $\lambda = \mu(\mathbb{S}^2)$,

$$\begin{aligned} &= |P| \sum_{i=1}^{\infty} a^{-i} \frac{\lambda^n \mathrm{e}^{-\lambda}}{i!} \\ &= |P| \mathrm{e}^{-\lambda} \sum_{i=1}^{\infty} \frac{(\lambda/a)^i}{i!} \\ &= |P| \mathrm{e}^{-\lambda} \left(\sum_{i=0}^{\infty} \frac{(\lambda/a)^i}{i!} - 1 \right) \\ &= |P| \mathrm{e}^{-\lambda} \left(\mathrm{e}^{-\lambda/a} - 1 \right). \end{aligned}$$

Hence we have shown that,

$$\int_{N_{lf,1}} \left| \hat{J}_{\mathrm{inhom}}(r) \right| P_X(dx) \leq |P| \mathrm{e}^{-\lambda} \left(\mathrm{e}^{-\lambda/a} - 1 \right)$$

and so by the dominated convergence theorem the expectation of $\hat{J}_{\mathrm{inhom}}(r)$ exists.

Existence of the variance of $\hat{J}_{\mathrm{inhom}}(r)$ follows simply based on the existence of the expectation,

$$\mathrm{Var}(\hat{J}_{\mathrm{inhom}}(r)) = \int_{N_{lf}} \left(\hat{J}_{\mathrm{inhom}}(r) - \mathbb{E}[\hat{J}_{\mathrm{inhom}}(r)] \right)^2 P_X(dx).$$

Partitioning the space N_{lf} again into $N_{lf,0} \equiv \{x \in N_{lf} | N(x) = 0\}$ and $N_{lf,1} \equiv \{x \in N_{lf} | N(x) > 0\}$ we have that,

$$\text{Var}(\hat{J}_{\text{inhom}}(r)) \tag{21}$$

$$= \int_{N_{lf,0}} \left(\hat{J}_{\text{inhom}}(r) - \mathbb{E}[\hat{J}_{\text{inhom}}(r)]\right)^2 P_X(dx) + \int_{N_{lf,1}} \left(\hat{J}_{\text{inhom}}(r) - \mathbb{E}[\hat{J}_{\text{inhom}}(r)]\right)^2 P_X(dx). \tag{22}$$

Taking the convention that $\frac{0}{0} = 1$ then the first term is simply,

$$\int_{N_{lf,0}} \left(\hat{J}_{\text{inhom}}(r) - \mathbb{E}[\hat{J}_{\text{inhom}}(r)]\right)^2 P_X(dx) = \mathbb{E}^2[\hat{J}_{\text{inhom}}(r)] \int_{N_{lf,0}} P_X(dx)$$
$$= \mathbb{E}^2[\hat{J}_{\text{inhom}}(r)] P_X(X \in N_{lf,0}),$$

then we note that $P_X(X \in N_{lf,0}) = P(N_X(\mathbb{S}^2) = 0)$ and so,

$$= \mathbb{E}^2[\hat{J}_{\text{inhom}}(r)] P(N_X(\mathbb{S}^2) = 0),$$

which is finite since the expectation exists. The second term of Equation 22 over the space $N_{lf,1}$ can also be shown to be finite. Since $\mathbb{E}[\hat{J}_{\text{inhom}}(r)]$ is finite we just need to show that $\hat{J}_{\text{inhom}}(r)$ is bounded in order show that the integrand is bounded and hence integrable. But from proving the expectation exists we have that,

$$0 \leq \hat{J}_{\text{inhom}}(r) \leq |P| \left(1 - \frac{\bar{\rho}}{\bar{\rho}_{\tilde{\mathbf{P}}}}\right)^{-n_x(\mathbf{S}^2)},$$

and so the integrand of second term in Equation 22 is bounded and thus the variance exists. □

Remark S1. *From this theorem the requirement of the process being Poisson was only needed for a closed form of the distribution for its corresponding counting process* $N_X(\mathbb{S}^2)$*. We can drop the requirement of* X *being Poisson but instead require that the probability generating function of* $N_X(\mathbb{S}^2)$*,* $G_{N_X(\mathbb{S}^2)}(s)$*, has radius of convergence* $|s| \leq \left(1 - \frac{\bar{\rho}}{\bar{\rho}_{\tilde{\mathbf{P}}}}\right)^{-1}$*. This condition would be sufficient for the theorem to hold true.*

S5.2. Covariance between $\hat{H}_{inhom}(r)$ and $\hat{F}_{inhom}(r)$

Proposition 1. *Let X be a spheroidal Poisson process with known intensity function $\rho : \mathbb{S}^2 \mapsto \mathbb{R}$. Then the covariance between $1 - \hat{H}_{inhom}(r)$ and $1 - \hat{F}_{inhom}(r)$ for $r \in [0, \pi]$ is,*

$$
\begin{aligned}
&Cov(1 - \hat{H}_{inhom}(r), 1 - \hat{F}_{inhom}(r)) \\
&= \frac{1}{|P|} \sum_{\mathbf{p} \in P} \int_{\mathbb{S}^2} \left(1 - \frac{\bar{\rho} \mathbb{1}[\mathbf{x} \in B_{\mathbb{S}^2}(\mathbf{p}, r)]}{\rho(\mathbf{x})}\right) \\
&\quad \frac{\exp\left\{-2\bar{\rho} 2\pi(1 - \cos r) - \int_{B_{\mathbb{S}^2}(\mathbf{x}, r) \cap B_{\mathbb{S}^2}(\mathbf{p}, r)} \frac{\bar{\rho}^2}{\rho(\mathbf{y})} \lambda_{\mathbb{S}^2}(d\mathbf{y})\right\}}{A(\mathbf{x}, \mathbf{p})} \frac{\rho(\mathbf{x})}{\mu(\mathbb{S}^2)} \lambda_{\mathbb{S}^2}(d\mathbf{x}) \\
&\quad - \exp(-2\pi(1 - \cos r)\bar{\rho}) \left(\exp(-2\pi(1 - \cos r)\bar{\rho}) - \exp(-\mu(\mathbb{S}^2))\right) \frac{\mu(\mathbb{S}^2)}{\mu(\mathbb{S}^2) - 2\pi(1 - \cos r)\bar{\rho}},
\end{aligned}
$$

where P is a finite grid of points on $\mathbb{S}^2$ and,

$$
A(\mathbf{x}, \mathbf{p}) = 1 - \frac{2\bar{\rho}}{\mu(\mathbb{S}^2)} 2\pi(1 - \cos r) + \frac{1}{\mu(\mathbb{S}^2)} \int_{B_{\mathbb{S}^2}(\mathbf{x}, r) \cap B_{\mathbb{S}^2}(\mathbf{p}, r)} \frac{\bar{\rho}^2}{\rho(\mathbf{y})} \lambda_{\mathbb{S}^2}(d\mathbf{y}).
$$

Proof. Define,

$$
\begin{aligned}
X &\equiv 1 - \hat{H}_{\text{inhom}}(r) = \frac{1}{N_X(\mathbb{S}^2)} \sum_{\mathbf{x} \in X} \prod_{\mathbf{y} \in X \setminus \{\mathbf{x}\}} \left(1 - \frac{\bar{\rho} \mathbb{1}[\mathbf{y} \in B_{\mathbb{S}^2}(\mathbf{x}, r)]}{\rho(\mathbf{y})}\right) \\
Y &\equiv 1 - \hat{F}_{\text{inhom}}(r) = \frac{1}{|P|} \sum_{\mathbf{p} \in P} \prod_{\mathbf{y} \in X} \left(1 - \frac{\bar{\rho} \mathbb{1}[\mathbf{y} \in B_{\mathbb{S}^2}(\mathbf{p}, r)]}{\rho(\mathbf{y})}\right)
\end{aligned}
$$

Then,

$$
\text{Cov}(X, Y) = \underbrace{\mathbb{E}[XY]}_{(a)} - \underbrace{\mathbb{E}[X]\mathbb{E}[Y]}_{(b)}
$$

Term (a) is given by,

$$
\begin{aligned}
&\mathbb{E}[XY] \\
&= \mathbb{E}\left[\left(\frac{1}{N_X(\mathbb{S}^2)} \sum_{\mathbf{x} \in X} \prod_{\mathbf{y} \in X \setminus \{\mathbf{x}\}} \left(1 - \frac{\bar{\rho} \mathbb{1}[\mathbf{y} \in B_{\mathbb{S}^2}(\mathbf{x}, r)]}{\rho(\mathbf{y})}\right)\right) \left(\frac{1}{|P|} \sum_{\mathbf{p} \in P} \prod_{\mathbf{y} \in X} \left(1 - \frac{\bar{\rho} \mathbb{1}[\mathbf{y} \in B_{\mathbb{S}^2}(\mathbf{p}, r)]}{\rho(\mathbf{y})}\right)\right)\right] \\
&= \frac{1}{|P|} \sum_{\mathbf{p} \in P} \mathbb{E}\left[\frac{1}{N_X(\mathbb{S}^2)} \sum_{\mathbf{x} \in X} \left(\prod_{\mathbf{y} \in X \setminus \{\mathbf{x}\}} \left(1 - \frac{\bar{\rho} \mathbb{1}[\mathbf{y} \in B_{\mathbb{S}^2}(\mathbf{x}, r)]}{\rho(\mathbf{y})}\right)\right) \left(\prod_{\mathbf{y} \in X} \left(1 - \frac{\bar{\rho} \mathbb{1}[\mathbf{y} \in B_{\mathbb{S}^2}(\mathbf{p}, r)]}{\rho(\mathbf{y})}\right)\right)\right] \\
&= \frac{1}{|P|} \sum_{\mathbf{p} \in P} \mathbb{E}\left(\frac{1}{N_X(\mathbb{S}^2)} \sum_{i=1}^{N_X(\mathbb{S}^2)}\right.
\end{aligned}
$$

$$\underbrace{\mathbb{E}\left[\left(\prod_{\substack{j=1\\j\neq i}}^{n}\left(1-\frac{\bar{\rho}\mathbb{1}[\mathbf{X}_j\in B_{\mathbb{S}^2}(\mathbf{X}_i,r)]}{\rho(\mathbf{X}_j)}\right)\right)\left(\prod_{j=1}^{n}\left(1-\frac{\bar{\rho}\mathbb{1}[\mathbf{X}_j\in B_{\mathbb{S}^2}(\mathbf{p},r)]}{\rho(\mathbf{X}_j)}\right)\right)\middle| N_X(\mathbb{S}^2)=n\right]}_{(\star)}\Bigg),$$

where $X_k,\ k=1,\dots,n$ are independently distributed on $\mathbb{S}^2$ with density proportional to $\rho(\mathbf{x}_k)$, when conditioned on $N_X(\mathbb{S}^2)$. Taking $(\star)$,

$$\begin{aligned}
&\mathbb{E}\left[\left(\prod_{\substack{j=1\\j\neq i}}^{n}\left(1-\frac{\bar{\rho}\mathbb{1}[\mathbf{X}_j\in B_{\mathbb{S}^2}(\mathbf{X}_i,r)]}{\rho(\mathbf{X}_j)}\right)\right)\left(\prod_{j=1}^{n}\left(1-\frac{\bar{\rho}\mathbb{1}[\mathbf{X}_j\in B_{\mathbb{S}^2}(\mathbf{p},r)]}{\rho(\mathbf{X}_j)}\right)\right)\middle| N_X(\mathbb{S}^2)=n\right]\\
&=\mathbb{E}\left[\left(1-\frac{\bar{\rho}\mathbb{1}[\mathbf{X}_i\in B_{\mathbb{S}^2}(\mathbf{p},r)]}{\rho(\mathbf{X}_i)}\right)\left(\prod_{\substack{j=1\\j\neq i}}^{n}\left(1-\frac{\bar{\rho}\mathbb{1}[\mathbf{X}_j\in B_{\mathbb{S}^2}(\mathbf{X}_i,r)]}{\rho(\mathbf{X}_j)}\right)\left(1-\frac{\bar{\rho}\mathbb{1}[\mathbf{X}_j\in B_{\mathbb{S}^2}(\mathbf{p},r)]}{\rho(\mathbf{X}_j)}\right)\right)\right],
\end{aligned}$$

Using iterated expectations and conditioning on $\mathbf{X}_i$,

$$\begin{aligned}
&=\mathbb{E}\left[\left(1-\frac{\bar{\rho}\mathbb{1}[\mathbf{X}_i\in B_{\mathbb{S}^2}(\mathbf{p},r)]}{\rho(\mathbf{X}_i)}\right)\prod_{\substack{j=1\\j\neq i}}^{n}\mathbb{E}\left[\left(1-\frac{\bar{\rho}\mathbb{1}[\mathbf{X}_j\in B_{\mathbb{S}^2}(\mathbf{x},r)]}{\rho(\mathbf{X}_j)}\right)\left(1-\frac{\bar{\rho}\mathbb{1}[\mathbf{X}_j\in B_{\mathbb{S}^2}(\mathbf{p},r)]}{\rho(\mathbf{X}_j)}\right)\right]\right]\\
&=\mathbb{E}\left[\left(1-\frac{\bar{\rho}\mathbb{1}[\mathbf{X}_i\in B_{\mathbb{S}^2}(\mathbf{p},r)]}{\rho(\mathbf{X}_i)}\right)\mathbb{E}^{n-1}\left[\left(1-\frac{\bar{\rho}\mathbb{1}[\mathbf{Y}\in B_{\mathbb{S}^2}(\mathbf{x},r)]}{\rho(\mathbf{Y})}\right)\left(1-\frac{\bar{\rho}\mathbb{1}[\mathbf{Y}\in B_{\mathbb{S}^2}(\mathbf{p},r)]}{\rho(\mathbf{Y})}\right)\right]\right]
\end{aligned}\tag{23}$$

where $\mathbf{Y}$ is distributed with density proportional to $\rho(\mathbf{y})$ on $\mathbb{S}^2$. It is then easy to show that,

$$\begin{aligned}
&\mathbb{E}\left[\left(1-\frac{\bar{\rho}\mathbb{1}[\mathbf{Y}\in B_{\mathbb{S}^2}(\mathbf{x},r)]}{\rho(\mathbf{Y})}\right)\left(1-\frac{\bar{\rho}\mathbb{1}[\mathbf{Y}\in B_{\mathbb{S}^2}(\mathbf{p},r)]}{\rho(\mathbf{Y})}\right)\right]\\
&\qquad=1-\frac{2\bar{\rho}}{\mu(\mathbb{S}^2)}2\pi(1-\cos r)+\frac{1}{\mu(\mathbb{S}^2)}\int_{B_{\mathbb{S}^2}(\mathbf{x},r)\cap B_{\mathbb{S}^2}(\mathbf{p},r)}\frac{\bar{\rho}^2}{\rho(\mathbf{y})}\lambda_{\mathbb{S}^2}(d\mathbf{y}).
\end{aligned}$$

Let us define $A(\mathbf{x},\mathbf{p})=\mathbb{E}\left[\left(1-\frac{\bar{\rho}\mathbb{1}[\mathbf{Y}\in B_{\mathbb{S}^2}(\mathbf{x},r)]}{\rho(\mathbf{Y})}\right)\left(1-\frac{\bar{\rho}\mathbb{1}[\mathbf{Y}\in B_{\mathbb{S}^2}(\mathbf{p},r)]}{\rho(\mathbf{Y})}\right)\right]$, and so returning to 23,

$$\mathbb{E}\left[\left(1-\frac{\bar{\rho}\mathbb{1}[\mathbf{X}_i\in B_{\mathbb{S}^2}(\mathbf{p},r)]}{\rho(\mathbf{X}_i)}\right)A^{n-1}(\mathbf{X}_i,\mathbf{p})\right]=\mathbb{E}\left[\left(1-\frac{\bar{\rho}\mathbb{1}[\mathbf{X}\in B_{\mathbb{S}^2}(\mathbf{p},r)]}{\rho(\mathbf{X})}\right)A^{n-1}(\mathbf{X},\mathbf{p})\right],$$

where $\mathbf{X}$ has density proportional to $\rho(\mathbf{x})$ on $\mathbb{S}^2$,

$$= \int_{\mathbb{S}^2} \left(1 - \frac{\bar{\rho}\mathbb{1}[\mathbf{x} \in B_{\mathbb{S}^2}(\mathbf{p}, r)]}{\rho(\mathbf{x})}\right) A^{n-1}(\mathbf{x}, \mathbf{p}) \frac{\rho(\mathbf{x})}{\mu(\mathbb{S}^2)} \lambda_{\mathbb{S}^2}(d\mathbf{x}).$$

And so,

$$\begin{aligned}
\mathbb{E}[XY] &= \frac{1}{|P|} \sum_{\mathbf{p} \in P} \mathbb{E}\left[\frac{1}{N_X(\mathbb{S}^2)} \sum_{i=1}^{N_X(\mathbb{S}^2)} \int_{\mathbb{S}^2} \left(1 - \frac{\bar{\rho}\mathbb{1}[\mathbf{x} \in B_{\mathbb{S}^2}(\mathbf{p}, r)]}{\rho(\mathbf{x})}\right) A^{N_X(\mathbb{S}^2)-1}(\mathbf{x}, \mathbf{p}) \frac{\rho(\mathbf{x})}{\mu(\mathbb{S}^2)} \lambda_{\mathbb{S}^2}(d\mathbf{x})\right] \\
&= \frac{1}{|P|} \sum_{\mathbf{p} \in P} \mathbb{E}\left[\int_{\mathbb{S}^2} \left(1 - \frac{\bar{\rho}\mathbb{1}[\mathbf{x} \in B_{\mathbb{S}^2}(\mathbf{p}, r)]}{\rho(\mathbf{x})}\right) A^{N_X(\mathbb{S}^2)-1}(\mathbf{x}, \mathbf{p}) \frac{\rho(\mathbf{x})}{\mu(\mathbb{S}^2)} \lambda_{\mathbb{S}^2}(d\mathbf{x})\right] \\
&= \frac{1}{|P|} \sum_{\mathbf{p} \in P} \int_{\mathbb{S}^2} \left(1 - \frac{\bar{\rho}\mathbb{1}[\mathbf{x} \in B_{\mathbb{S}^2}(\mathbf{p}, r)]}{\rho(\mathbf{x})}\right) \mathbb{E}\left[A^{N_X(\mathbb{S}^2)-1}(\mathbf{x}, \mathbf{p})\right] \frac{\rho(\mathbf{x})}{\mu(\mathbb{S}^2)} \lambda_{\mathbb{S}^2}(d\mathbf{x})
\end{aligned}$$

Then it can easily be shown that,

$$\mathbb{E}\left[A^{N_X(\mathbb{S}^2)-1}(\mathbf{x}, \mathbf{p})\right] = \frac{\exp\left\{-2\bar{\rho}2\pi(1 - \cos r) - \int_{B_{\mathbb{S}^2}(\mathbf{x}, r) \cap B_{\mathbb{S}^2}(\mathbf{p}, r)} \frac{\bar{\rho}^2}{\rho(\mathbf{y})} \lambda_{\mathbb{S}^2}(d\mathbf{y})\right\}}{A(\mathbf{x}, \mathbf{p})}.$$

Then from Theorem 3 we have that,

$$\begin{aligned}
\mathbb{E}[X] &= \exp(2\pi(1 - \cos r)\bar{\rho}) \\
\mathbb{E}[Y] &= \mu^2(\mathbb{S}) \frac{\exp(2\pi(1 - \cos r)\bar{\rho}) - \exp(\mu(\mathbb{S}^2))}{\mu(\mathbb{S}^2) - 2\pi(1 - \cos r)\bar{\rho}}.
\end{aligned}$$

And so we have the covariance between $\hat{H}_{\text{inhom}}(r)$ and $\hat{F}_{\text{inhom}}(r)$. □

S5.3. Taylor Series Expansion

We discuss Taylor series expansions in general and their use to approximate moments of random variables [8], after which we apply this to the $\hat{J}_{\text{inhom}}$-function. For any function $f : \mathbb{R}^2 \mapsto \mathbb{R}$, we have its Taylor expansion up to second order around $\boldsymbol{\theta} = (\theta_x, \theta_y)$ as,

$$\begin{aligned}
f(x, y) = f(\theta_x, \theta_y) &+ \left.\frac{\partial f}{\partial x}\right|_{\theta_X, \theta_Y} (x - \theta_x) + \left.\frac{\partial f}{\partial y}\right|_{\theta_X, \theta_Y} (y - \theta_y) + \\
&\frac{1}{2}\left[\left.\frac{\partial^2 f}{\partial x^2}\right|_{\theta_X, \theta_Y} (x - \theta_x)^2 + 2\left.\frac{\partial^2 f}{\partial x \partial y}\right|_{\theta_X, \theta_Y} (x - \theta_x)(y - \theta_x) + \left.\frac{\partial^2 f}{\partial y^2}\right|_{\theta_X, \theta_Y} (y - \theta_y)^2\right] + R(x, y),
\end{aligned}$$

where $R(x,y)$ is a remainder term. Thus using random variables X and Y, with $\boldsymbol{\theta} = (\mathbb{E}[X], \mathbb{E}[Y]) \equiv (\mu_X, \mu_Y)$, whilst also assuming that $\mathbb{E}[R(X,Y)]$ is close to 0 then,

$$\mathbb{E}[f(X,Y)] \approx f(\mu_X, \mu_Y) + \frac{1}{2}\left[\frac{\partial^2 f}{\partial x^2}\text{Var}(X) + 2\frac{\partial^2 f}{\partial x \partial y}\text{Cov}(X,Y) + \frac{\partial^2 f}{\partial y^2}\text{Var}(Y)\right].$$

Then to approximate the variance we first note that using the first order Taylor series expansion $\mathbb{E}[f(X,Y)] \approx f(\mu_X, \mu_Y)$. Then,

$$\begin{aligned}
\text{Var}(f(X,Y)) &= \mathbb{E}[(f(X,Y) - \mathbb{E}[f(X,Y)])^2] \\
&\approx \mathbb{E}[(f(X,Y) - f(\mu_X, \mu_Y))^2] \\
&\approx \mathbb{E}\left[\left(f(\mu_X, \mu_Y) + \left.\frac{\partial f}{\partial x}\right|_{\mu_X, \mu_Y}(X - \mu_X) + \left.\frac{\partial f}{\partial y}\right|_{\mu_X, \mu_Y}(Y - \mu_Y) - f(\mu_X, \mu_Y)\right)^2\right] \\
&= \mathbb{E}\left[\left(\left.\frac{\partial f}{\partial x}\right|_{\mu_X, \mu_Y}(X - \mu_X) + \left.\frac{\partial f}{\partial y}\right|_{\mu_X, \mu_Y}(Y - \mu_Y)\right)^2\right] \\
&= \left.\frac{\partial f}{\partial x}\right|^2_{\mu_X, \mu_Y}\text{Var}(X) + 2\left.\frac{\partial f}{\partial x}\right|_{\mu_X, \mu_Y}\left.\frac{\partial f}{\partial y}\right|_{\mu_X, \mu_Y}\text{Cov}(X,Y) + \left.\frac{\partial f}{\partial y}\right|^2_{\mu_X, \mu_Y}\text{Var}(Y)
\end{aligned}$$

Hence defining $f(X,Y) = \frac{X}{Y}$, we have the following approximations to the first and second order moments of $f(X,Y)$,

$$\mathbb{E}\left[\frac{X}{Y}\right] \approx \frac{\mu_X}{\mu_Y} - \frac{\text{Cov}(X,Y)}{\mu_Y^2} + \frac{\text{Var}(Y)\mu_X}{\mu_Y^3} \tag{24}$$

$$\text{Var}\left(\frac{X}{Y}\right) \approx \frac{\mu_X}{\mu_Y}\left[\frac{\text{Var}(X)}{\mu_X^2} - 2\frac{\text{Cov}(X,Y)}{\mu_X \mu_Y} + \frac{\text{Var}(Y)}{\mu_Y^2}\right]. \tag{25}$$

Then since $\hat{J}_{\text{inhom}}(r)$ is defined as the ratio of two random variables, in particular $1 - \hat{H}_{\text{inhom}}(r)$ and $1 - \hat{F}_{\text{inhom}}(r)$. Thus combining Proposition 1 with the Taylor series expansions given in Equations 24 and 25 provides an estimate for the expectation and variance of $\hat{J}_{\text{inhom}}(r)$.

S6. Regular and Cluster Processes

In this section we discuss properties of the regular and cluster processes discussed in the paper. These properties help to design the simulations discussed in the paper. We also discuss why we observe decreasing power of our test statistic as $\mathbb{D}$ *deforms* further away from the unit sphere.

S6.1. Properties of Regular and Cluster Processes

The following proposition gives the expected number of points for Matérn I and II processes in any subset of $\mathbb{D}$.

Proposition S1. *Let X_1 and X_2 be a Matérn I and II inhibition processes respectively. In the Matérn II case we also define a mark distribution $P_{M_{\mathbf{x}}}$ such that the mark is independent not only of all other points $\mathbf{y} \in X_2 \setminus \{\mathbf{x}\}$ and marks $M_{\mathbf{y}}$, for $\mathbf{y} \in X_2 \setminus \{\mathbf{x}\}$ but also of $\mathbf{x}$, the point associated to the mark $M_{\mathbf{x}}$. Define N_1 and N_2 to be the random counting measures of X_1 and X_2. Then the expectations of N_{X_1} and N_{X_2} are given as,*

$$\mathbb{E}[N_{X_1}(B)] = \rho \int_B \mathrm{e}^{-\rho\lambda_{\mathbb{D}}(B_{\mathbb{D}}(\mathbf{x},R))}\lambda_{\mathbb{D}}(d\mathbf{x})$$
$$\mathbb{E}[N_{X_2}(B)] = \int_B \frac{1-\mathrm{e}^{-\rho\lambda_{\mathbb{D}}(B_{\mathbb{D}}(\mathbf{x},R))}}{\lambda_{\mathbb{D}}(B_{\mathbb{D}}(\mathbf{x},R))}\lambda_{\mathbb{D}}(d\mathbf{x}),$$

where $B \subseteq \mathbb{D}$.

Proof. Let us first start with X_1. Define $Y_1 \sim PPP(\rho, \mathbb{D})$ to be the homogeneous Poisson process which is thinned to give X_1. Then $\forall B \subseteq \mathbb{D}$ we can rewrite the counting measure for X_1 as,

$$N_{X_1}(B) = \sum_{\mathbf{x}\in Y_1\cap B} \mathbb{1}[N_{Y_1\setminus\{\mathbf{x}\}}(B_{\mathbb{D}}(\mathbf{x},R)) = 0],$$

where $N_{Y_1\setminus\{\mathbf{x}\}}$, is the random counting measure for the process Y_1 without the point $\mathbf{x}$. Then taking expectations and using the Slivnyak-Mecke Theorem,

$$\mathbb{E}[N_{X_1}(B)] = \mathbb{E}\left[\sum_{\mathbf{x}\in Y_1\cap B} \mathbb{1}[N_{Y_1\setminus\{\mathbf{x}\}}(B_{\mathbb{D}}(\mathbf{x},R)) = 0]\right]$$

$$= \int_B \mathbb{E}\left[\mathbb{1}[N_{Y_1}(B_{\mathbb{D}}(\mathbf{x},R)) = 0]\right] \rho\lambda_{\mathbb{D}}(d\mathbf{x})$$
$$= \rho \int_B \mathbb{P}(N_{Y_1}(B_{\mathbb{D}}(\mathbf{x},R)) = 0)\lambda_{\mathbb{D}}(d\mathbf{x})$$
$$= \rho \int_B \mathrm{e}^{-\rho\lambda_{\mathbb{D}}(B_{\mathbb{D}}(\mathbf{x},R))}\lambda_{\mathbb{D}}(d\mathbf{x})$$

For $N_{X_2}(B)$ a few more steps are required in order to take into account the mark associated with each point. Similarly to the counting measure for X_1, we can rewrite the counting measure for X_2 as,

$$N_{X_2}(B) = \sum_{\mathbf{x}\in Y_2\cap B} \mathbb{1}[M_{\mathbf{x}} \le M_{\mathbf{y}}, \forall \mathbf{y} \in (Y_2 \setminus \{\mathbf{x}\}) \cap B_{\mathbb{D}}(\mathbf{x},R)].$$

By again taking expectations and using the Slivnyak-Mecke Theorem,

$$\mathbb{E}[N_{X_2}(B)] = \mathbb{E}\left[\sum_{\mathbf{x}\in Y_2\cap B} \mathbb{1}[M_{\mathbf{x}} \le M_{\mathbf{y}}, \forall \mathbf{y} \in (Y_2 \setminus \{\mathbf{x}\}) \cap B_{\mathbb{D}}(\mathbf{x},R)]\right]$$
$$= \int_B \mathbb{E}[\mathbb{1}[M_{\mathbf{x}} \le M_{\mathbf{y}}, \forall \mathbf{y} \in Y_2 \cap B_{\mathbb{D}}(\mathbf{x},R)]]\rho\lambda_{\mathbb{D}}(d\mathbf{x})$$
$$= \int_B \mathbb{P}(M_{\mathbf{x}} \le M_{\mathbf{y}}, \forall \mathbf{y} \in Y_2 \cap B_{\mathbb{D}}(\mathbf{x},R))\rho\lambda_{\mathbb{D}}(d\mathbf{x})$$

Define $\lambda_{\mathbf{x}} = \rho\lambda_{\mathbb{D}}(B_{\mathbb{D}}(\mathbf{x},R))$, then the probability is calculated as follows,

$$\mathbb{P}(M_{\mathbf{x}} \le M_{\mathbf{y}}, \forall \mathbf{y} \in Y_2 \cap B_{\mathbb{D}}(\mathbf{x},R))$$
$$= \sum_{n=0}^{\infty} \mathbb{P}\left(M_{\mathbf{x}} \le M_{\mathbf{y}}, \forall \mathbf{y} \in Y_2 \cap B_{\mathbb{D}}(\mathbf{x},R) | N_{Y_2}(B_{\mathbb{D}}(\mathbf{x},R)) = n\right) \mathbb{P}(N_{Y_2}(B_{\mathbb{D}}(\mathbf{x},R)) = n)$$
$$= \sum_{n=0}^{\infty} \frac{\lambda_{\mathbf{x}}^n \mathrm{e}^{-\lambda_{\mathbf{x}}}}{n!} \mathbb{P}\left(M_{\mathbf{x}} \le M_{\mathbf{y}}, \forall \mathbf{y} \in Y_2 \cap B_{\mathbb{D}}(\mathbf{x},R) | N_{Y_2}(B_{\mathbb{D}}(\mathbf{x},R)) = n\right)$$

Let us label the points $\mathbf{y}_i \in Y_2$, for $i = 1, \ldots, n$ to be the n points in $B_{\mathbb{D}}(\mathbf{x},R)$ coming from the process Y_2. Then the event $M_{\mathbf{x}} \le M_{\mathbf{y}}, \forall \mathbf{y} \in Y_2 \cap B_{\mathbb{D}}(\mathbf{x},R)$ given that $N_{Y_2}(B_{\mathbb{D}}(\mathbf{x},R)) = n$ is identical to the event that the mark associated to the point $\mathbf{x}$ is the smallest of all marks. In other words we are concerned with the event $M_{\mathbf{x}} \le \min\{M_{\mathbf{y}_1}, \ldots, M_{\mathbf{y}_n}\}$. Let us define $M_{\min} = \min\{M_{\mathbf{y}_1}, \ldots, M_{\mathbf{y}_n}\}$ and $f_{M^{\min}}(m)$ to be the density function of $M_{\min}$, then by using order statistics we know that $f_{M^{\min}}(m) = n f_{M_{\mathbf{x}}}(m)(1 - F_{M_{\mathbf{x}}}(m))^{n-1}$, where

$f_{M_{\mathbf{x}}}(m)$ and $F_{M_{\mathbf{x}}}(m)$ are the density and cumulative density functions of an individual markis respectively. Then we have,

$$\begin{aligned}
&\mathbb{P}\left(M_{\mathbf{x}} \leq M_{\mathbf{y}}, \forall \mathbf{y} \in Y_2 \cap B_{\mathbb{D}}(\mathbf{x}, R) | N_{Y_2}(B_{\mathbb{D}}(\mathbf{x}, R)) = n\right) \\
&\quad = \mathbb{P}(M_{\mathbf{x}} \leq M^{\min}) \\
&\quad = \int_{\mathbb{M}^{\min}} \mathbb{P}(M_{\mathbf{x}} \leq m) f_{M^{\min}}(m) dm \\
&\quad = \int_{\mathbb{M}^{\min}} F_{M_{\mathbf{x}}}(m) n f_{M_{\mathbf{x}}}(m)(1 - F_{M_{\mathbf{x}}}(m))^{n-1} dm \\
&\quad = \left[F_{M_{\mathbf{x}}}(m)(1 - F_{M_{\mathbf{x}}}(m))^n\right]^{\mathbb{M}^{\min}} + \int_{\mathbb{M}^{\min}} f_{M_{\mathbf{x}}}(m)(1 - F_{M_{\mathbf{x}}}(m))^n dm \\
&\quad = \left[F_{M_{\mathbf{x}}}(m)(1 - F_{M_{\mathbf{x}}}(m))^n\right]^{\mathbb{M}^{\min}} + \left[\frac{1}{n+1}(1 - F_{M_{\mathbf{x}}}(m))^{n+1}\right]^{\mathbb{M}^{\min}} \\
&\quad = \frac{1}{n+1},
\end{aligned}$$

where the last line follows since the range of $\mathbb{M}^{\min}$ is identical to the range of $M_{\mathbf{x}}$. Returning to $\mathbb{P}(\mathbf{x} \in X_2)$,

$$\begin{aligned}
\mathbb{P}(M_{\mathbf{x}} \leq M_{\mathbf{y}}, \forall \mathbf{y} \in Y_2 \cap B_{\mathbb{D}}(\mathbf{x}, R)) &= \sum_{n=0}^{\infty} \frac{\lambda_{\mathbf{x}}^n \mathrm{e}^{-\lambda_{\mathbf{x}}}}{n!} \frac{1}{n+1} \\
&= \frac{\mathrm{e}^{-\lambda_{\mathbf{x}}}}{\lambda_{\mathbf{x}}} \sum_{n=0}^{\infty} \frac{\lambda_{\mathbf{x}}^{n+1}}{(n+1)!} \\
&= \frac{\mathrm{e}^{-\lambda_{\mathbf{x}}}}{\lambda_{\mathbf{x}}} \left[\sum_{n=0}^{\infty} \frac{\lambda_{\mathbf{x}}^n}{n!} - 1\right] \\
&= \frac{\mathrm{e}^{-\lambda_{\mathbf{x}}}}{\lambda_{\mathbf{x}}} (\mathrm{e}^{\lambda_{\mathbf{x}}} - 1) \\
&= \frac{1 - \mathrm{e}^{-\lambda_{\mathbf{x}}}}{\lambda_{\mathbf{x}}} \\
&= \frac{1 - \mathrm{e}^{-\rho\lambda_{\mathbb{D}}(B_{\mathbb{D}}(\mathbf{x}, R))}}{\rho\lambda_{\mathbb{D}}(B_{\mathbb{D}}(\mathbf{x}, R))}.
\end{aligned}$$

Thus returning to the expectation of $N_{X_2}(B)$,

$$\mathbb{E}[N_{X_2}(B)] = \int_B \frac{1 - \mathrm{e}^{-\rho\lambda_{\mathbb{D}}(B_{\mathbb{D}}(\mathbf{x}, R))}}{\lambda_{\mathbb{D}}(B_{\mathbb{D}}(\mathbf{x}, R))} \lambda_{\mathbb{D}}(d\mathbf{x}).$$

□

As we are running simulations based on Matérn II inhibition processes and given that it is a regular process there is a finite maximum number of points that can arise on the surface $\mathbb{D}$. The following corollary to the previous proposition gives the maximum expected value of $N_{X_2}(\mathbb{D})$ for a fixed hard-core distance.

Corollary S2. *Let X be a Matérn II process over $\mathbb{D}$ with hard-core distance R and defined by a Poisson process with constant intensity function ρ. Then,*

$$\sup_{\rho\in\mathbb{R}^+}\mathbb{E}[N_X(\mathbb{D})] = \int_{\mathbb{D}} \frac{1}{\lambda_{\mathbb{D}}(B_{\mathbb{D}}(\mathbf{x}, R))}\lambda_{\mathbb{D}}(d\mathbf{x}).$$

Proof. Notice that since $\rho\lambda_{\mathbb{D}}(B_{\mathbb{D}}(\mathbf{x}, R))$ is always positive this means that $\frac{1-\mathrm{e}^{-\rho\lambda_{\mathbb{D}}(B_{\mathbb{D}}(\mathbf{x},R))}}{\lambda_{\mathbb{D}}(B_{\mathbb{D}}(\mathbf{x},R))} \geq 0$, $\forall\rho \in \mathbb{R}^+$ and $\mathbf{x} \in \mathbb{D}$. Then $\frac{1-\mathrm{e}^{-\rho_1\lambda_{\mathbb{D}}(B_{\mathbb{D}}(\mathbf{x},R))}}{\lambda_{\mathbb{D}}(B_{\mathbb{D}}(\mathbf{x},R))} < \frac{1-\mathrm{e}^{-\rho_2\lambda_{\mathbb{D}}(B_{\mathbb{D}}(\mathbf{x},R))}}{\lambda_{\mathbb{D}}(B_{\mathbb{D}}(\mathbf{x},R))}$, $\forall\rho_1 < \rho_2$ and so the supremum is when ρ is taken to infinity giving the final result. □

The following proposition gives the expected number of points for a Thomas-type process on $\mathbb{D}$.

Proposition S2. *Let X be a Thomas-type process on $\mathbb{D}$ with concentration parameter κ and a constant expected number of offspring, λ, for each parent point. Then,*

$$\mathbb{E}[N_X(\mathbb{D})] = \lambda_{\mathbb{D}}(\mathbb{D})\rho\lambda.$$

Proof. Let $Y = f(X)$, where $f(\mathbf{x}) = \mathbf{x}/||\mathbf{x}||$ then,

$$\begin{aligned}
\mathbb{E}[N_X(\mathbb{D})] &= \mathbb{E}\left[\sum_{\mathbf{c}\in X_p} N_{X_\mathbf{c}}(\mathbb{S}^2)\right] \\
&= \int_{\mathbb{D}} \mathbb{E}[N_{X_\mathbf{c}}(\mathbb{S}^2)]\rho d\mathbf{c} \\
&= \lambda\rho\int_{\mathbb{D}} d\mathbf{c} \\
&= \lambda_{\mathbb{D}}(\mathbb{D})\rho\lambda.
\end{aligned}$$

□

S6.2. Simulation of regular and cluster processes

In order to examine the power of our hypothesis testing procedure, we need to be able to simulate regular and cluster processes outlined here. Much of it is based on simulation of homogeneous Poisson processes on convex shapes. To simulate homogeneous Poisson processes we can first simulate the number of points in the pattern as $\rho\lambda_{\mathbb{D}}(\mathbb{D})$ and then distribute them uniformly across $\mathbb{D}$. In order to distribute the uniformly across $\mathbb{D}$ we can use the rejection sampler outlined by Kopytov and Mityushov [9].

Simulation of Matérn I and II then depend upon removing events in an underlying homogeneous Poisson process based on their distance between the events. This depends on being able to calculate the geodesic distance on a given surface. Assuming this can be achieved, then it is simple to simulate Matérn I and II process using their definitions. For ellipsoidal Matérn I and II processes we use the `geographiclib` [10] available as a `MATLAB` toolbox.

To simulate a Thomas process this can easily be achieved by rejection sampling again. Simulation of the parents is a homogeneous Poisson process. Then, for each parent, we simulate its random number of offspring and then construct a rejection sampler to sample from

$$k(\mathbf{x}_p, \mathbf{x}) = \frac{1}{\chi(\sigma^2)} \exp\left(-\frac{d^2(\mathbf{x}_p, \mathbf{x})}{2\sigma^2}\right),$$

where $\mathbf{x}_p$ is in the parent process, σ is a bandwidth parameter and $\chi(\sigma^2) = \int_{\mathbb{D}} \exp(-d^2(\mathbf{x}_p, \mathbf{x}) /2\sigma^2)\lambda_{\mathbb{D}}(d\mathbf{x})$. Again for an ellipsoidal Thomas process we use `geographiclib` [10] to calculate geodesic distances on the surface of an ellipsoid.

S6.3. Decreasing power

We also see that as a becomes smaller, and therefore c becomes larger a reducing empirical power of our test for both regular and cluster processes, for the same R and κ respectively. This effect could be due to a combination of mapping from the ellipsoid to the sphere and an artefact of the test statistic being proposed over a finite grid of points rather than being consider over the entire range of $[0, \pi]$. To see this consider just the Matérn II process with a fixed hardcore distance R. Further, let us only consider a cross section of the ellipsoid, more

specifically the ellipse such that it's major and minor axis lengths are c and a respectively, see Figure 1. Let us consider the point $(0, a)$ lying on the ellipse and take the point to the right of it which is precisely the hardcore distance R away from it, let us label this point $\mathbf{x}$. To find $\mathbf{x}$ use the parametrisation $(x, y) = (c \cos t, a \sin t)$ for $t \in [0, 2\pi)$. Then we solve the following equation for t to find $\mathbf{x}$,

$$R = \int_t^{\pi/2} (c^2 \sin^2 s + a^2 \cos^2 s)^{1/2} ds.$$

Let us label $\tilde{t}$ as the solution to this equation and we then find $\mathbf{x}$ as $(c \cos \tilde{t}, a \sin \tilde{t})$. Then apply the map onto the unit circle which gives us $(0, a) \mapsto (0, 1)$ and $(c \cos \tilde{t}, a \sin \tilde{t}) \mapsto (\cos \tilde{t}, \sin \tilde{t})$ and so our new hardcore distance on the circle is $R' = \cos^{-1}(\sin \tilde{t})$. It should be noted that this calculation is dependent upon where on an ellipsoid the event of interest, $\mathbf{x}$ is as $B_{\mathbb{D}}(\mathbf{x}, R)$ does not map to $B_{\mathbb{D}}(f(\mathbf{x}), r)$ for some $r > 0$ and where f is our mapping from the ellipsoid to the sphere. Using the example when $a = 0.4000$ we have that $c = 3.1602$ and thus for a hardcore distance of $R = 0.2$, on the cross sectional ellipse we have an effective hardcore distance of $R' = 0.0633$, which is an extremely small hardcore distance and since our finite grid of points is too coarse (points are only separated a distance of 0.02 apart) it results in a loss in power of our test. Furthermore, examining the standardised inhomogeneous K-function in Figure 2 we can further see the effect of our mapping and taking only a finite grid of points along $[0, \pi]$ as the negative deviation reduces as ellipsoid *deforms* further away from the unit sphere. It should be noted though that even though the power of our test reduces as we move further away from the sphere Figure 2 still indicates evidence that of regularity as for small r for all ellipsoids the observed inhomogeneous K-function falls below the simulation envelope. This highlights the importance of a proper examination of graphical representations of functional summary statistics as opposed to the use of formal hypothesis testing [11]. Another consideration would be to potential use a two sided hypothesis testing procedure which may provide greater power when the true underlying process is not CSR.

A similar effect also occurs when examining cluster processes. By mapping the pattern from the ellipsoid to the sphere we will distort the isotropic nature of our offspring density

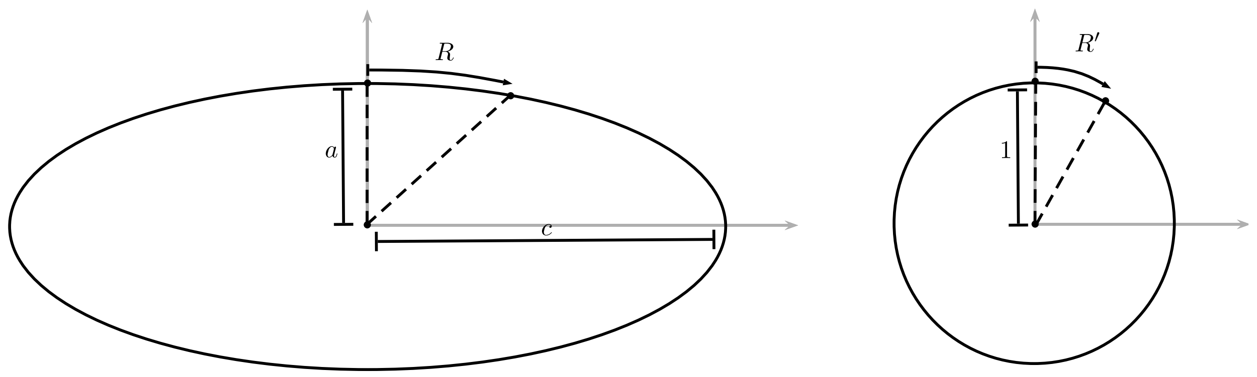

Figure 1: Example of hardcore distance reduction due to mapping to a sphere.

---

relative to its parent. In particular this will cause the cluster size to contract and so if our finite grid of points is too coarse we will struggle to detect deviations away from CSR.

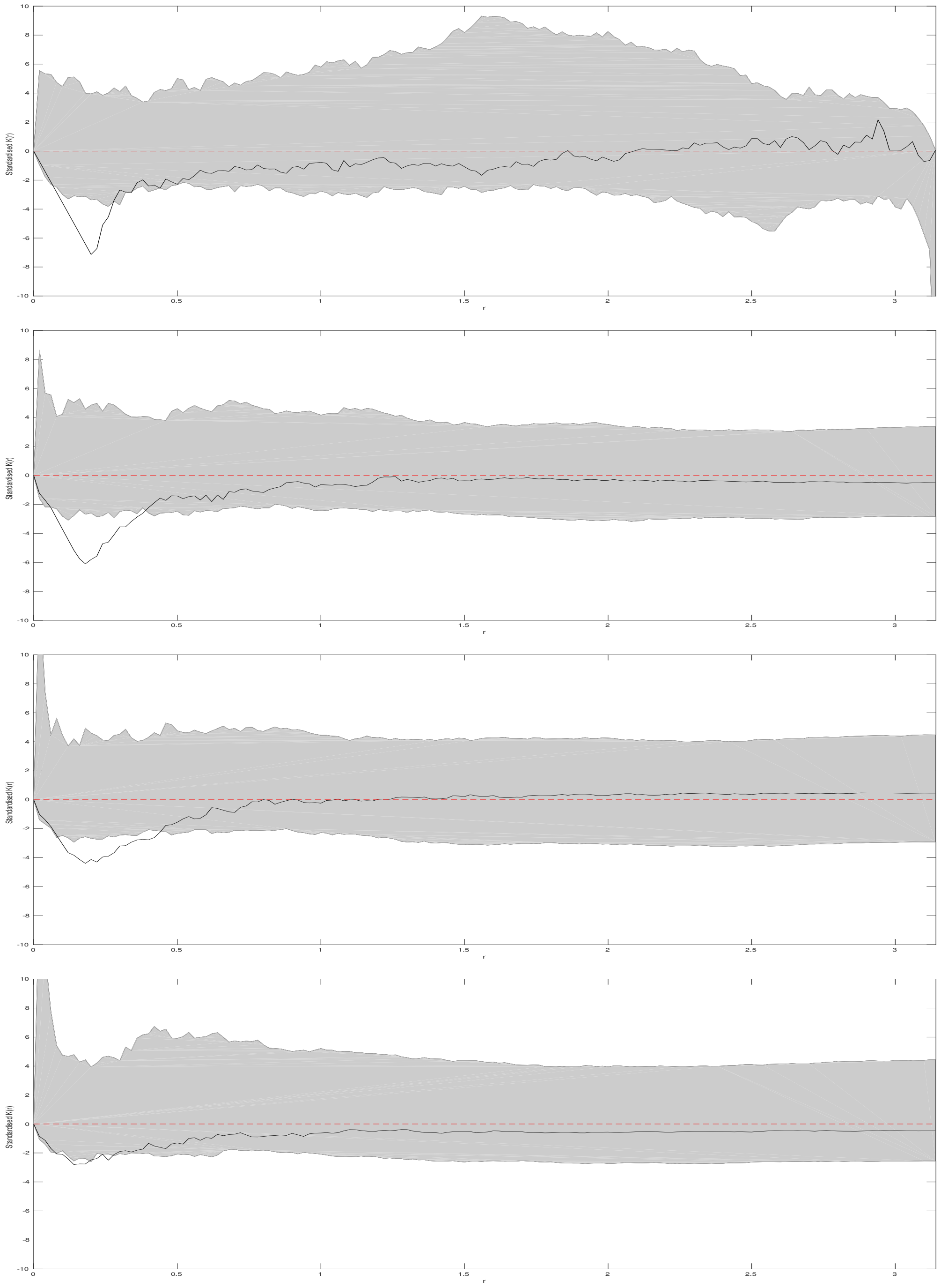

Figure 2: Plots of the standardised inhomogeneous K-function for a Matérn II process for a hardcore distance of $R = 0.2$. From top to bottom $a = 1$ (sphere), $a = 0.8, a = 0.6$ and $a = 0.4$. Black line is the estimated standardised $\hat{K}_{\text{inhom}}(r)$ for our observed data, dashed red line is the theoretical functional summary statistic for a Poisson process, and the grey shaded area represents the simulation envelope from 999 Monte Carlo simulations of Poisson processes fitted to the observed data.

S7. Properties of the estimator of the K_{inhom}-function when ρ is unknown

In this section we shall rederive the expectation and variance of our estimators for $K_{\text{inhom}}(r)$ in the scenario when ρ is unknown. We restate our estimator as,

$$\tilde{K}_{\text{inhom}}(r) = \begin{cases} \frac{\lambda_{\mathbb{D}}^2(\mathbb{D})}{4\pi N_Y(\mathbb{S}^2)(N_Y(\mathbb{S}^2)-1)} \sum_{\mathbf{x}\in Y} \sum_{\mathbf{y}\in Y\setminus\{x\}} \frac{\mathbb{1}[d(\mathbf{x},\mathbf{y})\leq r]}{\tilde{\rho}(\mathbf{x})\tilde{\rho}(\mathbf{y})}, & \text{if } N_Y(\mathbb{S}^2) > 1 \\ 0, & \text{otherwise} \end{cases} \tag{26}$$

where $Y = f(X)$ and

$$\tilde{\rho}(\mathbf{x}) = \begin{cases} l_1(f^{-1}(\mathbf{x}))J_{(1,f^*)}(\mathbf{x})\sqrt{1-\mathbf{x}_1^2-\mathbf{x}_2^2}, & \mathbf{x} \in f(\mathbb{D}_1) \\ \vdots & \\ l_n(f^{-1}(\mathbf{x}))J_{(n,f^*)}(\mathbf{x})\sqrt{1-\mathbf{x}_1^2-\mathbf{x}_2^2}, & \mathbf{x} \in f(\mathbb{D}_n) \end{cases} \tag{27}$$

Then the following theorem gives the properties of our estimator.

Theorem 6. *The bias and variance of $\tilde{K}_{inhom}(r)$ are,*

$$Bias(\tilde{K}_{inhom}(r)) = -P(N_Y(\mathbb{S}^2) \leq 1)2\pi(1-\cos r),$$

and,

$$\begin{aligned} Var(\tilde{K}_{inhom}(r)) &= 4\pi^2(1-\cos r)^2(1-P(N_Y(\mathbb{S}^2)\leq 1))P(N_Y(\mathbb{S}^2)\leq 1) \\ &+ \rho^3\lambda_{\mathbb{D}}^4(\mathbb{D})(1-\cos r)^2\left(\int_{\mathbb{S}^2}\frac{1}{\tilde{\rho}(\mathbf{x})}\lambda_{\mathbb{S}^2}(d\mathbf{x}) - \frac{16\pi^2}{\lambda_{\mathbb{D}}(\mathbb{D})}\right)\mathbb{E}\left[\frac{1}{(N_Y(\mathbb{S}^2)+3)^2(N_Y(\mathbb{S}^2)+2)^2}\right] \\ &+ \frac{\rho^2\lambda_{\mathbb{D}}^4(\mathbb{D})}{8\pi^2}\left(\int_{\mathbb{S}^2}\int_{\mathbb{S}^2}\frac{\mathbb{1}[d(\mathbf{x}_1,\mathbf{x}_2)\leq r]}{\tilde{\rho}(\mathbf{x}_1)\tilde{\rho}(\mathbf{x}_2)}\lambda_{\mathbb{S}^2}(d\mathbf{x}_1)\lambda_{\mathbb{S}^2}(d\mathbf{x}_2) - \frac{64\pi^4(1-\cos r)^2}{\lambda_{\mathbb{D}}^2(\mathbb{D})}\right) \\ &\quad\times\mathbb{E}\left[\frac{1}{(N_Y(\mathbb{S}^2)+2)^2(N_Y(\mathbb{S}^2)+1)^2}\right], \end{aligned} \tag{28}$$

where $\tilde{\rho}(\mathbf{x})$ is given by Equation (27).

Proof. We can rewrite our estimator as,

$$\tilde{K}_{\text{inhom}}(r) = \frac{\mathbb{1}[N_Y(\mathbb{S}^2)>1]\lambda_{\mathbb{D}}^2(\mathbb{D})}{4\pi N_Y(\mathbb{S}^2)(N_Y(\mathbb{S}^2)-1)}\sum_{\mathbf{x}\in Y}\sum_{\mathbf{y}\in Y\setminus\{x\}}\frac{\mathbb{1}[d(\mathbf{x},\mathbf{y})\leq r]}{\tilde{\rho}(\mathbf{x})\tilde{\rho}(\mathbf{y})} \tag{29}$$

Further, note that $N_Y(\mathbb{S}^2) = N_X(\mathbb{D}^2)$, where $f(\mathbf{x}) = \frac{\mathbf{x}}{|\mathbf{x}|}$. We then take expectations (using iterated expectations conditioning on $N_Y(\mathbb{S}^2)$) of $\tilde{K}_{\text{inhom}}(r)$ to get the bias,

$$\begin{aligned}
\mathbb{E}(\tilde{K}_{\text{inhom}}(r)) &= \mathbb{E}\left[\frac{\mathbb{1}[N_Y(\mathbb{S}^2) > 1]\lambda_{\mathbb{D}}^2(\mathbb{D})}{4\pi N_Y(\mathbb{S}^2)(N_Y(\mathbb{S}^2)-1)} \sum_{\mathbf{x}\in Y} \sum_{\mathbf{y}\in Y\setminus\{x\}} \frac{\mathbb{1}[d(\mathbf{x},\mathbf{y}) \le r]}{\tilde{\rho}(\mathbf{x})\tilde{\rho}(\mathbf{y})}\right] \\
&= \frac{\lambda_{\mathbb{D}}^2(\mathbb{D})}{4\pi}\mathbb{E}\left(\frac{\mathbb{1}[N_Y(\mathbb{S}^2) > 1]}{N_Y(\mathbb{S}^2)(N_Y(\mathbb{S}^2)-1)}\mathbb{E}\left[\sum_{\mathbf{x},\mathbf{y}\in Y}^{\neq} \frac{\mathbb{1}[d(\mathbf{x},\mathbf{y}) \le r]}{\tilde{\rho}(\mathbf{x})\tilde{\rho}(\mathbf{y})}\middle| N_Y(\mathbb{S}^2) = n\right]\right) \\
&= \frac{\lambda_{\mathbb{D}}^2(\mathbb{D})}{4\pi}\mathbb{E}\left(\frac{\mathbb{1}[N_Y(\mathbb{S}^2) > 1]}{N_Y(\mathbb{S}^2)(N_Y(\mathbb{S}^2)-1)}\mathbb{E}\left[\sum_{\mathbf{i},\mathbf{j}\in\{1,\dots,n\}}^{\neq} \frac{\mathbb{1}[d(\mathbf{Y}_i,\mathbf{Y}_j) \le r]}{\tilde{\rho}(\mathbf{Y}_i)\tilde{\rho}(\mathbf{Y}_j)}\right]\right) \\
&= \frac{\lambda_{\mathbb{D}}^2(\mathbb{D})}{4\pi}\mathbb{E}\left(\frac{\mathbb{1}[N_Y(\mathbb{S}^2) > 1]N_Y(\mathbb{S}^2)(N_Y(\mathbb{S}^2)-1)}{N_Y(\mathbb{S}^2)(N_Y(\mathbb{S}^2)-1)}\mathbb{E}\left[\frac{\mathbb{1}[d(\mathbf{Y},\mathbf{Y}) \le r]}{\tilde{\rho}(\mathbf{Y})\tilde{\rho}(\mathbf{Y})}\right]\right) \\
&= \frac{\lambda_{\mathbb{D}}^2(\mathbb{D})}{4\pi}P(N_Y(\mathbb{S}^2) > 1)\mathbb{E}\left[\frac{\mathbb{1}[d(\mathbf{Y},\mathbf{Y}) \le r]}{\tilde{\rho}(\mathbf{Y})\tilde{\rho}(\mathbf{Y})}\right] \\
&= \frac{\lambda_{\mathbb{D}}^2(\mathbb{D})}{4\pi}P(N_Y(\mathbb{S}^2) > 1)\int_{\mathbb{S}^2}\int_{\mathbb{S}^2} \frac{\mathbb{1}[d(\mathbf{x},\mathbf{y}) \le r]}{\tilde{\rho}(\mathbf{x})\tilde{\rho}(\mathbf{y})}\frac{\rho^*(\mathbf{x})\rho^*(\mathbf{y})}{\rho^2\lambda_{\mathbb{D}}^2(\mathbb{D})}\lambda_{\mathbb{S}^2}(d\mathbf{x})\lambda_{\mathbb{S}^2}(d\mathbf{y}) \\
&= P(N_Y(\mathbb{S}^2) > 1)2\pi(1-\cos r).
\end{aligned}$$

The bias follows by noting that $P(N_Y(\mathbb{S}^2) > 1) = 1 - P(N_Y(\mathbb{S}^2) \le 1)$. The variance can be calculated using the law of total variance we have,

$$\text{Var}(\tilde{K}_{\text{inhom}}(r)) = \underbrace{\mathbb{E}[\text{Var}(\tilde{K}_{\text{inhom}}(r)|N_Y(\mathbb{S}^2))]}_{(1)} + \underbrace{\text{Var}[\mathbb{E}[\tilde{K}_{\text{inhom}}(r)|N_Y(\mathbb{S}^2)]]}_{(2)}. \tag{30}$$

Considering term (2) first,

$$\mathbb{E}[\tilde{K}_{\text{inhom}}(r)|N_Y(\mathbb{S}^2) = n] = \frac{\mathbb{1}[n > 1]\lambda_{\mathbb{D}}^2(\mathbb{D})}{4\pi n(n-1)}\mathbb{E}\left[\sum_{\mathbf{x},\mathbf{y}\in Y}^{\neq} \frac{\mathbb{1}[d(\mathbf{x},\mathbf{y}) \le r]}{\tilde{\rho}(\mathbf{x})\tilde{\rho}(\mathbf{y})}\middle| N_Y(\mathbb{S}^2) = n\right]$$

Using the fact that given $N_Y(\mathbb{S}^2) = n$, each $\mathbf{x} \in Y$ is independently and identically distributed with density $\rho^*(\mathbf{x})/\mu(\mathbb{D})$, where $\mu(\mathbb{D}) = \rho\lambda_{\mathbb{D}}(\mathbb{D})$, the expectation becomes,

$$\begin{aligned}
&= \frac{\mathbb{1}[n > 1]\lambda_{\mathbb{D}}^2(\mathbb{D})}{4\pi n(n-1)}\mathbb{E}\left[\sum_{i,j\in\{1,\dots,n\}}^{\neq} \frac{\mathbb{1}[d(\mathbf{Y}_i,\mathbf{Y}_j) \le r]}{\tilde{\rho}(\mathbf{Y}_i)\tilde{\rho}(\mathbf{Y}_j)}\middle| N_Y(\mathbb{S}^2) = n\right] \\
&= \frac{\mathbb{1}[n > 1]\lambda_{\mathbb{D}}^2(\mathbb{D})}{4\pi n(n-1)}\sum_{i,j\in\{1,\dots,n\}}^{\neq}\mathbb{E}\left[\frac{\mathbb{1}[d(\mathbf{Y}_i,\mathbf{Y}_j) \le r]}{\tilde{\rho}(\mathbf{Y}_i)\tilde{\rho}(\mathbf{Y}_j)}\middle| N_Y(\mathbb{S}^2) = n\right]
\end{aligned}$$

$$= \frac{\mathbb{1}[n>1]\lambda^2_{\mathbb{D}}(\mathbb{D})}{4\pi n(n-1)} n(n-1)\mathbb{E}\left[\frac{\mathbb{1}[d(\mathbf{X},\mathbf{Y})\le r]}{\tilde{\rho}(\mathbf{X})\tilde{\rho}(\mathbf{Y})}\right]$$
$$= \frac{\mathbb{1}[n>1]\lambda^2_{\mathbb{D}}(\mathbb{D})}{4\pi}\mathbb{E}\left[\frac{\mathbb{1}[d(\mathbf{X},\mathbf{Y})\le r]}{\tilde{\rho}(\mathbf{X})\tilde{\rho}(\mathbf{Y})}\right],$$

where $\mathbf{X}$ and $\mathbf{Y}$ are independent random vectors distributed in $\mathbb{S}^2$ with density $\rho^*(\mathbf{x})/\mu(\mathbb{D})$. Noting that the joint density of $\mathbf{X}$ and $\mathbf{Y}$ is $\rho^*(\mathbf{x})\rho^*(\mathbf{y})/\mu^2(\mathbb{D})$, the expectation becomes,

$$\begin{aligned}
\mathbb{E}[\tilde{K}_{\text{inhom}}(r)|N_Y(\mathbb{S}^2)=n] &= \frac{\mathbb{1}[n>1]\lambda^2_{\mathbb{D}}(\mathbb{D})}{4\pi}\int_{\mathbb{S}^2}\int_{\mathbb{S}^2}\frac{\mathbb{1}[d(\mathbf{x},\mathbf{y})\le r]}{\tilde{\rho}(\mathbf{y})\tilde{\rho}(\mathbf{y})}\frac{\rho^*(\mathbf{x})\rho^*(\mathbf{y})}{\mu^2(\mathbb{D})}\lambda_{\mathbb{S}^2}(d\mathbf{x})\lambda_{\mathbb{S}^2}(d\mathbf{y}) \\
&= \frac{\mathbb{1}[n>1]\lambda^2_{\mathbb{D}}(\mathbb{D})}{4\pi}\frac{1}{\lambda^2_{\mathbb{D}}(\mathbb{D})}\int_{\mathbb{S}^2}\int_{\mathbb{S}^2}\mathbb{1}[d(\mathbf{x},\mathbf{y})\le r]\lambda_{\mathbb{S}^2}(d\mathbf{x})\lambda_{\mathbb{S}^2}(d\mathbf{y}) \\
&= \frac{\mathbb{1}[n>1]}{4\pi}\int_{\mathbb{S}^2}\int_{\mathbb{S}^2}\mathbb{1}[d(\mathbf{x},\mathbf{y})\le r]\lambda_{\mathbb{S}^2}(d\mathbf{x})\lambda_{\mathbb{S}^2}(d\mathbf{y}) \\
&= \mathbb{1}[n>1]2\pi(1-\cos r)
\end{aligned}$$

Hence term (2) is,

$$\begin{aligned}
\text{Var}[\mathbb{E}[\tilde{K}_{\text{inhom}}(r)|N_Y(\mathbb{S}^2)]] &= \text{Var}(\mathbb{1}[N_Y(\mathbb{S}^2)>1]2\pi(1-\cos r)) \\
&= 4\pi^2(1-\cos r)^2\left[\mathbb{E}(\mathbb{1}^2[N_Y(\mathbb{S}^2)>1]) - \mathbb{E}^2(\mathbb{1}[N_Y(\mathbb{S}^2)>1])\right] \\
&= 4\pi^2(1-\cos r)^2\left[P(N_Y(\mathbb{S}^2)>1) - P^2(N_Y(\mathbb{S}^2)>1)\right] \\
&= 4\pi^2(1-\cos r)^2 P(N_Y(\mathbb{S}^2)>1)\left[1-P(N_Y(\mathbb{S}^2)>1)\right] \\
&= 4\pi^2(1-\cos r)^2(1-P(N_Y(\mathbb{S}^2)\le 1))P(N_Y(\mathbb{S}^2)\le 1),
\end{aligned}$$

where, since $N_Y(\mathbb{S}^2)\sim \text{Poisson}(\rho\lambda_{\mathbb{D}}(\mathbb{D}))$,

$$P(N_Y(\mathbb{S}^2)\le 1) = 1 - e^{-\rho\lambda_{\mathbb{D}}(\mathbb{D})} - \rho\lambda_{\mathbb{D}}(\mathbb{D})e^{-\rho\lambda_{\mathbb{D}}(\mathbb{D})}.$$

Now consider term (1), we calculate $\text{Var}(\tilde{K}_{\text{inhom}}(r)|N_Y(\mathbb{S}^2))$,

$$\begin{aligned}
\text{Var}(\tilde{K}_{\text{inhom}}(r)|N_Y(\mathbb{S}^2)=n) &= \text{Var}\left(\frac{\mathbb{1}[N_Y(\mathbb{S}^2)>1]\lambda^2_{\mathbb{D}}(\mathbb{D})}{4\pi N_Y(\mathbb{S}^2)(N_Y(\mathbb{S}^2)-1)}\sum^{\neq}_{\mathbf{x},\mathbf{y}\in Y}\frac{\mathbb{1}[d(\mathbf{x},\mathbf{y})\le r]}{\tilde{\rho}(\mathbf{x})\tilde{\rho}(\mathbf{y})}\middle| N_Y(\mathbb{S}^2)=n\right) \\
&= \frac{\mathbb{1}^2[n>1]\lambda^4_{\mathbb{D}}(\mathbb{D})}{16\pi^2}\text{Var}\left(\frac{1}{n(n-1)}\sum^{\neq}_{\mathbf{x},\mathbf{y}\in Y}\frac{\mathbb{1}[d(\mathbf{x},\mathbf{y})\le r]}{\tilde{\rho}(\mathbf{x})\tilde{\rho}(\mathbf{y})}\middle| N_Y(\mathbb{S}^2)=n\right) \\
&= \frac{\mathbb{1}[n>1]\lambda^4_{\mathbb{D}}(\mathbb{D})}{16\pi^2}\text{Var}\left(\frac{1}{n(n-1)}\sum^{\neq}_{\mathbf{x},\mathbf{y}\in Y}\frac{\mathbb{1}[d(\mathbf{x},\mathbf{y})\le r]}{\tilde{\rho}(\mathbf{x})\tilde{\rho}(\mathbf{y})}\middle| N_Y(\mathbb{S}^2)=n\right)
\end{aligned}$$

$$= \frac{\mathbb{1}[n > 1]\lambda_{\mathbb{D}}^4(\mathbb{D})}{16\pi^2}\text{Var}\left(\frac{1}{n(n-1)}\sum_{i,j\in\{1,\dots,n\}}^{\neq}\frac{\mathbb{1}[d(\mathbf{Y}_i, \mathbf{Y}_j) \le r]}{\tilde{\rho}(\mathbf{Y}_i)\tilde{\rho}(\mathbf{Y}_j)}\right)$$

Here we follow a similar argument to that of Lang and Marcon [12] through U-statistics. Noting that $\frac{\mathbb{1}[d(\mathbf{Y}_i,\mathbf{Y}_j)\le r]}{\tilde{\rho}(\mathbf{Y}_i)\tilde{\rho}(\mathbf{Y}_j)} = \frac{\mathbb{1}[d(\mathbf{Y}_j,\mathbf{Y}_i)\le r]}{\tilde{\rho}(\mathbf{Y}_j)\tilde{\rho}(\mathbf{Y}_i)}$, i.e. it is symmetric in its arguments, we rewrite the summation,

$$\begin{aligned}
\frac{1}{n(n-1)}\sum_{i,j\in\{1,\dots,n\}}^{\neq}\frac{\mathbb{1}[d(\mathbf{Y}_i, \mathbf{Y}_j) \le r]}{\tilde{\rho}(\mathbf{Y}_i)\tilde{\rho}(\mathbf{Y}_j)} &= \frac{1}{n(n-1)}\sum_{1\le i<j\le n}\frac{\mathbb{1}[d(\mathbf{Y}_i, \mathbf{Y}_j) \le r]}{\tilde{\rho}(\mathbf{Y}_i)\tilde{\rho}(\mathbf{Y}_j)} + \frac{\mathbb{1}[d(\mathbf{Y}_j, \mathbf{Y}_i) \le r]}{\tilde{\rho}(\mathbf{Y}_j)\tilde{\rho}(\mathbf{Y}_i)} \\
&= \frac{2}{n(n-1)}\sum_{1\le i<j\le n}\frac{\mathbb{1}[d(\mathbf{Y}_i, \mathbf{Y}_j) \le r]}{\tilde{\rho}(\mathbf{Y}_i)\tilde{\rho}(\mathbf{Y}_j)} \\
&= \binom{n}{2}^{-1}\sum_{1\le i<j\le n}\frac{\mathbb{1}[d(\mathbf{Y}_i, \mathbf{Y}_j) \le r]}{\tilde{\rho}(\mathbf{Y}_i)\tilde{\rho}(\mathbf{Y}_j)}
\end{aligned}$$

This is form of a U-statistic and variances of this class of statistics can be decomposed using the work of Hoeffding [13]. Using the same notation as Hoeffding [13], we define some quantities and derive a number of expectations,

$$\begin{aligned}
U_n &= \binom{n}{2}^{-1}\sum_{1\le i<j\le n}\frac{\mathbb{1}[d(\mathbf{Y}_i, \mathbf{Y}_j) \le r]}{\tilde{\rho}(\mathbf{Y}_i)\tilde{\rho}(\mathbf{Y}_j)} \\
\Phi(\mathbf{y}_1, \mathbf{y}_2) &= \frac{\mathbb{1}[d(\mathbf{y}_1, \mathbf{y}_2) \le r]}{\tilde{\rho}(\mathbf{y}_1)\tilde{\rho}(\mathbf{y}_2)} \\
\Phi_1(\mathbf{y}_1) &\equiv \Phi_1(\mathbf{y}_1, \mathbf{Y}_2) = \mathbb{E}[\Phi(\mathbf{y}_1, \mathbf{Y}_2)] = \mathbb{E}[\Phi(\mathbf{Y}_1, \mathbf{Y}_2)|\mathbf{Y}_1 = \mathbf{y}_1] \\
&= \mathbb{E}\left[\frac{\mathbb{1}[d(\mathbf{y}_1, \mathbf{Y}_2) \le r]}{\tilde{\rho}(\mathbf{y}_1)\tilde{\rho}(\mathbf{Y}_2)}\right] \\
&= \int_{\mathbb{S}^2}\frac{\mathbb{1}[d(\mathbf{y}_1, \mathbf{y}_2) \le r]}{\tilde{\rho}(\mathbf{y}_1)\tilde{\rho}(\mathbf{y}_2)}\frac{\rho^*(\mathbf{y}_2)}{\rho\lambda_{\mathbb{D}}(\mathbb{D})}\lambda_{\mathbb{S}^2}(d\mathbf{y}_2) \\
&= \frac{1}{\tilde{\rho}(\mathbf{y}_1)\lambda_{\mathbb{D}}(\mathbb{D})}\int_{\mathbb{S}^2}\mathbb{1}[d(\mathbf{y}_1, \mathbf{y}_2) \le r]\lambda_{\mathbb{S}^2}(d\mathbf{y}_2) \\
&= \frac{2\pi(1-\cos r)}{\tilde{\rho}(\mathbf{y}_1)\lambda_{\mathbb{D}}(\mathbb{D})} \\
\Phi_2(\mathbf{y}_1, \mathbf{y}_2) &= \mathbb{E}[\Phi(\mathbf{y}_1, \mathbf{y}_2)] = \mathbb{E}[\phi(\mathbf{Y}_1, \mathbf{Y}_2)|\mathbf{Y}_1 = \mathbf{y}_1, \mathbf{Y}_2 = \mathbf{y}_2] = \Phi(\mathbf{y}_1, \mathbf{y}_2) \\
\mathbb{E}[\Phi_1(\mathbf{Y}_1)] &= \int_{\mathbb{S}^2}\frac{2\pi(1-\cos r)}{\tilde{\rho}(\mathbf{y}_1)\lambda_{\mathbb{D}}(\mathbb{D})}\frac{\rho^*(\mathbf{y}_1)}{\rho\lambda_{\mathbb{D}}(\mathbb{D})}\lambda_{\mathbb{S}^2}(d\mathbf{y}_1) \\
&= \frac{4\pi\cdot 2\pi(1-\cos r)}{\lambda_{\mathbb{D}}^2(\mathbb{D})}
\end{aligned}$$

$$
\begin{aligned}
\mathbb{E}[\Phi_2(\mathbf{Y}_1,\mathbf{Y}_2)] &= \int_{\mathbb{S}^2}\int_{\mathbb{S}^2} \frac{\mathbb{1}[d(\mathbf{y}_1,\mathbf{y}_2)\le r]}{\tilde{\rho}(\mathbf{y}_1)\tilde{\rho}(\mathbf{y}_2)} \frac{\rho^*(\mathbf{y}_1)\rho^*(\mathbf{y}_2)}{\rho^2\lambda_{\mathbb{D}}^2(\mathbb{D})}\lambda_{\mathbb{S}^2}(d\mathbf{y}_1)\lambda_{\mathbb{S}^2}(d\mathbf{y}_2)\\
&= \frac{4\pi\cdot 2\pi(1-\cos r)}{\lambda_{\mathbb{D}}^2(\mathbb{D})}\\
\mathbb{E}[\Phi_1^2(\mathbf{Y}_1)] &= \int_{\mathbb{S}^2} \frac{4\pi^2(1-\cos r)^2}{\tilde{\rho}^2(\mathbf{y}_1)\lambda_{\mathbb{D}}^2(\mathbb{D})}\frac{\rho^*(\mathbf{y}_1)}{\rho\lambda_{\mathbb{D}}(\mathbb{D})}\lambda_{\mathbb{S}^2}(d\mathbf{y}_1)\\
&= \frac{4\pi^2(1-\cos r)^2}{\lambda_{\mathbb{D}}^3(\mathbb{D})}\int_{\mathbb{S}^2}\frac{1}{\tilde{\rho}(\mathbf{y}_1)}\lambda_{\mathbb{S}^2}(d\mathbf{y}_1)\\
\mathbb{E}[\Phi_2^2(\mathbf{Y}_1,\mathbf{Y}_2)] &= \int_{\mathbb{S}^2}\int_{\mathbb{S}^2} \frac{\mathbb{1}[d(\mathbf{y}_1,\mathbf{y}_2)\le r]}{\tilde{\rho}^2(\mathbf{y}_1)\tilde{\rho}^2(\mathbf{y}_2)} \frac{\rho^*(\mathbf{y}_1)\rho^*(\mathbf{y}_2)}{\rho^2\lambda_{\mathbb{D}}^2(\mathbb{D})}\lambda_{\mathbb{S}^2}(d\mathbf{y}_1)\lambda_{\mathbb{S}^2}(d\mathbf{y}_2)\\
&= \frac{1}{\lambda_{\mathbb{D}}^2(\mathbb{D})}\int_{\mathbb{S}^2}\int_{\mathbb{S}^2} \frac{\mathbb{1}[d(\mathbf{y}_1,\mathbf{y}_2)\le r]}{\tilde{\rho}(\mathbf{y}_1)\tilde{\rho}(\mathbf{y}_2)}\lambda_{\mathbb{S}^2}(d\mathbf{y}_1)\lambda_{\mathbb{S}^2}(d\mathbf{y}_2)\\
\zeta_1 &= \mathrm{Var}(\Phi_1(\mathbf{Y}_1))\\
&= \mathbb{E}[\Phi_1^2(\mathbf{Y}_1)] - \mathbb{E}^2[\Phi_1(\mathbf{Y}_1)]\\
&= \frac{4\pi^2(1-\cos r)^2}{\lambda_{\mathbb{D}}^3(\mathbb{D})}\int_{\mathbb{S}^2}\frac{1}{\tilde{\rho}(\mathbf{y}_1)}\lambda_{\mathbb{S}^2}(d\mathbf{y}_1) - \frac{16\pi^2\cdot 4\pi^2(1-\cos r)^2}{\lambda_{\mathbb{D}}^4(\mathbb{D})}\\
&= \frac{4\pi^2(1-\cos r)^2}{\lambda_{\mathbb{D}}^3(\mathbb{D})}\left(\int_{\mathbb{S}^2}\frac{1}{\tilde{\rho}(\mathbf{y}_1)}\lambda_{\mathbb{S}^2}(d\mathbf{y}_1) - \frac{16\pi^2}{\lambda_{\mathbb{D}}(\mathbb{D})}\right)\\
\zeta_2 &= \mathrm{Var}(\Phi_2(\mathbf{Y}_1,\mathbf{Y}_2))\\
&= \mathbb{E}[\Phi_2^2(\mathbf{Y}_1,\mathbf{Y}_2)] - \mathbb{E}^2[\Phi_2(\mathbf{Y}_1,\mathbf{Y}_2)]\\
&= \frac{1}{\lambda_{\mathbb{D}}^2(\mathbb{D})}\int_{\mathbb{S}^2}\int_{\mathbb{S}^2} \frac{\mathbb{1}[d(\mathbf{y}_1,\mathbf{y}_2)\le r]}{\tilde{\rho}(\mathbf{y}_1)\tilde{\rho}(\mathbf{y}_2)}\lambda_{\mathbb{S}^2}(d\mathbf{y}_1)\lambda_{\mathbb{S}^2}(d\mathbf{y}_2) - \frac{16\pi^2\cdot 4\pi^2(1-\cos r)^2}{\lambda_{\mathbb{D}}^4(\mathbb{D})}\\
&= \frac{1}{\lambda_{\mathbb{D}}^2(\mathbb{D})}\left(\int_{\mathbb{S}^2}\int_{\mathbb{S}^2} \frac{\mathbb{1}[d(\mathbf{y}_1,\mathbf{y}_2)\le r]}{\tilde{\rho}(\mathbf{y}_1)\tilde{\rho}(\mathbf{y}_2)}\lambda_{\mathbb{S}^2}(d\mathbf{y}_1)\lambda_{\mathbb{S}^2}(d\mathbf{y}_2) - \frac{64\pi^4(1-\cos r)^2}{\lambda_{\mathbb{D}}^2(\mathbb{D})}\right)
\end{aligned}
$$

Then using the variance derived by Hoeffding [13] for U-statistics, the variance of our U_n can be decomposed as,

$$
\begin{aligned}
\mathrm{Var}\left(U_n\right) &= \binom{n}{2}^{-1}\sum_{k=1}^{2}\binom{2}{k}\binom{n-2}{2-k}\zeta_k\\
&= \frac{4(n-2)}{n(n-1)}\zeta_1 + \frac{2}{n(n-1)}\zeta_2
\end{aligned}
$$

Then,

$$\mathrm{Var}(\tilde{K}_{\mathrm{inhom}}(r)|N_Y(\mathbb{S}^2)=n) = \frac{\mathbb{1}[n>1]\lambda_{\mathbb{D}}^4(\mathbb{D})}{16\pi^2}\left(\frac{4(n-2)}{n(n-1)}\zeta_1 + \frac{2}{n(n-1)}\zeta_2\right)$$
$$= \lambda_{\mathbb{D}}(\mathbb{D})(1-\cos r)^2\left(\int_{\mathbb{S}^2}\frac{1}{\tilde{\rho}(\mathbf{y}_1)}\lambda_{\mathbb{S}^2}(d\mathbf{y}_1) - \frac{16\pi^2}{\lambda_{\mathbb{D}}(\mathbb{D})}\right)\cdot\frac{\mathbb{1}[n>1](n-2)}{n(n-1)}$$
$$+ \frac{\lambda_{\mathbb{D}}^2(\mathbb{D})}{8\pi^2}\left(\int_{\mathbb{S}^2}\int_{\mathbb{S}^2}\frac{\mathbb{1}[d(\mathbf{y}_1,\mathbf{y}_2)\le r]}{\tilde{\rho}(\mathbf{y}_1)\tilde{\rho}(\mathbf{y}_2)}\lambda_{\mathbb{S}^2}(d\mathbf{y}_1)\lambda_{\mathbb{S}^2}(d\mathbf{y}_2) - \frac{64\pi^4(1-\cos r)^2}{\lambda_{\mathbb{D}}^2(\mathbb{D})}\right)\frac{\mathbb{1}[n>1]}{n(n-1)}$$

Taking expectations gives,

$$\mathbb{E}[\mathrm{Var}(\tilde{K}_{\mathrm{inhom}}(r)|N_Y(\mathbb{S}^2))]$$
$$= \lambda_{\mathbb{D}}(\mathbb{D})(1-\cos r)^2\left(\int_{\mathbb{S}^2}\frac{1}{\tilde{\rho}(\mathbf{y}_1)}\lambda_{\mathbb{S}^2}(d\mathbf{y}_1) - \frac{16\pi^2}{\lambda_{\mathbb{D}}(\mathbb{D})}\right)\mathbb{E}\left[\frac{\mathbb{1}[N_Y(\mathbb{S}^2)>1](N_Y(\mathbb{S}^2)-2)}{N_Y(\mathbb{S}^2)(N_Y(\mathbb{S}^2)-1)}\right]$$
$$+ \frac{\lambda_{\mathbb{D}}^2(\mathbb{D})}{8\pi^2}\left(\int_{\mathbb{S}^2}\int_{\mathbb{S}^2}\frac{\mathbb{1}[d(\mathbf{y}_1,\mathbf{y}_2)\le r]}{\tilde{\rho}(\mathbf{y}_1)\tilde{\rho}(\mathbf{y}_2)}\lambda_{\mathbb{S}^2}(d\mathbf{y}_1)\lambda_{\mathbb{S}^2}(d\mathbf{y}_2) - \frac{64\pi^4(1-\cos r)^2}{\lambda_{\mathbb{D}}^2(\mathbb{D})}\right)\mathbb{E}\left[\frac{\mathbb{1}[N_Y(\mathbb{S}^2)>1]}{N_Y(\mathbb{S}^2)(N_Y(\mathbb{S}^2)-1)}\right]$$

The expectations can be simplified as follows and defining $\lambda = \rho\lambda_{\mathbb{D}}(\mathbb{D})$,

$$\begin{aligned}
\mathbb{E}\left[\frac{\mathbb{1}[N_Y(\mathbb{S}^2)>1](N_Y(\mathbb{S}^2)-2)}{N_Y(\mathbb{S}^2)(N_Y(\mathbb{S}^2)-1)}\right] &= \sum_{n=0}^{\infty}\frac{\mathbb{1}[n>1](n-2)}{n(n-1)}\frac{\lambda^n e^{-\lambda}}{n!}\\
&= \sum_{n=3}^{\infty}\frac{(n-2)}{n(n-1)}\frac{\lambda^n e^{-\lambda}}{n!}\\
&= \sum_{n=3}^{\infty}\frac{1}{n^2(n-1)^2}\frac{\lambda^n e^{-\lambda}}{(n-3)!}\\
&= \sum_{n=0}^{\infty}\frac{1}{(n+3)^2(n+2)^2}\frac{\lambda^{n+3}e^{-\lambda}}{n!}\\
&= \lambda^3\sum_{n=0}^{\infty}\frac{1}{(n+3)^2(n+2)^2}\frac{\lambda^n e^{-\lambda}}{n!}\\
&= \lambda^3\mathbb{E}\left[\frac{1}{(N_Y(\mathbb{S}^2)+3)^2(N_Y(\mathbb{S}^2)+2)^2}\right]
\end{aligned}$$

Similarly the other expectation is,

$$\mathbb{E}\left[\frac{\mathbb{1}[N_Y(\mathbb{S}^2)>1]}{N_Y(\mathbb{S}^2)(N_Y(\mathbb{S}^2)-1)}\right] = \lambda^2\mathbb{E}\left[\frac{1}{(N_Y(\mathbb{S}^2)+2)^2(N_Y(\mathbb{S}^2)+1)^2}\right],$$

and so,

$$\begin{aligned}
&\mathbb{E}[\mathrm{Var}(\tilde{K}_{\mathrm{inhom}}(r)|N_Y(\mathbb{S}^2))] \\
&\quad = \rho^3\lambda_{\mathbb{D}}^4(\mathbb{D})(1-\cos r)^2\left(\int_{\mathbb{S}^2}\frac{1}{\tilde{\rho}(\mathbf{y}_1)}\lambda_{\mathbb{S}^2}(d\mathbf{y}_1) - \frac{16\pi^2}{\lambda_{\mathbb{D}}(\mathbb{D})}\right)\mathbb{E}\left[\frac{1}{(N_Y(\mathbb{S}^2)+3)^2(N_Y(\mathbb{S}^2)+2)^2}\right] \\
&\quad + \frac{\rho^2\lambda_{\mathbb{D}}^4(\mathbb{D})}{8\pi^2}\left(\int_{\mathbb{S}^2}\int_{\mathbb{S}^2}\frac{\mathbb{1}[d(\mathbf{y}_1,\mathbf{y}_2)\le r]}{\tilde{\rho}(\mathbf{y}_1)\tilde{\rho}(\mathbf{y}_2)}\lambda_{\mathbb{S}^2}(d\mathbf{y}_1)\lambda_{\mathbb{S}^2}(d\mathbf{y}_2) - \frac{64\pi^4(1-\cos r)^2}{\lambda_{\mathbb{D}}^2(\mathbb{D})}\right) \\
&\qquad\quad \times\mathbb{E}\left[\frac{1}{(N_Y(\mathbb{S}^2)+2)^2(N_Y(\mathbb{S}^2)+1)^2}\right].
\end{aligned}$$

Combining everything gives the variance of $\tilde{K}_{\mathrm{inhom}}(r)$,

$$\begin{aligned}
&\mathrm{Var}(\tilde{K}_{\mathrm{inhom}}(r)) = 4\pi^2(1-\cos r)^2(1-P(N_Y(\mathbb{S}^2)\le 1))P(N_Y(\mathbb{S}^2)\le 1) \\
&\quad + \rho^3\lambda_{\mathbb{D}}^4(\mathbb{D})(1-\cos r)^2\left(\int_{\mathbb{S}^2}\frac{1}{\tilde{\rho}(\mathbf{y}_1)}\lambda_{\mathbb{S}^2}(d\mathbf{y}_1) - \frac{16\pi^2}{\lambda_{\mathbb{D}}(\mathbb{D})}\right)\mathbb{E}\left[\frac{1}{(N_Y(\mathbb{S}^2)+3)^2(N_Y(\mathbb{S}^2)+2)^2}\right] \\
&\quad + \frac{\rho^2\lambda_{\mathbb{D}}^4(\mathbb{D})}{8\pi^2}\left(\int_{\mathbb{S}^2}\int_{\mathbb{S}^2}\frac{\mathbb{1}[d(\mathbf{y}_1,\mathbf{y}_2)\le r]}{\tilde{\rho}(\mathbf{y}_1)\tilde{\rho}(\mathbf{y}_2)}\lambda_{\mathbb{S}^2}(d\mathbf{y}_1)\lambda_{\mathbb{S}^2}(d\mathbf{y}_2) - \frac{64\pi^4(1-\cos r)^2}{\lambda_{\mathbb{D}}^2(\mathbb{D})}\right) \\
&\qquad\quad \times\mathbb{E}\left[\frac{1}{(N_Y(\mathbb{S}^2)+2)^2(N_Y(\mathbb{S}^2)+1)^2}\right]
\end{aligned}$$

□

In order to construct estimates of $\mathrm{Var}(\tilde{K}_{\mathrm{inhom}}(r))$ we need estimators for $P(N_Y(\mathbb{S}^2)\le 1)$. The following lemma helps us do this.

Lemma 2. *Let $N \sim$ Poisson(λ) and $k, p \in \mathbb{N}$. Define the following random variable,*

$$R = \frac{N!e^{N-k}}{(N-k)!(e+p)^N}.$$

Then R is ratio-unbiased for $\lambda^k e^{-p\lambda}$.

Proof. Define $S = N!e^{N-k}/(N-k)!$ and $T = (e+p)^N$, then $R = S/T$. Then,

$$\begin{aligned}
\mathbb{E}[S] &= \sum_{n=0}^{\infty}\frac{n!e^{n-k}}{(n-k)!}\frac{\lambda^n e^{-\lambda}}{n!} \\
&= e^{-k}e^{-\lambda}\sum_{n=k}^{\infty}\frac{(e\lambda)^n}{(n-k)!} \\
&= e^{-k}e^{-\lambda}\sum_{l=0}^{\infty}\frac{(e\lambda)^{l+k}}{l!}
\end{aligned}$$

$$
\begin{aligned}
&= e^{-k} e^{-\lambda} (e\lambda)^k \sum_{l=0}^{\infty} \frac{(e\lambda)^l}{l!} \\
&= e^{-k} e^{-\lambda} (e\lambda)^k e^{e\lambda} \\
&= \lambda^k e^{\lambda(e-1)}.
\end{aligned}
$$

Further,

$$
\begin{aligned}
\mathbb{E}[T] &= \sum_{n=0}^{\infty} (e+p)^n \frac{\lambda^n e^{-\lambda}}{n!} \\
&= e^{-\lambda} \sum_{n=0}^{\infty} \frac{(\lambda(e+p))^n}{n!} \\
&= e^{-\lambda} e^{\lambda(e+p)} \\
&= e^{\lambda(e+p-1)}.
\end{aligned}
$$

Therefore,

$$
\frac{\mathbb{E}[S]}{\mathbb{E}[T]} = \frac{\lambda^k e^{\lambda(e-1)}}{e^{\lambda(e+p-1)}} = \lambda^k e^{-p\lambda}
$$

□

References

[1] S. D. White, The hierarchy of correlation functions and its relation to other measures of galaxy clustering, Monthly Notices of the Royal Astronomical Society 186 (1979) 145–154.

[2] J.-F. Coeurjolly, J. Møller, R. Waagepetersen, A tutorial on Palm distributions for spatial point processes, International Statistical Review 85 (2017) 404–420.

[3] M. N. van Lieshout, A J-function for inhomogeneous point processes, Statistica Neerlandica 65 (2011) 183–201.

[4] J. F. C. Kingman, Poisson Processes, Oxford University Press, 1993.

[5] J. Møller, R. P. Waagepetersen, Statistical Inference and Simulation for Spatial Point Processes, CRC Press, Florida, 2004.

[6] T. Lawrence, A. Baddeley, R. K. Milne, G. Nair, Point pattern analysis on a region of a sphere, Stat 5 (2016) 144–157.

[7] J. Møller, E. Rubak, Functional summary statistics for point processes on the sphere with an application to determinantal point processes, Spatial Statistics 18 (2016) 4–23.

[8] K. Wolter, Introduction to variance estimation, Springer Science & Business Media, 2007.

[9] N. P. Kopytov, E. A. Mityushov, The method for uniform distribution of points on surfaces in multi-dimensional Euclidean space, Intellectual Archive (2012).

[10] C. Karney, geographiclib, MATLAB Central File Exchange, 2020. URL: `https://www.mathworks.com/matlabcentral/fileexchange/50605-geographiclib`.

[11] P. J. Diggle, Statistical Analysis of Spatial Point Patterns, 2nd ed., Oxford University Press, New York, 2003.

[12] G. Lang, E. Marcon, Testing randomness of spatial point patterns with the Ripley statistic, ESAIM: Probability and Statistics 17 (2013) 767–788.

[13] W. Hoeffding, A Class of Statistics with Asymptotically Normal Distribution, Springer New York, New York, NY, 1992, pp. 308–334.
